# The *Echinococcus canadensis* (G7) genome: a key knowledge of parasitic platyhelminth human diseases

**DOI:** 10.1186/s12864-017-3574-0

**Published:** 2017-02-27

**Authors:** Lucas L. Maldonado, Juliana Assis, Flávio M. Gomes Araújo, Anna C. M. Salim, Natalia Macchiaroli, Marcela Cucher, Federico Camicia, Adolfo Fox, Mara Rosenzvit, Guilherme Oliveira, Laura Kamenetzky

**Affiliations:** 1 IMPaM, CONICET, Facultad de Medicina, Universidad de Buenos Aires, Ciudad Autónoma de Buenos Aires, Argentina; 2 Genomics and Computational Biology Group, René Rachou Research Center, Oswaldo Cruz Foundation, Belo Horizonte, Brazil; 3 Instituto Tecnológico Vale, Belém, Brazil

**Keywords:** *Echinococcus* genome, SNPs, Drug targets, Helminth parasites, Comparative genomics

## Abstract

**Background:**

The parasite *Echinococcus canadensis* (G7) (phylum Platyhelminthes, class Cestoda) is one of the causative agents of echinococcosis. Echinococcosis is a worldwide chronic zoonosis affecting humans as well as domestic and wild mammals, which has been reported as a prioritized neglected disease by the World Health Organisation. No genomic data, comparative genomic analyses or efficient therapeutic and diagnostic tools are available for this severe disease. The information presented in this study will help to understand the peculiar biological characters and to design species-specific control tools.

**Results:**

We sequenced, assembled and annotated the 115-Mb genome of *E. canadensis* (G7). Comparative genomic analyses using whole genome data of three *Echinococcus* species not only confirmed the status of *E. canadensis* (G7) as a separate species but also demonstrated a high nucleotide sequences divergence in relation to *E. granulosus* (G1). The *E. canadensis* (G7) genome contains 11,449 genes with a core set of 881 orthologs shared among five cestode species. Comparative genomics revealed that there are more single nucleotide polymorphisms (SNPs) between *E. canadensis* (G7) and *E. granulosus* (G1) than between *E. canadensis* (G7) and *E. multilocularis.* This result was unexpected since *E. canadensis* (G7) and *E. granulosus* (G1) were considered to belong to the species complex *E. granulosus sensu lato.* We described SNPs in known drug targets and metabolism genes in the *E. canadensis* (G7) genome. Regarding gene regulation, we analysed three particular features: CpG island distribution along the three *Echinococcus* genomes, DNA methylation system and small RNA pathway. The results suggest the occurrence of yet unknown gene regulation mechanisms in *Echinococcus*.

**Conclusions:**

This is the first work that addresses *Echinococcus* comparative genomics. The resources presented here will promote the study of mechanisms of parasite development as well as new tools for drug discovery. The availability of a high-quality genome assembly is critical for fully exploring the biology of a pathogenic organism. The *E. canadensis* (G7) genome presented in this study provides a unique opportunity to address the genetic diversity among the genus *Echinococcus* and its particular developmental features. At present, there is no unequivocal taxonomic classification of *Echinococcus* species; however, the genome-wide SNPs analysis performed here revealed the phylogenetic distance among these three *Echinococcus* species. Additional cestode genomes need to be sequenced to be able to resolve their phylogeny.

**Electronic supplementary material:**

The online version of this article (doi:10.1186/s12864-017-3574-0) contains supplementary material, which is available to authorized users.

## Background

Tapeworms belong to one of the three major groups of worms that parasitize humans, the other two comprises flukes (Trematoda) and round worms (Nematoda). Despite their public health importance, genome-wide data are currently available only for a few parasitic platyhelminth species including *Schistosoma mansoni* [1], *Schistosoma japonicum* [2], *Schistosoma haematobium* [3]*, Clonorchis sinensis* [4], and the tapeworms *Taenia solium*, *Hymenolepis microstoma*, *Echinococcus multilocularis*, *Echinococcus granulosus* (G1) [5, 6] and *Spirometra erinaceieuropaei* [7]. Recently, the 50 Helminth Genomes Initiative headed by the Wellcome Trust Sanger Institute provided several additional draft genomes of nematodes, cestodes and trematodes (ftp://ftp.sanger.ac.uk/pub/project/pathogens/HGI/).

Cystic hydatid disease is a zoonosis caused by *Echinococcus granulosus sensu lato* species complex which is associated with poverty and poor hygiene practices, particularly in livestock-raising communities [8]. It is a preventable condition that is recognized by the World Health Organisation as a “neglected” disease. It has been estimated that 1–3.6 million disability-adjusted life years are lost worldwide due to human cystic echinococcosis [9] and that up to $2 billion are lost annually in the livestock industry [10]. *E. granulosus s.l.* has a complex life cycle, including intermediate hosts (domesticated or wild ungulates), where the hydatid cyst develops by asexual reproduction, and definitive hosts (domesticated or wild canids), where adult flatworms develop by sexual reproduction. Humans are accidentally infected by the ingestion of tapeworm eggs in contaminated food or water, or by direct contact with definitive hosts. Hydatid cysts develop mainly in the liver (65%) and lungs (25%), and less frequently in muscles, spleen, bones, kidneys, brain, eyes, heart and pancreas [11]. The rupture of an hydatid cyst and the sequelae of rupture are named secondary hydatid disease and are more important than the mass effect of hydatid cysts, mostly in the brain, where the mass effect has severe consequences.

*Echinococcus granulosus s.l.* was initially described as being composed of ten genotypes (G1 to G10) [12]. In recent years, mitochondrial phylogenetic analyses allowed to classify most of the genotypes as new species [13]. The new classification determines that *E. granulosus s. l.* is composed of five species: *Echinococcus granulosus sensu stricto* (G1/G2/G3), *Echinococcus equinus* (G4), *Echinococcus ortleppi* (G5), *Echinococcus canadensis* (G6/G7/G8/G10) and *Echinococcus felidis*. All of them cause unilocular echinococcosis and are macroscopically indistinguishable at the larval stage (hydatid cysts). For many years they were considered to be the same species; nevertheless, some species show clear differences such as intermediate host infectivity, antigenic profile and infectivity to humans [12]. In human beings, *E. granulosus s. s.* and *E. canadensis* are the most prevalent species, representing ~77 and ~22% of total worldwide cases reported for each species, respectively [14, 15]. At present, there is no clear link between *Echinococcus* genetic diversity and human infection features. Some reports suggest that human infections caused by *E. canadensis* (G7) presented smaller liver cysts than those caused by *E. granulosus s. s.* (G1) [16], and that *E. canadensis* (G6) is found more frequently in the brain [17]. In previous studies of *E. canadensis* isolates from South America we have demonstrated that G6 and G7 genotypes are genetically indistinguishable by most of the molecular markers employed [18–22]. Particularly, *E. canadensis* (G7) was shown to differ from *E. granulosus* s. s. (G1) in the rate of development in the definitive host [23]. Recently, we demonstrated that *E. canadensis* (G7) protoscoleces are unable to establish an infection in the murine model [24]. However, very little is known about the factors that determine its host specificity or developmental differences. In order to address biological differences among *E. granulosus s. l.* species, systematic experiments need to be performed involving all of the species. This approach is very difficult to carry out with *Echinococcus* species since biological material from natural infections is difficult to sample and it is not possible to obtain the complete life cycle neither in vivo nor in vitro.

Regard the disease control technics, albendazole is the only drug recommended by the World Health Organisation to treat cystic echinococcosis (http://www.who.int/mediacentre/factsheets/fs377/en/, [25]). However, this drug has low dissolution, low absorption and several side-effects [26]. In addition, resistance and/or differential efficacy can potentially arise in some *Echinococcus* species; therefore new effective anti-echinococcosis drugs need to be urgently developed. Hence, knowledge of the universe of potential gene product targets is essential.

Despite its biological and public health importance, no genome-wide data have yet been produced for *Echinococcus canadensis* (G7). In this work we are describing the genome sequencing and annotation of *E. canadensis* (G7) genome. We are showing comparative genomics and genome-wide SNPs analyses performed among three *Echinococcus* species which revealed particular SNPs sites in known drug targets and metabolism genes. Specific cestode gene families were identified based on large-scale orthology comparisons of gene families across the phyla. In addition, a curated list of potential new drug targets is presented. Finally, phylogenetic analyses based on different approaches allowed to determine the genetic distance among *Echinococcus* species. These data contribute to the growing global resources that allow new treatments and to understand the biology and particular features of these parasites.

## Results

### The genome of *Echinococcus canadensis* (G7) and gene annotation

**Fig. 1:**
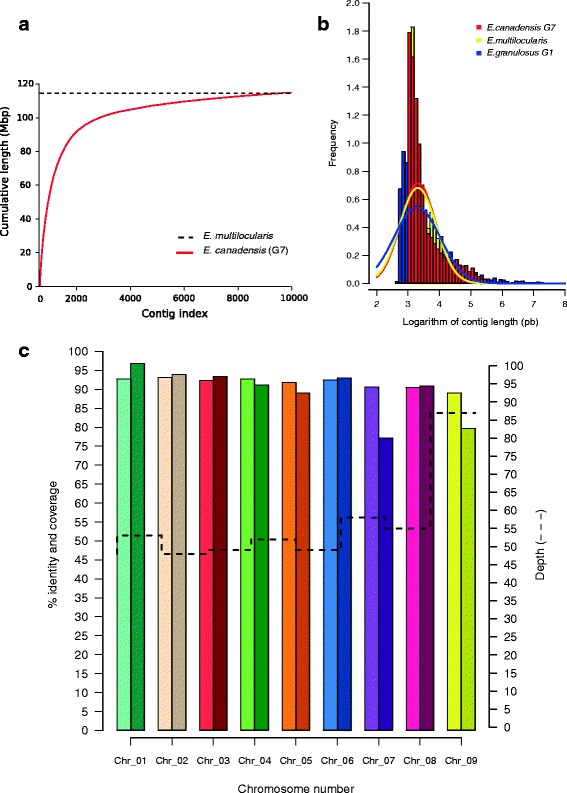
Statistic measures of quality assembly. **a** Cumulative length distribution using *E. multilocularis* assembly as reference genome. **b** *Echinococcus canadensis* (G7) contig length distribution. The histogram represents the frequency of contigs per log contig length (bp). *Lines* indicate normal distribution of log contig length. **c** Identity, coverage (*bars*) and depth coverage (*dashedline*) of *E. canadensis* (G7) contigs on *E. multilocularis* chromosomes

The *E. canadensis* (G7) genome sequence was assembled from a combination of two Illumina libraries. High-quality genomic DNA was purified from a large unilocular cyst. Polymerase chain reaction (PCR) amplification of cytochrome oxidase 1 (COX1) followed by direct sequencing confirmed the (G7) genotype. Five de novo assembly strategies were performed and the best assembly of *E. canadensis* (G7) genome was chosen based on quality metrics such as N50, deep coverage, number of contigs, %GC and the coverage of the *E. multilocularis* genome. The best assembly was achieved using SPAdes [27] (Fig. 1 and Additional file 1: 1.1). After removing putative non target contigs (lower than 1 kb), the genome was composed of 9326 contigs whose quality assembly parameters were: N50 74.6 kb, 55× depth coverage (Table 1). The *E. canadensis* (G7) genome assembly comprised 115 Mb with 41.86% of GC content. Mapping and ordering of *E. canadensis* (G7) contigs on the *E. multilocularis* chromosomes resulted in 77.1–96.9% of coverage and 89.1–93.1% of identity (Fig. 1c). The total number of contigs included a 13,719-bp contig length belonging to the mitochondrial genome, with 85% of coverage, 95% of genes and 99.9% of nucleotide identity in relation to the *E. canadensis* (G7) mitochondrial genome, currently deposited in the GenBank under the accession number AB235847 (Additional file 1: 1.2). The Core Eukaryotic Genes Mapping Approach (CEGMA) software [28] was used to assess the completeness of the genome. The gene space was estimated to be >85.08% complete and the most conservative CEGMA reference gene sets were recovered up to >95% rate in the *E. canadensis* (G7) genome. Protein-coding genes were predicted using the MAKER2 software [29] along with species-specific gene models of *E. multilocularis* and *E. granulosus* (G1) and transcriptomic data of *E. canadensis* (G7). A total of 11,449 gene model predictions were obtained comprising a gene density of 12.82 genes per Mb (Table 1). A total of 6842 proteins were functionally annotated as follows: 6205 proteins were associated with InterPro2GO terms, 493 were associated by using a cestode-specific orthology group and 144 were annotated by reciprocal BLAST against UniProt database. The remaining 4607 proteins were classified as hypothetical (Additional file 1: 1.3 and 1.4). The frequency of GO (Gene Ontology) terms obtained for the annotated proteins is presented in Additional file 2A. The two main categories found were binding (GO:0005488) and catalytic activity (GO:0003824), which is in accordance with the GO terms frequency observed in other cestode genomes [30]. In addition, a total of 4202 proteins of *E. canadensis* (G7) were assigned to one or more KO (KEGG (Kyoto Encyclopedia of Genes and Genomes) Orthology) identifiers classified into 31 categories of the main 5 KEGG pathways (Additional file 3: 3.1 and 3.2). We found that *E. canadensis* (G7) had 656 KO terms associated with metabolism, grouped in 11 pathways. We found ~ 80% of KEGG pathways expected in *Echinococcus.* Pathways such as de novo synthesis of nucleotide bases, amino acids and lipids seem to have been lost, which is in accordance with previous results obtained by Zhang et al. and Tsai et al. [5, 6], and reinforces the dependence of *Echinococcus* on its host metabolites (Additional file 3: 3.3). Regarding non-coding genes, a total of 39 microRNA genes were identified in agreement with our previous results [31]. Also 5 18S, 3 5.8S and 2 28S rRNA genes were found in addition to 124 tRNA genes with 76.8–100% of identity to previously described tRNAs sequences (Additional file 1: 1.5, 1.6 and 1.7). A total of 21 tRNA gene clusters with up to 6 tRNA genes were identified, according to what has been reported for nematodes and mammalian genomes [32].

**Table 1:** Genome-wide statistics for the *Echinococcus canadensis* (G7) assembly and gene predictions

Genome statistics	
Size of genome (Mb)	115
GC content (%)	42
Number of contigs	9326
N50 (Kb)	75
Largest contig (Kb)	574
Deep coverage	55×
Number of predicted genes	11,449
Gene density per Mb	13
Length of proteome (amino acids)	4,915,068
Maximum protein length (amino acids)	7886
Average protein length (amino acids)	440
Average exon length (bp)	219
Median exon length (bp)	159
Average exons per transcript	6
Median exons per transcript	4
Total length of contained introns (Kb)	40,117
Average intron length (bp)	714
Median intron length (bp)	273

### Repetitive elements

*E. canadensis* (G7) repeat sequences (E.canG7_rep), including simple repeats, interspersed repeats and satellite DNAs, were identified in the assembled genome using RepeatModeler [33] and an in-house flatworm repetitive database. We found that the repeat content of the parasite genome was 7.9% and included well-known repetitive elements, such as *E. canadensis* (G7) genotype TREG element described in previous studies by us [18, 19]. To perform comparative genomic analyses, we selected a subset of sequences with more than 50% of coverage of the consensus repetitive sequences. This highly reliable set of repetitive elements was composed of 213 sequences and comprised 2.4% of the *E. canadensis* (G7) genome. Repetitive sequences were classified as DNA transposons (32.4%), Long terminal repeat (LTR) retrotransposons (27.7%), Long Interspersed Nuclear Elements (LINEs) (19.3%), Short Interspersed Nuclear Elements (SINEs) (0.9%) and unknown class (19.7%) (Additional file 1: 1.8 and 1.9). Moreover, 13 microsatellite sequences representing 20 loci in the *E. canadensis* (G7) genome were identified as tandemly repeated multi-loci microsatellite EmsB, which have been used to study the genetic diversity of the genus *Echinococcus* [34, 35]. The identity of the repetitive elements between *E. canadensis* (G7) and *E. granulosus* (G1) ranged from 65.5% to 100%, whereas between *E. canadensis* (G7) and *E. multilocularis* ranged from 57% to 100%. The most frequent E.canG7_rep sequence identified was the E.canG7_Brep (548 loci), which had high similarity with *E. granulosus* (G1) EgBRep repetitive DNA element (98.93% of identity) [36] (GenBank: ×67152.1). This SINE sequence had less conservation in *E. multilocularis* (82.86% of identity). Also, E.canG7_Brep sequences seem to be specific for flatworms since positive hits were found in *Hymenolepis microstoma*, *Taenia solium*, *Schistosoma mansoni* and *Schistosoma japonicum*, and no sequence similarity was observed in any other organisms. The E.canG7_Brep sequence had 88.5% of identity and 70.2% of coverage in relation to terminal-repeat retrotransposon in miniature (TRIM) sequences of *E. granulosus* (G1) (pathogen_EgG_scaffold_0006_Trim-1250290-1250818) found by Koziol [37]. The second most frequent repetitive element was E.canG7_rep142 (254 loci) which had a high copy number in all of the flatworm species sequenced so far, being variable in all of the *Echinococcus* species (90–91.3% of identity), and included an open reading frame (ORF) encoding for pol polyprotein. Furthermore, E.canG7_rep142, E.canG7_rep39 and E.canG7_rep1032 contained sequences encoding for replicase domain, reverse transcriptase domain and RNA recognition domain, respectively. Recently, a novel TRIM has been found in *E. multilocularis* [37] but lacked evidence of active retrotransposition in *E. canadensis* (G7) and *E. granulosus* (G1). In addition, most of the E.canG7_rep (73%, 156/213) detected in *E. canadensis* (G7) had expression evidence (RNA-seq reads) in the protoscolex and metacestode stages. E.canG7_Brep and E.canG7_rep39 had a high number of RNA-seq reads (Additional file 2B).

### Comparative genomics among *Echinococcus* species

**Fig. 2:**
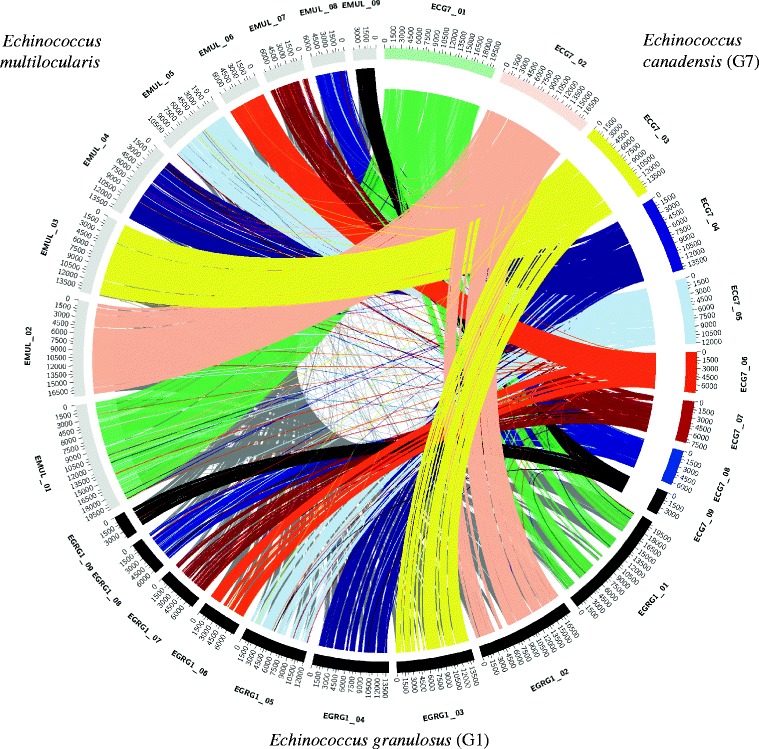
Circos Plot of the genome of *E. canadensis* (G7), *E. granulosus and E. multilocularis*. One-to-one orthologs connected according their distribution on their corresponding chromosomes

**Fig. 3:**
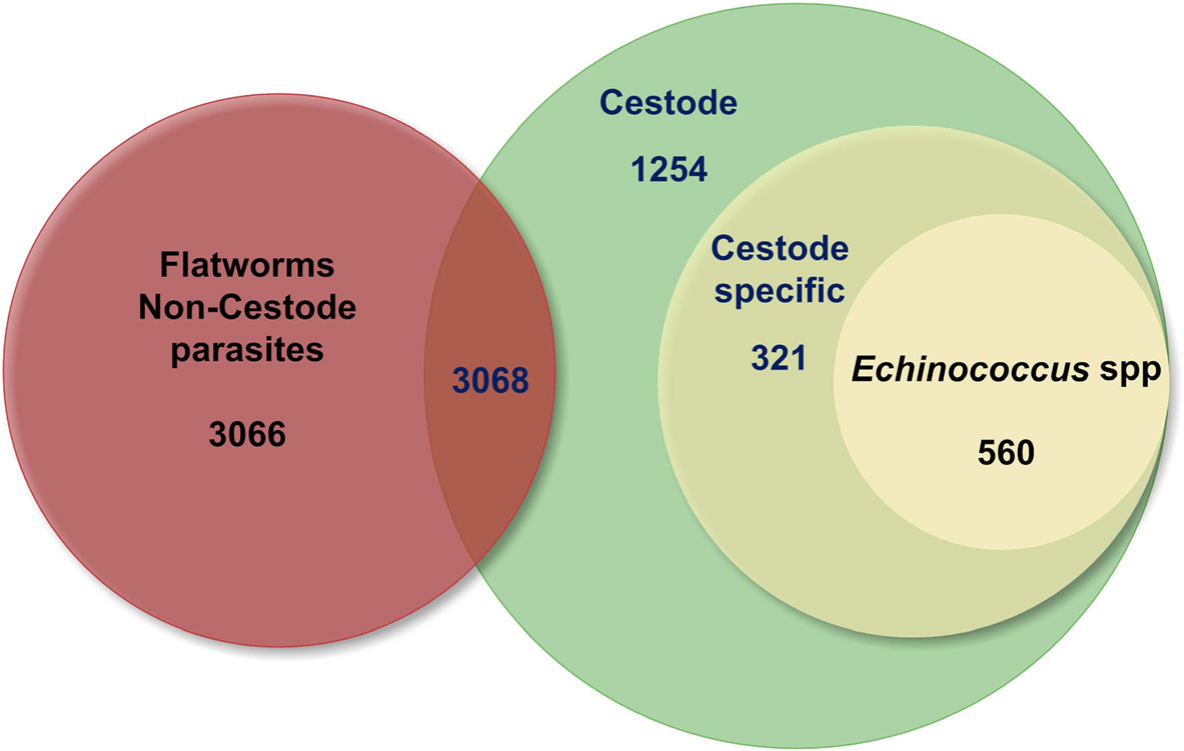
Orthologous genes present exclusively in *Echinococcus* species. Venn diagram illustrating the number of gene clusters in each analysed group

**Fig. 4:**
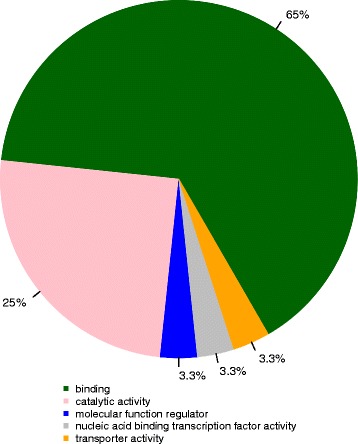
Proportion of *Echinococcus* species-specific proteins with functional information according to different Gene Ontology (GO) categories. Gene models from *E. canadensis* (G7) genome were classified according to molecular function GOterms

To determine the gene-order arrangement, we retrieved one-to-one orthologous gene pairs among *E. multilocularis*, *E. canadensis* (G7) and *E. granulosus* (G1) having previously ordered their contigs on *E. multilocularis* chromosomes and then using the OrthoMCL gene clustering (see next section). We found a high degree of conservation in the structure and organisation of genes within the three *Echinococcus* species (Fig. 2). The range of syntenic genes was from 89.1 to 97.1% (average 94.6%) between *E. canadensis* (G7) and *E. multilocularis* and from 95 to 99.7% (average 98.3%) between *E. canadensis* (G7) and *E. granulosus* (G1) (Additional file 1: 1.10). In order to perform orthology analysis, we constructed orthologs groups from a total of 14 proteomes of different organisms, including model organisms and representatives of the phylum Platyhelminthes. The total number of genes and orthology groups obtained in each organism are shown in Additional file 1: 1.11 and 1.12. From a total of 39,482 clusters of orthologous genes obtained, 6134 were present in all of the flatworm parasites and in at least one species of metazoan, among which 5203 orthologous groups were contained in all of the species of cestodes. The cestode category shared 3068 groups with at least one non-cestode species of flatworm parasites, 881 were cestode-exclusive and 560 groups were found only in *Echinococcus* species (Fig. 3). A total of 581 *E. canadensis* (G7) proteins belonging to *Echinococcus* specific orthologous groups were further analysed (Additional file 1: 1.13, 1.14 and 1.15), among which 80% were classified as hypothetical proteins. A total of 115 proteins had conserved domains and 49 proteins had molecular function GO term associated with them (Fig. 4). Two of the five GO categories grouped the 90% of all the *Echinococcus* specific proteins. These categories were binding (GO:0005488) and catalytic activity (GO:0003824).

### Expanded protein families in *Echinococcus*

A total of 26 expanded protein families consisting of 10 to 66 members were found in *Echinococcus* (Additional file 1: 1.16). Among them, we found the heat-shock protein 70 (Hsp70) family, which has been described by Tsai et al. [5] in all of the tapeworm genomes obtained so far [6]. We also found three interesting expanded families that were present only in the cestode orthology group: GPS motif-containing protein, Ubiquitin-conjugating enzyme and Glycosyl transferase. The *E. canadensis* (G7) GPS motif-containing protein is related to polycystin-1, a protein involved in central signal-transduction pathways and the GPS motif (PF01825) is the responsible of protein-protein interactions. Polycystins form an expanded family of proteins composed of multiple members in fish, invertebrates, mammals and humans. Ubiquitin-conjugating enzyme is known to be involved in the ubiquitination pathway, modulating proteins degradation and protein-protein interactions. The Ubiquitin-conjugating (UBC) complex consisted of up to 19 genes in *Echinococcus*. Protein sequence alignments showed a high conservation of the UBC superfamily domain (PF00179) only among cestode parasites. The third expanded proteins family is the glycosyl transferases family, which is involved in glycan biosynthesis and modifications. This important pathway could play an important role in the biogenesis of the acellular carbohydrate-rich laminated layer, which is a unique *Echinococcus*-specific trait and one of the morphological traits that differs among *Echinococcus* species. These protein families are composed of 10 members that are conserved in cestodes but are very divergent in relation to other organisms (Additional file 4).

### Drug targets

Protein drug targets previously described by us [6] were searched in the *E. canadensis* (G7) counterpart gene repertory. Comparative genomic analyses showed 89.4–100% of identity with *E. granulosus* and *E. multilocularis* proteins, whereas the range of identity with *S. mansoni* proteins was lower, from 34.9 to 97%. Phylogenetic analyses including reference proteomes showed that 20 out of 21 drug targets had human orthologs (Additional file 1: 1.17). In order to obtain more specific drug target genes we selected new candidates considering the following criteria: to be present in all of the cestode species, to have high sequence conservation among cestodes (MCL score <0.8) and to be absent or to have a high degree of divergence in humans (Table 2). A total of 42 cestode proteins were selected and all these putative drug targets were grouped into 7 categories: 1- antigens: Taeniidae antigen (AgB) and immunogenic protein Ts11; 2-defence: antimicrobial peptide; 3- signalling: neuropeptides and peptide hormones; 4- transport: vacuolar (H+) ATPase G subunit containing protein; 5- metabolic processes: dolichol phosphate mannosyl transferase subunit containing protein and EF-hand calcium-binding protein; 6- transcription processes: zinc finger C2H2-containing protein and 7- conserved hypothetical proteins. (Additional file 1: 1.18, 1.19, 1.20 and 1.21.) These proteins were subject of manual curation and are further described in Additional file 4.

**Table 2:** *E. canadensis* (G7) new drug targets proteins found in cestodes but absent or highly divergent in humans

Category	Product	*Ecanadensis* (G7) IDs
Antigens	Taeniidae antigen (Antigen B)	ECANG7_07838
	immunogenic protein ts11	ECANG7_01678
Defense	Antimicrobial peptide tachystatin A	ECANG7_00862
Sygnalling	neuropeptide-like protein	ECANG7_03703
	neuropeptide spp-like	ECANG7_10139
	Pancreatic hormone	ECANG7_09023
	Pancreatic hormone	ECANG7_05886
Transport	Vacuolar (H+) ATPase G subunit	ECANG7_02132
Metabolic process	Dolichol phosphate mannosyltransferase subunit 3	ECANG7_01023
	EF-hand domain containing protein	ECANG7_02884
Transcription processes	CREB binding protein	ECANG7_05946
	zinc finger, C2H2 type	ECANG7_07928

OG distant less than 0.8 and present in all cestodes species analysed (stricted criteria)

### Cytosine methylation in *Echinococcus*

Cytosine methylation is a conserved epigenetic feature that is found throughout the phylum Platyhelminthes. Metazoan DNA methyltransferases (DNMT1, DNMT2, DNMT3a/3b) are involved in catalysing this feature by transferring a methyl group (CH3) from S-adenosyl methionine (SAM) to the 5-carbon (C5) position of cytosine in the genomic DNA [38]. These “epigenetic marks” are subsequently recognized as binding CpG sites by methyl-CpG-binding domain proteins (MeCP2 and MBD1-4) and converted into signals that are necessary for generating phenotypic diversity [39]. DNMTs and MBDs complexes together with other proteins constitute the core of the metazoan DNA methylation system in both vertebrate and invertebrate species. Cytosine methylation has been observed in the *S. mansoni* genome and this epigenetic feature was directly dependent upon the presence of enzymatically active DNMT2 [40]. DNA methylation has been described in several Platyhelminthes species and homologous DNMT2 has been found in *S. mansoni* [41]. DNMT2 and cytosine methylation have been identified in the *E. multilocularis* protoscoleces. DNMT2 was also identified among the gene repertory of *E. canadensis* (G7), but neither DNMT1 nor DNMT3 orthologs were found (Additional file 5A). MBDs have been also observed in several species of Platyhelminthes [41], which seem to be members of the ancestral MBD2/3 family [42]. Despite sharing more than 70% of amino acid identity, which is indicative of a gene duplication event, mammalian MBD2 can bind methylated cytosine within genomic DNA, whereas MBD3 cannot. Therefore, further research is needed to identify whether these new platyhelminth MBD2/3 proteins are functionally closer to methyl-CpG-binding MBD2 or to non-methyl-CpG-binding MBD3. So far, only one MBD candidate has been found in each of the platyhelminth genomes. However, we not only found the previously described MDB protein in the *E. canadensis* (G7) genome (EcanG7_00768), but also identified a novel MBD that showed conserved domains and characteristic motifs of the MBD domain across members of the class cestode. The phylogenetic analysis grouped them into a specific cluster (Additional file 5B and C). These novel MBD proteins contain the N-terminal methyl-CpG-binding domain, which consist of 73 residues, a PHD domain (PF00628) of 48 amino acids in length and a bromodomain (PF00439) toward the C-terminal comprising 86 residues.

### CpG islands

**Fig. 5:**
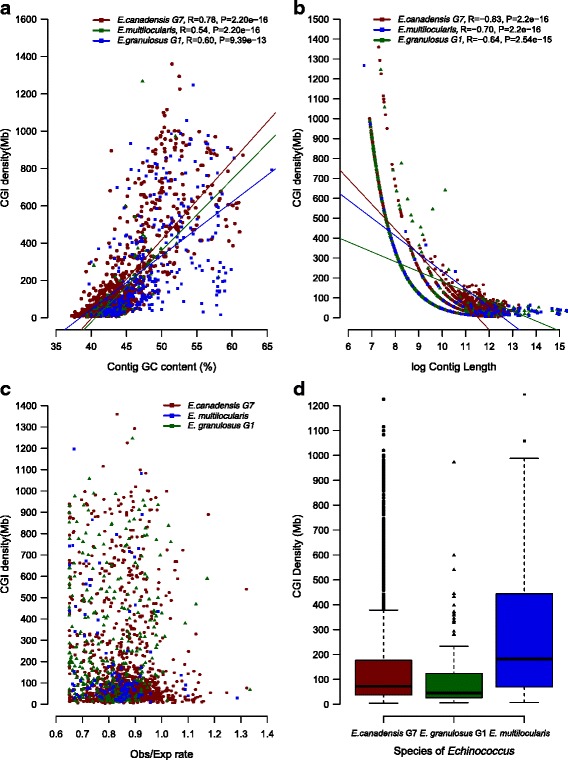
Correlations between CGI density and genomic features in the genomes of the three *Echinococcus* species. **a** CGI density (per Mb) versus contig GC content (%). **b** CGI density (per Mb) versus log (contig size). **c** CGI density (per Mb) versus contigs Obs.CpG/Exp.CpG. **d** CGI density (per Mb) by *Echinococcus* species

**Fig. 6:**
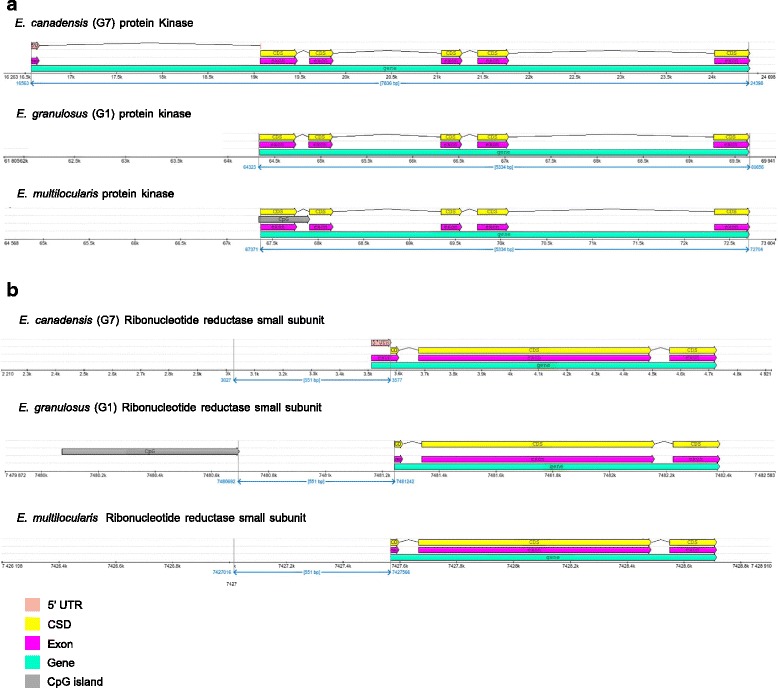
Genomic context of CpG islands associated with genes in *Echinococcus*. **a** ECANG7_03687 that codes for protein kinase . **b** ECANG7_06911 that codes for ribonucleotide reductase small subunit.

CpG is the pair of adjacent nucleotides that appear in a row on the same strand of DNA linked by a phosphodiester bond. CpG islands (CGIs) are palindromic stretches of DNA comprising about 1000 base pairs long that contain a higher CpG density than the rest of the genome and are not methylated [43]. CpG dinucleotides in GC-rich regions can be considered gene markers and may also play an important role in the regulation of gene expression. Approximately 70% of gene promoters reside within CGIs [44]. Enhancer elements can be located between CpGs or even include CpGs, thus hiding both the core promoter qualities and the enhancer qualities of the CGI [45]. Since many transcription factors binding sites are GC-rich, CGIs are likely to enhance binding to these sites, even in the absence of common promoter elements such as TATA boxes [46]. The methylation of CGIs results in stable silencing of gene expression [47]. It was also observed that, during gametogenesis and early embryonic development, CGIs undergo differential methylation [43]. In this work, CGIs distribution was evaluated in the three *Echinococcus* species and particular differences were detected among distinct genomic regions. We identified 4200, 4297 and 4249 CGIs, comprising a CGI density of 36.5, 37.4 and 36.9 (# of CGI per Mb) in *E. canadensis* (G7)*, E. multilocularis* and *E. granulosus* (G1), respectively. Compared to studies carried out on mammalian genomes [48], *Echinococcus* genomes exhibited similar length of the stretches of DNA where CGIs reside but higher levels of both, CGI density and observed/expected ratio of CpG. When we performed CGIs distribution analysis on coding regions and non-coding regions of *Echinococcus* genomes we observed that the number of CGIs and the CGIs density were much higher in the coding regions. We also evaluated the distribution of CGIs 5 kb upstream from coding regions and we identified 1363, 1637 and 1457 CGIs in *E. canadensis* (G7)*, E. multilocularis* and *E. granulosus* (G1), respectively. The CGI density was calculated considering only those regions where CGIs were found. The values of CGI density observed were 254.9, 253.1 and 241.7 respectively and slightly lower values than in coding regions but higher than in the rest of the genome were found (Additional file 6: 6.1). Furthermore, we estimated the correlation between CGIs density and other genomic features such as genome GC content, contig size and observed/expected ratio of CpG. In this regard, we observed a significant correlation between CGIs density and genome GC content, and between CGIs density and contig size in the three genomes of *Echinococcus* (Figs. 5a and b). By contrast, there was no correlation between CGIs density and the observed/expected ratio of CpG (Fig. 5c). We also estimated CGIs density per contig length only in those contigs containing CGIs for each *Echinococcus* species (Fig. 5d). The median of CGIs density in *E. canadensis* (G7) and *E. granulosus* (G1) genomes was lower in relation to *E. multilocularis* genome. The correlation between CGIs density and genome GC content differed among the three analysed regions, i.e. whole genome, coding regions and upstream coding regions. The same pattern was observed for the three *Echinococcus* species (Additional file 7). In *E. canadensis* (G7), the median of distance of CGIs from the start codon of genes was roughly 2 kb, and the range for most of the genes was between 500 and 3500 bp approximately. The same results were found in *E. granulosus* (G1) and *E. multilocularis* (Additional file 6: 6.2). We also identified CGIs differentially distributed along the genome of the three *Echinococcus* species, suggesting that the presence/absence of CGIs upstream *Echinococcus* genes could differentially regulate the gene expression. CGIs upstream *Echinococcus* genes are summarized in Additional file 6: 6.3, 6.4, 6.5, 6.6, 6.7 and 6.8. The numbers of genes differentially preceded by CGIs were 501 between *E. canadensis* (G7) and *E. granulosus* (G1), 548 between *E. canadensis* (G7) and *E. multilocularis* and 357 between *E. multilocularis* and *E. granulosus* (G1). Interestingly, we found drug target genes differentially preceded by CGIs. The genomic context for the ribonucleotide reductase small subunit and for the protein kinase that are differentially preceded by CGIs is shown in Fig. 6 for the three *Echinococcus* species.

### Small RNA pathway

Since the discovery of miRNAs in cestodes [49], many reports have highlighted the importance of miRNAs in several parasite species [50] because of their relevant roles in mammals where they control several pathways, such as developmental timing, haematopoiesis, organogenesis, apoptosis, cell proliferation and tumorigenesis. An important effector in miRNA pathways is the protein Argonaute (Ago). Phylogenetic analyses showed two clades for the Ago proteins: one clade grouped a highly conserved Ago protein found in all of the organisms. The other clade grouped new Ago/Piwi-like proteins present only in flatworm parasites and are very different from any other Ago/Piwi-like protein described in humans, mice and flies [6]. In order to further characterise Ago proteins in *Echinococcus* (G7) we identified the OrthoMCL clusters that included the Ago proteins and searched for the characteristic PAZ and PIWI domains. We found 4 Ago proteins with an amino acid identity that ranged between 69 and 99.7%. We confirmed the gene expression by RT-qPCR for the 4 Agos in *E. canadensis* (G7) metacestodes (Additional file 8A and B). In order to identify conserved domains and the possible interaction with a miRNA, 3D structures of Ago proteins were obtained by comparative modelling. For analysis purposes, we grouped the proteins into 4 groups (1 to 4), where the first one contained the conserved Ago proteins, which have high identity with the mammal Ago-2, and the three other groups contained the *Echinococcus* proteins of the new Ago clade (Additional file 8C). Each of the structures obtained showed RNAseH-like fold in concordance with the Ago/Piwi family proteins studied so far [51, 52] (Additional file 8D and E), except for proteins from group 3, which lack the N-terminal region and a stretch of the PAZ domain.

Ago proteins form a kind of channel or pocket in which the miRNA resides and interacts with residues of Piwi, Mid and PAZ domains and the target mRNA has access to mate with the miRNA [51]. The ArgoN domain located in N-terminal region contributes to the final conformation and has been described as being involved in the slicer activity. In spite of overall domain conservation, some particular residues are divergent between canonical and the platyhelminthic Ago/Piwi proteins, but they are conserved among proteins of this new clade (Additional file 8F and G). Moreover, the presence of motifs L1 and L2 adjacent to the PAZ domain, which consists of two beta-sheets and two alpha-helices, respectively, confirmed that proteins from *Echinococcus* Ago Group 1 belonged to the mammal conserved Ago family (Additional file 8H). In contrast to mammals, where other Agos lack the core related to slicer activity, we found the “DEDH” core in all of the *Echinococcus* Ago protein groups located in the Piwi domain which is decisive for the slicer activity of the protein on the target mRNA [52]. Furthermore, the presence of two defined motifs (Motif I and Motif II) at the N-terminal is also needed for the slicer activity (Additional file 8I and J). In humans, the methionine in Motif I plays an important role in the slicer activity of the messenger. On the other hand, Motif II comprises two alpha-helices on the exposed surface of the protein, which is involved in the activation of the RNA-induced silencing complex (RISC) and removal of the passenger strand [52]. In *Echinococcus*, the secondary structure of Motif I and II is conserved, but the expected methionine of the Motif I in the N-terminal region is not present in any Ago protein. The Ago proteins of the group 2 of *E. canadensis* (G7) and *E. granulosus* (G1) lack the domain ArgoN but present the ACT_6 domain instead. ACT domains bind to amino acids and regulate associated enzyme domains that could alter the interaction with RISC. Finally, we searched for residues involved in binding the seed region of the miRNA. The amino acid residues (QSKN) constitute a motif that bind to the miRNA seed region in the *Echinococcus* Ago Group 1 which is conserved in relation to human Ago-2, whereas the proteins motif of the new Ago- Piwi-like clade (Groups 2 to 4) have a different amino acids sequence. Surprisingly, this motif is conserved among the proteins of this new clade. The seed amino acid sequence of proteins in these groups is KDGT, except for the *E. multilocularis* EmuJ_000911600 protein that changes an aspartic acid (D) for a glutamic acid (E) (Additional file 8K).

### Whole genome *Echinococcus* single nucleotide polymorphisms

Next-generation sequence reads were mapped to a reference genome to identify single nucleotide polymorphisms/variations (SNPs/SNVs, hereafter referred to as SNPs). For all the analyses; the reads were first mapped against their own reference genomes and then against the genome of the corresponding analysed species. Homozygous and heterozygous variant sites were identified and were marked in both the reference and the alternative allele (see the Methods section for details). First, we evaluated the intraspecific variation in each *Echinococcus* genome. We observed the highest number of intraspecific variant sites in the *E. granulosus* (G1) genome, exhibiting a total of 74,585 SNPs which comprised a SNP rate of 0.64 SNPs each 1000 bp. The lowest number of intraspecific variant sites was observed in the *E. multilocularis* genome consisting of 1658 SNPs which exhibited a rate of 0.014 SNPs each 1000 bp. In regard to the *E. canadensis* (G7) genome, the number of intraspecific SNPs sites was 9449 resulting in a SNP rate of 0.09 SNPs each 1000 bp.

Genome-wide SNPs analyses performed among the three *Echinococcus* species revealed more SNPs between *E. canadensis* (G7) and *E. granulosus* (G1) than between *E. canadensis* (G7) and *E. multilocularis*. A total of 788,554 SNPs were identified between *E. canadensis* (G7) and *E. granulosus* (G1) genomes, comprising 1396 heterozygous and 777,710 homozygous SNPs. When we compared *E. canadensis* (G7) with *E. multilocularis* we found 327,802 SNPs sites comprising 215 heterozygous and 327,572 homozygous SNPs. In both cases the transition/transversion ratio was 2.97. Furthermore, the comparison of *E. granulosus* (G1) with *E. multilocularis* revealed 332,124 SNPs which included 6405 heterozygous and 325,691 homozygous SNPs and a transition/transversion ratio of 2.90 (Additional file 3: 3.4). The number of SNPs located in introns and intergenic regions was also higher between *E. canadensis* and *E. granulosus*. By contrast the number of SNPs in coding regions among the three *Echinococcus* species was fairly similar (Additional file 9A and B). Regarding the analysis of polymorphic sites in coding regions, we demonstrated that the percentage of synonymous substitution was higher than the percentage of non-synonymous substitution in all of the analysed species. Moreover, the rate of missense SNPs between *E. canadensis* (G7) and *E. granulosus* (G1) was slightly higher than between *E. canadensis* (G7) and *E. multilocularis* (Additional file 9C), which involved 7153 more non-synonimous SNPs. To confirm these results, we performed the same analysis as above using the *E. granulosus* (G1) genome obtained by Zhang et al. [5], which comprised a different parasite isolate, sequence technology and assembly methods than the employed by Tsai et al. [6]. The results obtained were virtually the same for both *E. granulosus* (G1) genomes. Finally, we assessed the SNPs distribution that causes changes in amino acid residues in 5668 *E. canadensis* (G7) proteins and its respective one-to-one ortholog in *E. granulosus* (G1) and in *E. multilocularis.* This analysis revealed a higher distribution of amino acid changes per each 100 residues of protein between *E. canadensis* (G7) and *E. granulosus* (G1) than between *E. granulosus* (G1) and *E. multilocularis,* and even higher than between *E. canadensis* (G7) and *E. multilocularis*. This result showed differences among the species that were statistically significant according to the Anova test (Additional file 9D). We also analysed the distribution of missense SNPs in genes of *Echinococcus* species associated to KEGGs pathways classified in 35 pathways. These results showed again a higher SNP density by gene between *E. canadensis* (G7) and *E. granuosus* (G1) than between *E. canadensis* (G7) and *E. multilocularis,* and between *E. granulosus* (G1) and *E. multilocularis* too. SNPs distribution among KEGGs pathways is shown in figure (Additional file 3: 3.5 and 3.6 and 9E).

### SNPs validation

In order to validate and verify polymorphisms detected by mapping NGS reads, we performed PCR amplification followed by direct sequencing of coding regions of some selected genes. This analysis was focused on validating missense SNPs; therefore the primers were designed based on exon sequences flanking at least one missense polymorphism. Since there are many SNPs among the different *Echinococcus* species, to select the best regions for primer design we detected exon regions with low variability. Selected regions were amplified by PCR of DNA extracted from 10 independent parasite isolates, three hydatid cyst of *E. canadensis* (G7), three hydatid cysts of *E. granulosus* (G1) and four metacestode isolates of *E. multilocularis*. Our results allowed us to characterise 48 polymorphisms (24 synonymous and 24 non-synonymous) as “high confidence” since all of the selected SNPs that were detected by NGS were also found by direct sequencing and in all of the analysed organisms (Additional file 3: 3.7).

### Phylogenetic studies of three *Echinococcus* species

The study of the *Echinococcus* phylogeny was addressed using 3 different approaches: mitochondrial genome, single copy genes and genome-wide SNPs analysis.

The mitochondrial genome assembly in addition to all the available *Echinococcus* mitochondrial genomes were used for the phylogeny reconstruction whereby it was possible to classify and discriminate among the *Echinococcus* species. The mitochondrial phylogeny obtained was according to Nakao [53] (Additional file 10A).

From the total of 39,482 clusters of orthologous genes we identified 29 single-copy groups which were subsequently used to perform a phylogenetic analysis. The phylogenetic trees obtained recovered the monophyly of Chordata, Arthropoda, Nematoda and Platyhelminthes in agreement with previously published results [6, 30] (Additional file 10B and C).

**Fig. 7:**
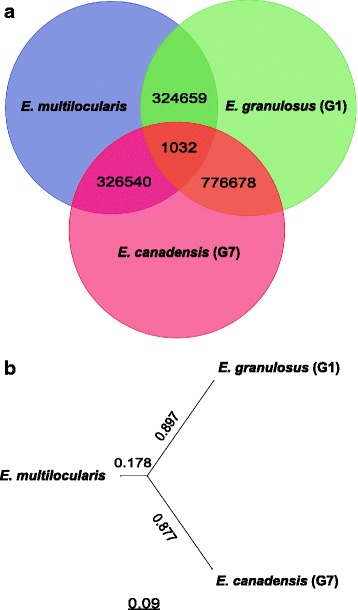
SNPs and Phylogeny based on genome-wide SNPs analysis **a** Homozygous SNP sites between *E. canadensis* (G7), *E. multilocularis* and *E. granulosus* (G1) species. The numbers in the overlap region indicate the number of SNPs between the species. The numbers in triple overlapping indicate the number of triallelic loci. **b** Phylogenetic tree based on genome-wide SNPs analysis by Maximum Likelihood method

Furthermore, to better understand the phylogenetic relationship between the taxa and to evaluate the contribution of SNPs to the genetic diversity among *Echinococcus* species, we selected only the homozygous SNPs and used them to perform a phylogenetic analysis. After removing heterozygous SNPs, the homozygous SNPs were concatenated and the resulting alignment was used to create a phylogenetic tree by implementing the Maximum Likelihood method. The tree topology demonstrated a higher genetic distance between *E. canadensis* (G7) and *E. granulosus* (G1) than between *E. canadensis* (G7) and *E. multilocularis,* comprising a total of 557,254 homozygous polymorphic sites. Moreover, *E. canadensis* (G7) and *E. granulosus* (G1) diverge from a common node close to *E. multilocularis* node (Fig. 7a and b). Since we observed that the number and type of changes varied according to the group of proteins analysed, random combinations of concatenated SNP sequences were also subjected to phylogenetic studies and all converged into the same topology. In addition, we registered how many polymorphic loci are shared among the *Echinococcus* species. For that purpose, we used a different reference genome in each one of the three rounds of mapping and we mapped the reads of the other two genomes against the selected reference. Using *E. canadensis* (G7) as reference, 32,142 SNPs were found at the same loci (29,756 had the same polymorphism). By contrast, using *E. multilocularis* as reference, we found 96,135 shared loci (94,990 loci with the same polymorphism). Finally, using *E. granulosus* (G1) as reference, 42,866 loci were found, (39,281 showed the same polymorphism) (Additional file 10D). Indeed, most of the SNPs were unique to one of the species and the highest number of shared loci was observed when the *E. multilocularis* genome was used as reference. It doubled and tripled the number of loci in relation to *E. canadensis* (G7) and *E. granulosus* (G1), respectively.

## Discussion

### The *Echinococcus canadensis* (G7) genome

*E. canadensis* (G7) is one of the most distributed parasites worldwide responsible for many human Echinococcosis cases. In recent years, several genomes of Platyhelminthes have become available; however it is yet an underexplored area. In this work we sequenced, assembled and annotated the 115-Mb genome of *E. canadensis* (G7) whose genome size is in the same order of magnitude as other Platyhelminthes including other members of the genus *Echinococcus*. The *E. canadensis* (G7) genome contains 11,449 genes with a core set of 881 orthologs shared among five cestode species. Our work demonstrated that the genome of *E. canadensis* (G7) is highly syntenic in relation to the other *Echinococcus* genomes, which makes comparative genomic analysis exceptionally interesting and crucial for addressing the study of flatworms.

Comparative genomics based on SNPs analyses results were unexpected. The intra specific SNP frequency of *E. canadensis* (G7) is almost 10 fold lower than the intra specific SNP frequency found in *E. granulosus* (G1). A high SNPs rate had already been observed for *E. granulosus* (G1) by Zheng et al. [5]; even though both genomes were sequenced from genomic material from a single cyst (originated from a single egg, which was assumed to be a clone). This observation indicates a lower intra cystic genetic diversity among protoscoleces of *E. canadensis* (G7) in comparison with protoscoleces of *E. granulosus* (G1). The higher genetic diversity between *E. canadensis* (G7) and *E. granulosus* (G1) was indeed unexpected due to they belong to the complex *E. granulosus sensu lato,* and therefore were supposed to be phylogenetically closer to each other than to *E. multilocularis.* On the other hand; despite the relevant number of SNPs among the *Echinococcus* species, a similar distribution of SNPs for all the KEGG pathways is observed for every gene, suggesting that there is no bias to accumulation of SNPs in a specific pathways.

### New drug targets

The analyses of protein domain sequences of new putative drug targets demonstrated that some *E. canadensis* (G7) proteins have greater differences with *E. granulosus* (G1) than with *E. multilocularis.* This information is relevant for developing a drug against these parasites and to be effective in geographic regions where different species coexist. As previously described by Cucher et al. [15], both species are the most frequent in human echinococcosis cases and are sympatric in many countries worldwide. At present, there are only two methods for the treatment of hydatid disease: surgery and the use of benzimidazole, but both have often unsatisfactory results [54]. Hence, novel treatment compounds are urgently needed. In this study, we identified several new potential drug targets against echinococcosis, which provided an expanded drug target repertoire. These proteins are highly conserved in cestodes but are absent or highly divergent in humans. One relevant feature would be related with the function of antimicrobial peptides. There is certain evidence that some proteins secreted by helminth parasites play a key role in modulating host immunity [55, 56]. Furthermore, studies of *Fasciola hepatica* have described a secreted peptide that exhibits similar biochemical and functional characteristics to the human cathelicidin LL-37 and modulates the innate cell response by mimicking the function of host antimicrobial peptides [57, 58]. Analysis of peptide structure in addition to orthology studies and its conservation in cestodes suggest that the *E. canadensis* (G7) possesses peptides that may exert an antimicrobial activity, as the proposed for the leucine-rich-repeat-(LRR)-containing proteins and the lectin-like proteins identified by Zheng et al. [5], or may play an immunomodulatory role as the proposed for the cathelicidin-like peptide of *F. hepatica*. Highly expressed secreted peptides at infectious stages of the life cycle could be involved in host-parasite cross-talk and could play an essential role in regulating host immune responses which would prolong the parasites survival in mammalian host species. Another relevant feature is related to *E. canadensis* (G7) neuropeptides that could be useful for performing biochemical studies of signal transduction, which have been hindered due to the inability to obtain large quantities of flatworm neuronal tissues. Indeed, these genes may represent potential drug targets for developing new therapeutic interventions or novel biopharmaceutical components.

### Regulation of gene expression

A relevant genomic feature is related to the CGIs distribution that was studied in *Echinococcus* genomes for first time in this work. Since CGIs are considered gene markers, they are expected to have a high correlation with the gene density which indeed was found in the *Echinococcus* genome structure. Both the number of extant CGIs and the CGI density were much higher than in non-coding regions, which is similar to what was observed in mammalian genomes. Our studies also demonstrated that the CGI density is in correlation with the distance from the start codon of genes and the corresponding upstream region. The position CGIs and its corresponding CGIs density in upstream coding regions may help to identify promoter regions by experimental and in silico approaches; tasks that have not yet been addressed in Platyhelminthes. The diversity of eukaryotic promoters is the main obstacle for their characterisation, which is very important to understand the molecular mechanisms of gene transcription and would provide valuable information for the genetic manipulation of these parasites. *Echinococcus* genomes exhibited a higher CGIs density than the mammalian genomes, which is in accordance with the low levels of DNA methylation reported in some Platyhelminthes genomes and similar to many non-mammalian model organisms, such as round worms and flies, which also lack or have low levels of methylated DNA [59]. Experimental and computational studies corroborated that CGIs tend to vanish during genome evolution by a mechanism of de novo methylation of their CpG dinucleotides, which subsequently change to TpGs or CpAs due to a very high methylation-dependent transition rate [60–64]. The mammalian proteome contains many members of DNMTs and MBDs proteins, which is in accordance with the significantly lower CGIs density rate in comparison with the genomes of *Echinococcus* species. The high CGIs density rate ~ 37 CGIs/Mb in the *Echinococcus* genomes is well explained by the absence of other members of the family of DNMTs and MBDs proteins. This phenomenon is in agreement with the low levels of methylation observed in other members of the Platyhelminthes phylum, such as *E. multilocularis*, the monogenean *Protopolystoma xenopodi* and the turbellarian *Polycelis nigra* and *Macrostomum lignano* species [41, 65]. *E. canadensis* (G7) also provides an interesting model for studying fine-tune gene regulation during parasite development, because protoscoleces can rapidly respond to environment signals, giving rise to hydatid cyst or adult worms. On the other hand, since the *E. canadensis* (G7) genome has a small number of members of the DNA methylation system, as well as a novel and genus-specific MBD protein family member, this organism will provide an important parasite model for studying the evolution of methylation in Platyhelminthes.

Another regulatory mechanism is related to the differential expression of small RNAs during parasite development in *E. canadensis* (G7) [31]. We performed structural analyses of Ago proteins to gain further insight into small RNA pathways. Computational analyses including phylogenetic and protein structure studies confirm a new protein Ago clade in *Echinococcus* and suggest a possible interaction with miRNA. The conservation of some particular residues could play an important role in binding small RNA, in the stabilization of the mRNA target and in the slicer activity. On the other hand, specific amino acid changes in the motif that binds the seed miRNA site were also identified and are conserved through the new Ago clade. These particularities may result into a distinctive small RNA specificity and therefore a differential genes regulation that could be evaluated in future experiments. Furthermore, since *Echinococcus* lacks classical Piwi proteins [6] and also seems to lack piRNA molecules [31] it would be interesting to perform studies to identify molecules that interact with this new Ago clade.

### Expanded protein families

We have previously identified an expansion of the Hsp70 gene family in tapeworms [6]. In this work, we identified three cestode-specific expanded gene families that could play important roles in the parasites biology: the ubiquitin-conjugating enzyme, GPCR-proteolytic site (GPS) motif-containing protein and Glycosyl transferase. The *E. canadensis* (G7) GPS motif-containing protein and the ubiquitin-conjugating enzyme could be involved in important pathways, such as cell signalling and protein-protein interaction. *Echinococcus* species exhibit an unusual high degree of developmental plasticity and gene expansions that could be explained by these phenomena. Glycosyl transferases are involved in glycan biosynthesis and modifications. These important pathways are involved in the biogenesis of the acellular carbohydrate-rich laminated layer, which is an *Echinococcus*-specific trait and one of the morphological traits that differentiate *Echinococcus* species. This expanded gene family presents sequences that are different from known glycosyl transferases. It could be interesting to determine whether the non-canonical enzymes have a metabolic role in metacestode development of each *Echinococcus* species.

### Phylogenetic relationship among *Echinococcus* species

At present, there is not unequivocal taxonomic classification of *Echinococcus* species neither genomic analysis that reveals particularities among them. The phylogeny studies based on three different approaches allowed us to understand the phylogenetic relationship among these three species. Firstly, the reconstruction of the mitochondrial phylogeny from complete mitochondrial genomes confirms the monophyly of the *E. canadensis* species. Moreover; the phylogenetic tree topology of single-copy genes based on amino acid or nucleotide sequences demonstrates the status of *E. canadensis* (G7), *E. multilocularis* and *E. granulosus* (G1) as different species. However, slightly different topologies are observed when protein or nucleotide sequences are used for the phylogeny reconstruction, which make this analysis robust enough to discriminate among species but it is not sufficient to define a common ancestor. Furthermore, considering only coding regions, the analyses could be biased due to sampling bias and/or due to the small divergence rate among proteins, therefore the real genetic distance could be underestimated. Otherwise, whole-genome sequencing and a SNP-based approach provided the requisite level of genetic detail to resolve the paradigm of these *Echinococcus* species. This analysis demonstrates that *E. canadensis* (G7) and *E. granulosus* (G1) are phylogenetically more distant to each other due to a higher rate of genetic diversity and suggests that *E. multilocularis* would be the ancestral species of both. This result is in accordance with the phylogeny reconstructed from single-copy genes based on nucleotide sequences here obtained and with previous studies carried out by Saarma et al. [66]. The fact that *E. canadensis* (G7) and *E. granulosus* (G1) share more homozygous polymorphic loci with the same variant reinforces the hypothesis of a basal *E. multilocularis* that accumulated mutations over time until a speciation phenomenon occurred. Afterwards they would have diverged independently by increasing the genetic diversity. Indeed, whole genome sequencing is crucial not only for exposing differences among the species, but also for unequivocally defining the phylogeny and the evolutionary history of these parasites and other species. Hence, additional cestode genomes need to be sequenced in order to understand the complete evolutionary history and to obtain an accurate *Echinococcus* phylogeny.

## Conclusions

In the current study we are presenting a new genome of *Echinococcus*. We sequenced, assembled and annotated the genome of the human flatworm parasite *E. canadensis* (G7) which highly contributes to the source of knowledge of the flatworm biology. The present work focused on the description of a group of genes that are involved in parasite development and survival, metabolic features and relevant genomic structures involved in gene expression regulation. And finally; we performed a thorough genetic variability analysis among the *Echinococcus* species that was taken in advantage to perform phylogenetic analyses. These results lay the groundwork for further research of *Echinococcus* phylogeny and many others aspects of the evolutionary history of these parasites. The resources given in this work not only promote the study of parasite developmental mechanisms, but will also provide new tools for drug discovery and control strategies.

## Methods

### Data availability

The assembled sequences of the *E. canadensis* (G7) genome were deposited in ENA (BioProject PRJEB8992, https://www.ebi.ac.uk/ena/data/view/PRJEB8992) and Wormbase Parasite (http://parasite.wormbase.org/Echinococcus_canadensis_prjeb8992/Info/Index/). Data on orthology groups can be downloaded from the web page of FlatDB project (http://www.bmhid.org.ar/flatdb/).

### Sample collection, DNA extraction and next-generation sequencing

#### Parasites material

All *E. canadensis* (G7) materials were collected from Buenos Aires, Argentina. Fertile hydatid cysts were obtained from the livers of naturally infected pigs provided by abattoirs from Buenos Aires, Argentina. The animals involved in this study were not subjected to any experimental procedure. All of the samples used in the study were collected post-mortem in commercial abattoirs. For genome sequencing purposes, we collected a large unilocular cyst from a pig’s liver and the hydatid fluid was aseptically aspirated from cysts with a syringe. Protoscoleces (PS) were recovered from aspirated fluid and extensively washed in Phosphate Buffer Saline (PBS) to remove dead PS and cyst wall debris. One fraction of freshly isolated PS from each cyst was used for determining viability by eosine exclusion test. Samples showing more than 90% of viability were selected for DNA isolation. The species and genotype were determined by sequencing a fragment of the mitochondrial cytochrome c oxidase subunit 1 (CO1) [49, 67]. The resulting species and genotype was *E. canadensis* (G7).

### DNA isolation, library construction and sequencing

PS were treated with pepsin in order to eliminate the remaining host tissue, and proteinase K was used to break cell wall and release the genetic material. Isolation of high-quality genomic DNA was performed by phenol/chloroform method. Samples were quantified using a Qubit Fluorometer (Invitrogen) and quality was evaluated by rate OD 260/280 and OD 260/230 using a NanoDrop (ThermoFisher Scientific). Library preparation and miSEQ Illumina sequencing were performed at the Genomics and Computational Biology Group at Centro de Pesquisa René Rachou (CPqRR), Oswaldo Cruz Foundation, Minas Gerais, Brazil. For each library preparation, 50 ng of DNA were subjected to reaction of random tagmentation, and DNA was simultaneously fragmented and linked to specific adapters using Nextera® XT DNA Sample Preparation Kit, according to the manufacturer’s instructions. Then, genomic DNA was purified and subjected to an amplification reaction using primers complementary to the adapters. The products were quantified by qPCR using KAPA™ SYBR® FAST qPCR Kit. Two libraries of 1050 pb and 1200 pb fragment size were prepared. Libraries were diluted in Tris–HCl solution + Tween 0.1%, deposited in a flowchip and subjected to 500 and 600 sequencing cycles (2× 250 bp and 2×300, respectively) using MiSeq v2 Reagent Kit. The images were processed and analysed with the manufacturer-supplied software. The quality of the Illumina reads was evaluated with FastQC v0.10.1, and the reads were trimmed and end-clipped to a phred score of 33 using Trimmomatic [68].

### RNA isolation and RT-PCR

The metacestodes were kept in liquid nitrogen until they were used. The samples were centrifuged and the pellet was immediately carried at −85 °C in TRIzol (ThermoFisher Scientific) reagent in suitable proportion. Subsequently, chloroform was added and the procedure was conducted as described by the manufacturer. Isolated RNA was treated with RQ1 DNase - Free RNase (Promega) following the manufacturer’s protocol. cDNA was obtained using SuperScript III First-Strand Synthesis System for RT-PCR (Invitrogen, Life Technologies). Long PCR kit Enzyme Mix (Fermentas ThermoFisher Scientific) with proofreading activity was used for the amplification of cDNA of high molecular weight. The cDNA obtained was sequenced as previously described. In order to determine the degree of contamination with host RNA, RT-PCR was performed using pig actin primers. The selection of reagents, temperatures and cycling time, primers and performing calibration curves for each Argonaute genes is shown in the Appendix. Finally, the selection of these variables was as follows: Real Time PCR Reagents brand Firepol Eva Green (Solis BioDyne) and primers purified by HPLC. RT-PCR reaction was performed in a thermal cycler Rotor Gene 6000 5 Plex (Corbett, Qiagen). Primers were designed for amplification of full messengers and RT-qPCR (Additional file 8L). Primers were also tested not to be paired with pig genes.

### PCR amplification and sequencing

The amplification reaction for genotype verification was performed as described by Kamenetzky et al. [20]. Fragments for SNPs in vitro verification were selected by containing at least one SNP, thereby causing a missense mutation. Since there are many SNPs among the different *Echinococcus* species, to select the best regions for primer design, we detected exon regions with low variability. The PCR reaction was performed into a final 50 ul volume containing sample DNA (5–20 ng), 200 uM of each dNTP (Invitrogen), 1.5 mM MgCl_2_, 0.2 uM of forward and reverse primers, and 2U of DNA taq polymerase (Additional file 3: 3.8). Thermocycling conditions started with denaturing at 95 °C for 5 min, thermal cycling was performed for 35 cycles at 95 °C for 45 s, followed by 50 s at 57 °C and then by 72 °C for 60 s. Reactions were finished by 5-min incubation at 72 °C. Amplification products were checked in 1.5% agarose gels stained with gelRed to verify the presence of a single amplification product. Sequencing was performed in an Applied Biosystems 3130 capillary sequencer using a Big-Dye terminator cycle sequencing kit, according to the manufacturer’s instructions. Gene fragments were PCR-amplified from 3 representative isolates of *E. canadensis* (G7), 3 of *E. granulosus* (G1) and 4 of *E. multilocularis*, and they were sequenced in both strands. Base calling of chromatograms, assembly of sequences, detection of polymorphisms and manual inspection of assembled sequences and polymorphisms was done using a software package composed of Phred - (version phred-dist-071220.c), Phrap (version 1.090518) and Consed (version 0.29). Basecalling of chromatograms was done by Phred. Sequences were then assembled by Phrap-Consed. All candidate SNPs were identified and subjected to manual inspection.

### De novo assembly of *E. canadensis* (G7) NGS reads and removal of non-target sequences

The *E. canadensis* (G7) genome sequence was assembled from a combination of the two libraries sequenced using the Illumina MiSeq platform. Sequence data were screened against host genome. In order to optimally assemble the genome of *E. canadensis* (G7), we tested different de novo assemblers and measured statistical parameters to define the best assembly. Velvet1.2.07 [69], SOAPdenovo2 [70] and SPAdes 3.6 [27, 71] were used on the Illumina reads. Reads and obtained contigs were reused to generate the high-quality genomes using PAGIT IMAGE [72]; ICORN [73]; ABACAS [74]. This initial assembly process resulted in the assembled genome version 1. Metrics of the quality of the assemblies obtained by DBG approaches were evaluated by QUAST [75] based on standard assembly metrics, such as N50, total number of contigs and total length of the assembly. In addition, the conjunction of all the information was employed to select the best assembly (Additional file 1: 1.1). Putative non-target contigs and contigs that were shorter than 1 kb in length were removed before performing further analyses. The completeness of the gene space was validated using CEGMA 2.4 [28]. Genome-wide quality comparison of *Echinococcus* species was performed by mapping contigs against the *E. multilocularis* genome using ABACAS.1.3.2 [74]. Coverage and depth coverage were calculated with custom scripts. Depth coverage refers to the number of times that the same region or position in the reference genome is represented by the assembled genome. Coverage refers to the percentage of the total length of the reference genome that is represented by the assembled genome.

### Gene prediction and annotation

*E. canadensis* (G7) specific repeat families, including simple repeats, interspersed repeats and satellite DNAs, were identified from the assembled genome using RepeatModeler [33]. Subsequently, the automated annotation pipeline MAKER 2.31 [29] was used for ab initio gene finding. For gene model predictions, a reliable group of genes and proteins of related species of the genus *Echinococcus* was selected from different databases in order to generate a reliable training dataset for Augustus [76] and SNAP [77]. Protein data were also incorporated through exonerate version 2.2.0 [78]. Moreover, RNA-seq data generated by the Wellcome Trust Sanger Institute was incorporated. RNA-Seq reads were mapped to the *E. canadensis* (G7) genome with Tophat v2.0.12 [79, 80] and the output was used to construct a Hidden Markov Model for GeneMark [81] through the automatic training of the eukaryotic ab initio gene-finding algorithm GeneMark-ET [82]. The automated annotation pipeline MAKER 2.31 [29] was used for structural annotation as follows: repetitive DNA was masked using RepeatMasker [83] and the *E. canadensis* (G7) specific repeat library initially created using Repeat Modeler [33, 83]. Gene models identified by CEGMA 2.3 [28] were passed through MAKER 2.31 [29]. Three rounds of MAKER2 were performed. A first pass of MAKER2 was run using a custom protein database as physical evidence and employing GeneMark [81], SNAP [77] and Augustus [76] for ab initio predictions. Subsequently, a second pass of MAKER2 was performed using physical EST evidence from RNA-Seq from GFF3 file obtained with GeneMark-ET [82] and with all of the ab initio predictors turned off. The final pass of MAKER2 was run using physical EST evidence from RNA-Seq from GFF3 file obtained with GeneMark-ET and with all of the gene predictors turned on, plus gene models obtained in round 2 passed through MAKER2 as EST with a threshold AED score of 0.5. Thus MAKER2 then reconciled homology-based physical evidence with the results of purely ab initio predicted gene models. Transfer RNAs were searched by tRNAscan-SE 1.23 [84], each tRNA locus was confirmed by RNA central database (http://rnacentral.org/) analysis. Two adjacent tRNA genes were defined as clustered if their distance was less than 1000 nucleotides, according to Bermudez et al. [32]. Ribosomal RNAs were predicted by RNAmmer-1.2 software (www.cbs.dtu.dk/services/RNAmmer/) and BLAST searches against NCBI and 5S rRNA (http://combio.pl/rrna/) databases. Specific miRNAs of *Echinococcus* were identified as previously described [31]. BLAST [85] was used to map miRNAs against the genome The data were added to final coordinates GFF file. Custom scripts were used to achieve the correct format files.

### Repetitive elements annotation

Repetitive elements of *E. canadensis* (G7) were automatically identified using RepeatModeler v1.0.8. In addition, a reliable set of repetitive elements described for flatworms was used in homology searching against the genome. Custom scripts were used to select the best match from overlapping matches in RepeatModeler and homology searches. For comparative genomic analyses, we selected sequences with more than 50% of coverage of consensus repetitive sequences and classified them with TEclass software (a tool for automated classification of unknown eukaryotic transposable elements [86]). Sequences from RepeatModeler and from reported *Echinococcus* species entries were remapped on the *E. canadensis* (G7) genome. Only hits with e-values lower than 0.0001 and with more than 50% of coverage were counted as genome matches, and the genome localization was obtained with exonerate version 2.2.0 [78].

### Gene model analyses and gene annotation

The performance of gene annotation and basic statistics for *E. canadensis* (G7) gene models, including average intron/exon lengths and number of introns were calculated using Eval [87]. Gene annotation was performed by a combination of three methods. All gene models were screened for known domains using InterProScan-5.7-48.0 [88] and InterPro2GO databases were used to assign Gene Ontology terms [89]. Gene models were subjected to a BLAST search [85] against UniprotDB and the Parasites orthology group built with OrhtoMCL v2.0.9 [90, 91] was used to define the final annotations.

### Orthologous gene groups

The protein complements of 14 metazoan taxa (Additional file 1: 1.11) were searched for reciprocal best hits using BLAST [85], and the results were subsequently employed to identify orthologous gene clusters by using the MCL algorithm [91]. An inflation value was set in 1.5 and a subset of proteins was clustered according to taxa and other criteria summarized in Additional file 1: 1.12. To detect expanded protein families, orthology groups built with OrthoMCL were used. Clusters containing more than 3 proteins per species were identified in all of the protozoa species and in *Echinococcus* phyla.

### Drug targets

In order to identify cestode-specific genes that are absent in humans, we selected orthologous genes of *E. canadensis* (G7) from orthology groups. A stringent inflation value of 0.8 was used to perform clustering of proteins that had already been reported as drug targets. In order to obtain more specific drug target genes we selected new candidates considering the following criteria: to be present in all of the cestode species, to have high sequence conservation among cestodes (MCL score <0.8) and to be absent or to have a high degree of divergence in humans (Table 2). Phylogenetic analyses were performed for all of the orthology groups selected by ClustalX multiple alignments and maximum likelihood algorithm was implemented in MEGA 5 software.

### Domain annotation and structural analysis

Proteins studied in this work were searched using BLAST [85] against UniProtKB/Swiss-Prot databases. Protein domains were screened against PFAM, and Prosite databases using PFAM_scan ([92] or HMMscan 3.0. Signal peptide analyses were performed using Phobius (http://www.ebi.ac.uk/Tools/pfa/phobius/) and SignalP 4.0 (http://www.cbs.dtu.dk/services/SignalP/). Secondary structure analysis was assessed with PSIPRED (http://bioinf.cs.ucl.ac.uk/psipred/) and Jpred 4 (http://www.compbio.dundee.ac.uk/jpred/). Protein structure modelling was obtained with PHYRE2 [93] and SWISS-MODEL [94–97]. PDB databases were used for homology searching and Human Argonaute-2 - miR-20a complex (http://www.rcsb.org/pdb, [51]) was used for structural comparison analyses.

### CpG islands

In order to identify CpG island, CgiHunterLight software tool v1.0 (http://cgihunter.bioinf.mpi-inf.mpg.de/) was applied, and Takai and Jones algorithm [98] was used, which suggests an optimal set of parameters (GC content ≥55%, Obs CpG/Exp CpG ≥0.65 and length ≥500 bp). This algorithm can effectively exclude false positive CGIs from repeats. We evaluated CpG islands in different genomic regions of the three *Echinococcus* species, genomic, coding and upstream coding regions using custom scripts.

### Genome comparison

Genome-wide quality comparison of *Echinococcus* species was performed by mapping contigs against the *E. multilocularis* genome using ABACAS.1.3.2 [74], and coverage and depth coverage were calculated with custom scripts. The results of orthology analyses were used to define gene orthology regions and synteny in the chromosome context. Synteny of orthologous genes was represented by CIRCOS plot [99].

### SNPs

All of the Illumina reads from *E. canadensis* (G7), *E. granulosus* (G1) and *E. multilocularis* libraries were filtered by quality and then mapped onto the *Echinococcus* species genomes using bowtie2 [100]. Mapping statistics were calculated with Bamtools (https://github.com/pezmaster31/bamtools), and duplications were marked and discarded using picard-tools v-1.129 (http://broadinstitute.github.io/picard/). SNPs were called using Samtools [101], with the following parameters: variation frequency was set >40% with at least 20 reads covering SNP sites, and the base quality of both reference site and variation site was >30. Insertion and deletions (indels) were filtered out with VCFtools [102], and SNPs with less than 10 pb from indels were removed to avoid false positives in SNP calling. For all SNPs analyses the reads were first mapped against their own reference genomes and then against the genome of the corresponding analysed species. Heterozygous sites were only retained if both forward and reverse reads mapped against the reference and alternative allele at a given nucleotide position with more than 20 reads supporting that position. Homozygous polymorphic sites were annotated if the forward and reverse reads mapped onto the alternative allele with more than 20 reads supporting that position and if there were no reads supporting the reference allele. Homozygous and heterozygous SNPs sites were registered for all of the species. Transition/transversion ratios were calculated using VCFtools [102], and the annotation and classification of SNPs based on the effect on coding regions were carried out with SnpEff v4.0 [103]. Comparison among three of *Echinococcus* species was performed with custom scripts. Statistical analyses were performed by ANOVA test, with a confidence level of 95% using R software and package car. Graphics were built using R software (https://www.r-project.org/).

### KEGGs pathways

Metabolic pathways in the whole proteome were obtained from the KEGG database using the KAAS tool [104]. In order to provide an overview of secondary metabolite biosynthesis and a hand-picked selection of important regulatory pathways and other functional modules, we used KEGG Mapper Reconstruct Pathway for the visualization and analysis of cellular pathways.

### Phylogeny reconstruction with mitochondrial genomes

Mitochondrial contigs were identified by BLAST [85] against a customized mitochondrial database of *Echinococcus* species. Contigs were reassembled into complete mitochondrial genome and gene annotation was performed using RATT [105]. Nucleotide sequences of 13 protein-coding genes from mitochondrial *Echinococcus* genomes were translated to amino acid sequences using the flatworm mitochondrial genetic code [106]. The deduced amino acid sequences were aligned by ClustalX multiple sequence aligner [107]. Phylogeny reconstruction with mitochondrial genomes was implemented using the Maximum Likelihood method based on the JTT matrix-based model performed with MEGA5 software. The bootstrap consensus tree inferred from 100 replicates was taken to represent the evolutionary history of the taxa analysed. Branches corresponding to partitions reproduced in less than 50% bootstrap were collapsed. The analysis involved 13 mitochondrial genomes with 12 proteins each (3276 amino acid sites positions). The values of each node are ML bootstrap percentages.

### Global phylogeny of *Echinococcus* and model species

Single-copy ortholog groups were selected and protein sequences were concatenated by organism species. Protein sequences were aligned using ClustalX multiple sequence aligner. Global phylogeny of *Echinococcus* and model species were analysed using the Maximum Likelihood method based on the JTT matrix-based model conducted in MEGA5. All of the positions with less than 60% site coverage were eliminated. The analysis involved 14 amino acid sequences from 29 single-copy genes from orthology groups. There were a total of 7001 positions in the final dataset. Nucleotide sequences from single-copy genes were aligned with transAlign software (EMBOSS), and phylogenetic analysis was performed by Maximum Likelihood method based on the Tamura-Nei model. All of the positions containing gaps and missing data were eliminated. There were a total of 14,364 positions in the final dataset. Genome-wide SNPs were used to perform phylogeny analysis as follows: only the homozygous SNPs were selected to correct for complete lineage sorting. After removing heterozygous SNPs, the homozygous SNPs loci were concatenated and the resulting alignment was used to create a phylogenetic tree by implementing the Maximum Likelihood method.

## Additional files

Additional file 1: Table of metrics and annotations. 1.1 Metrics summary table for assemblies obtained by different assemblers for the genomic *E. canadensis* (G7) NGS reads. Only contigs > 1000 bp are considered. DBG-de Bruijn graph; PDBG- Paired de Bruijn graphs. 1.2 Mitochondrial genome. 1.3 *E. canadensis* (G7) gene identifier and annotation, 1.4 Interpro and GO annotation; 1.5 tRNA annotation, 1.6 rRNA annotation; 1.7 miRNA annotation; 1.8 Repetitive DNA 1.9 Repetitive Genome coordinates. 1.10 Percentage of syntenic genes among *Echinoccocus* species; 1.11 Metazoan taxa used for OrthoMCL analysis; 1.12 O*rthologous gene groups 1.13 Cestode orthology groups; 1.14 Echinococcus specific orthology groups 1.15 E. canadensis (G7) proteins ID from Echinococcus specific orthology group. 1.16 Expanded family proteins in the genus Echinococcus. 1.17* Previously described drug targets in *Echinococcus;* 1.18 V-ATPase; 1.19 Peptide hormone; 1.20 Dolichol- phosphate mannosyl transferase (DPM) enzyme;1.21 Zinc finger domain containing protein. (XLS 5476 kb)
Additional file 2:
*Echinococcus canadensis* (G7) functional annotation and highest repetitive elements read counts. (A) Molecular function GO terms associated frequency of *E. canadensis* (G7). (B) E.canG7_Brep and (C) E.canG7_rep39 per-base coverage with reads from the forward strand (blue) and reads from the reverse strand (red) are shown. (PDF 295 kb)
Additional file 3: Table of metabolic pathways and associated SNPs. 3.1 *E. canadensis* (G7) KO; 3.2 *E. canadensis* pathways; 3.3 Complete pathways modules; 3.4 Number of Homozygous and heterozygous variant sites of *E. canadensis* (G7), *E. multilocularis* and *E. granulosus* (G1).3.5 Gene distribution in KEGG pathways; 3.6 SNPs density in KEGG pathways. 3.7 List of genes subject of experimental validation in SNPs analyses; 3.8 Table of primers for SNP validation. (XLSX 699 kb)
Additional file 4: Expanded proteins families and drug targets: Multiple alignment of expanded proteins families and drug target sequences of *E. canadensis* (G7) and their orthologs. (PDF 999 kb)
Additional file 5: DNMT and MBD proteins of *Echinococcus*. (A) Phylogenetic tree of *Echinococcus* DNMT proteins and classification in family class. (B) Phylogenetic tree of MBD proteins. New MBD protein and families classification. (C) Amino acidic residues involved in MBD motif are shown as logo plots. (PDF 295 kb)
Additional file 6: Table of CGIs. 6.1 CpG islands and other genomic features in *Echinococcus* species genomes. 6.2 Summary of correlations between CGI density and genome features in the three *Echinococcus* species; 6.3-6.8 Differential CGIs distribution upstream *Echinococcus* genes (presence/absent in *Echinococcus* genomes). (XLS 204 kb)
Additional file 7: Correlations between CGI density and coding regions in the genomes of the three *Echinococcus* species: (A) CGI density (per Mb) versus contig GC content (%). (B) CGI density (per Mb) versus log (contig size). (C) CGI density (per Mb) versus contigs Obs.CpG/Exp.CpG. (D) CGI density (per Mb) by *Echinococcus* species. Correlations between CGI density and upstream coding regions in the genomes of the three *Echinococcus* species: (E) CGI density (per Mb) versus contig GC content (%). (F) CGI density (per Mb) versus log (contig size). (G) CGI density (per Mb) versus contigs Obs.CpG/Exp.CpG. (H) CGI density (per Mb) by *Echinococcus* species. (I) Boxplots of average CGIs distance from the start codon of genes in the three *Echinococcus* species. (PDF 257 kb)
Additional file 8: Argonaute proteins in *Echinococcus*. (A) Relative expression of Ago proteins in *E. canadensis* (G7). (B) Relative expression of Ago proteins in *E. multilocularis*. Actin gene Emul_190400 was used as reference. (C) Table of *Echinococcus* Ago proteins domains. (D) Cartoon representation of RNAse-like fold coloured by domains of Group 1 *Echinococcus* Ago proteins. (E) Molecular surface representation of Group 1 of *Echinococcus* Ago and pocket binding miRNA. (F) Overview of conserved Group 1 structures and their domain architecture. (G) Relevant conserved and non-conserved amino acid residues of *Echinococcus* Ago proteins. (H) Cartoon representation of proteins from Group 1 of *Echinococcus* Ago: relevant and conserved residues involved in seed binding, mRNA and slicer activity are zoomed. (I) Cartoon representation of proteins from Group 2 of *Echinococcus* Ago: relevant and conserved residues involved in seed binding, mRNA and slicer activity are zoomed. (J) Cartoon representation of proteins from Group 4 of *Echinococcus* Ago: relevant and conserved residues involved in seed binding, mRNA and slicer activity are zoomed. (K) Sequence alignment of amino acid residues involved in seed binding region of the four groups of *Echinococcus*. (L) Table of sequence primers used for RT-qPCR. (PDF 304 kb)
Additional file 9: Single nucleotide polymorphisms in *Echinococcus.* (A) Number and localization of SNPs in genomic regions defined on the basis of gene architecture between the three *Echinococcus* species. (B) Type of substitution caused by SNPs in coding regions between the three *Echinococcus* species. (C) Distribution of missense mutation per each 100 residues of amino acid. (D) Statistical analyses of missense substitution each 100 amino acid residues among the pairs of *Echinococcus* species. The significance of the differences observed in missense SNPs was evaluated using the anova test with a confidence level of 95%. *P*-values belong to Anova test significance. (E) SNPs distribution in KEGGs pathways. Measure of SNP density by calculated as the number of SNPs divided the number of genes associated with the 5 main pathways. (i) Cellular processes. (ii) Environmental Information Processing. (iii) Organismal Systems. (iv) Metabolism. (v) Genetic Information Processing. (PDF 2885 kb)
Additional file 10: Global phylogeny of *Echinococcus* and model species. (A) Phylogeny reconstruction from complete *Echinococcus* mitochondrial genomes. Nucleotide sequences of 12 protein-coding genes were aligned according to Nakao et al. [53]. Phylogenetic analysis was performed using the Maximum Likelihood method based on the JTT matrix-based model. Bootstrap consensus tree was inferred from 100 replicates. Branches corresponding to partitions reproduced in less than 50% bootstrap replicates are collapsed. Mitochondrial genomes used for *Echinococcus* species phylogeny reconstruction: *E. oligarthrus*, NC_009461, Nakao et al., [108]; *E. vogeli*, NC_009462, Nakao et al., [108]*; E. equinus*, AB786665, Nakao et al., [53]; *E. granulosus*, AB786664, Nakao et al., [53]; *E. felidis*, AB732958, Nakao et al., [53]; *E. multilocularis*, NC_000928, Nakao et al., [109]; *E. shiquicus*, NC_009460, Nakao et al., [108]; *E. ortleppi*, NC_011122, Nakao et al., [108]; *E. canadensis* (G6), NC_011121, Nakao et al., [108]; *E. canadensis* (G7), AB235847, Nakao et al., [108]; *E. canadensis* (G7), PRJEB8992, this work; *E. canadensis* (G8), AB235848, Nakao et al., [108]; *E. canadensis* (G10), AB745463, Nakao et al., [53]. (B) Proteins encoded by single-copygenes were analysed using the Maximum Likelihood method based on the JTT matrix-based model. The analysis involved 14 amino acid sequences from 29 single-copy genes. There were a total of 7001 positions in the final dataset. (C) Single-copy coding DNA sequences (CDS). The analysis involved 14 nucleotide sequences from 29 single-copy genes using the Maximum Likelihood method based on the Tamura-Nei model. There were a total of 14,364 positions in the final dataset. All of the positions with less than 60% site coverage were eliminated. Evolutionary analyses were conducted in MEGA5. Hsap: *Homo sapiens*; Mmus: *Mus musculus*; Drer: *Danio rerio*; Bflo: *Branchiostoma floridae*; Dmel: *Drosophila melanogaster*; Cele: *Caenorhabditis elegans*; ECANG7: *Echinococcus canadensis*; Egra: *Echinococcus granulosus*; Emul: *Echinococcus multilocularis*; Gsal: *Gyrodactylus salaris*; Hmic: *Hymenolepis microstoma*; Sman*: Schistosoma mansoni*; Smed*: Schimdtea mediterranea*; TsM1: *Taenia solium.* (D) Venn diagram of shared loci between the different *Echinococcus* species. (i) The number of total SNPs in each species using *E. canadensis* (G7) genome as reference. (ii) The number of total SNPs in each species using *E. multilocularis* genome as reference. (iii) The number of total SNPs in each species using *E. granulosus* (G1) genome as reference. The numbers under each species name indicate the number of SNPs in the species against the corresponding reference genome. The numbers in the overlap region indicate the number of SNPs with the same polymorphism at the same locus between the species. (PDF 530 kb)

## Abbreviations

AgB: Taeniidae antigen
CGIs: CpG Islands
COX1: Cytochrome oxidase 1
CpG: Pair of adjacent CG
DNMT: DNA methyltransferases
ERAD: Endoplasmic reticulum (ER)-associated degradation
GO: Gene Ontology
GPCR: Adhesion G protein-coupled receptors
GPS: GPCR-proteolytic site
Hsp: Heat shock protein
LINE: Long Interspersed Nuclear Elements
LTR: Long terminal repeat
MBD: Methyl binding domain protein
MeCP: Methyl-CpG binding domain proteins
miRNAs: microRNAs
ORF: Open reading frame
PCR: Polymerase chain reaction
piRNAs: piwiRNAs
RISC: RNA-induced silencing complex
SAM: S-adenosylmethionine
SINE: Short Interspersed Nuclear Elements
siRNAs: Small interfering RNAs
SNP: Single nucleotide polymorphism
SNV: Single nucleotide variation
TRIM: Terminal-repeat retrotransposon in miniature
V-ATPase: Vacuolar ATPase
