# Supplementary figures and images for: The Echinococcus canadensis (G7) genome: a key knowledge of parasitic platyhelminth human diseases
Source: BMC Genomics. 2017 Feb 27;18:204. doi: 10.1186/s12864-017-3574-0 (PMC5327563; doi:10.1186/s12864-017-3574-0)

A

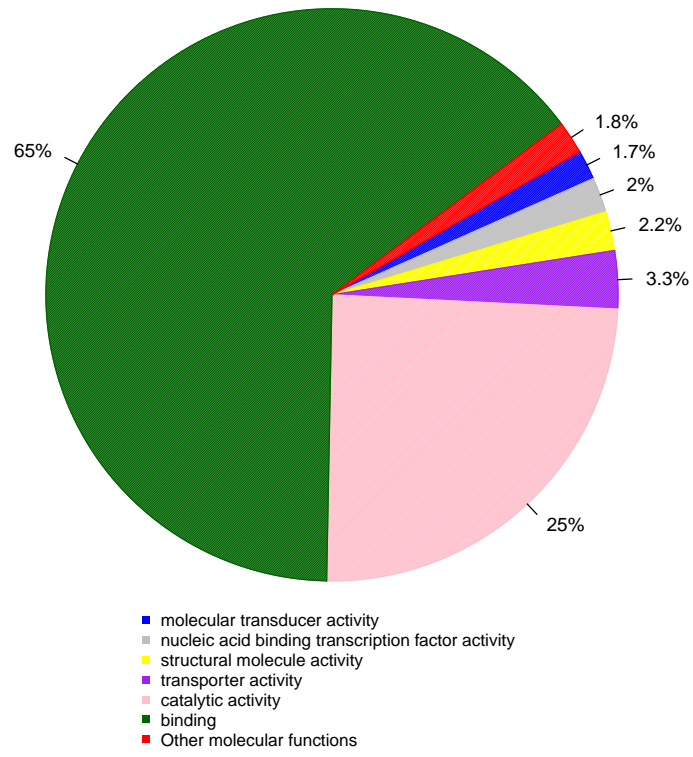

B

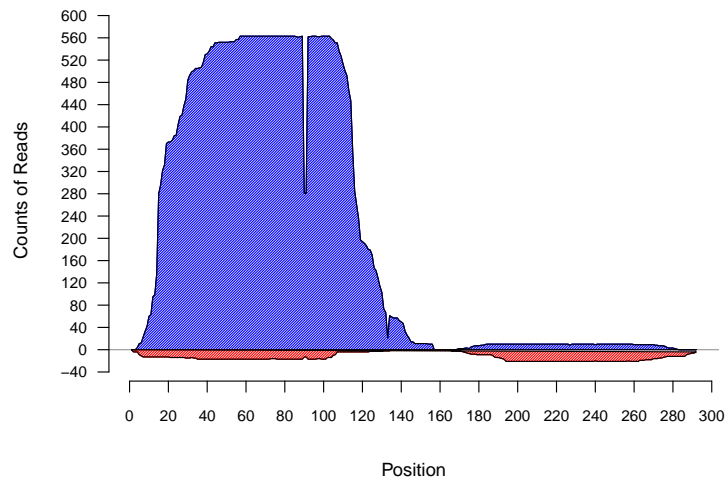

C

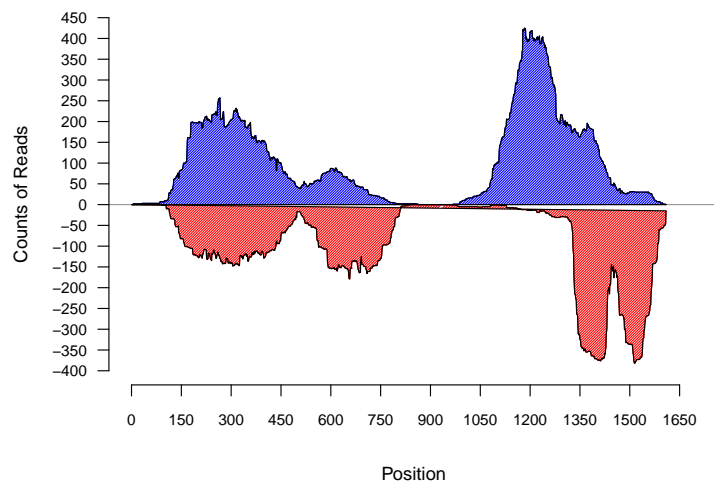

Supplement: Additional file 2: — Echinococcus canadensis (G7) functional annotation and highest repetitive elements read counts. (A) Molecular function GO terms associated frequency of E. canadensis (G7). (B) E.canG7_Brep and (C) E.canG7_rep39 per-base coverage with reads from the forward strand (blue) and reads from the reverse strand (red) are shown. (PDF 295 kb) [file 12864_2017_3574_MOESM2_ESM.pdf]

A

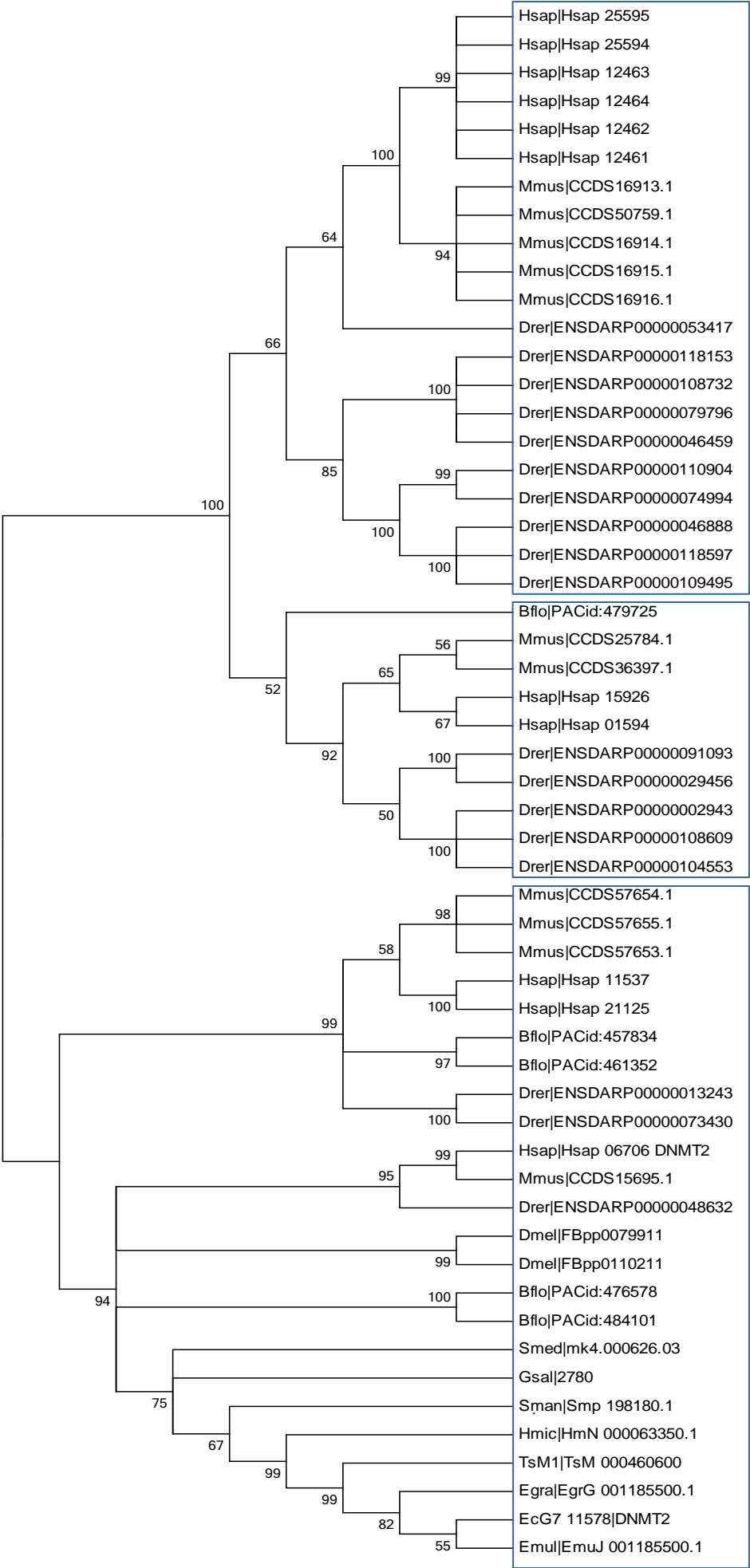

DNMT1

DNMT3

DNMT2

B

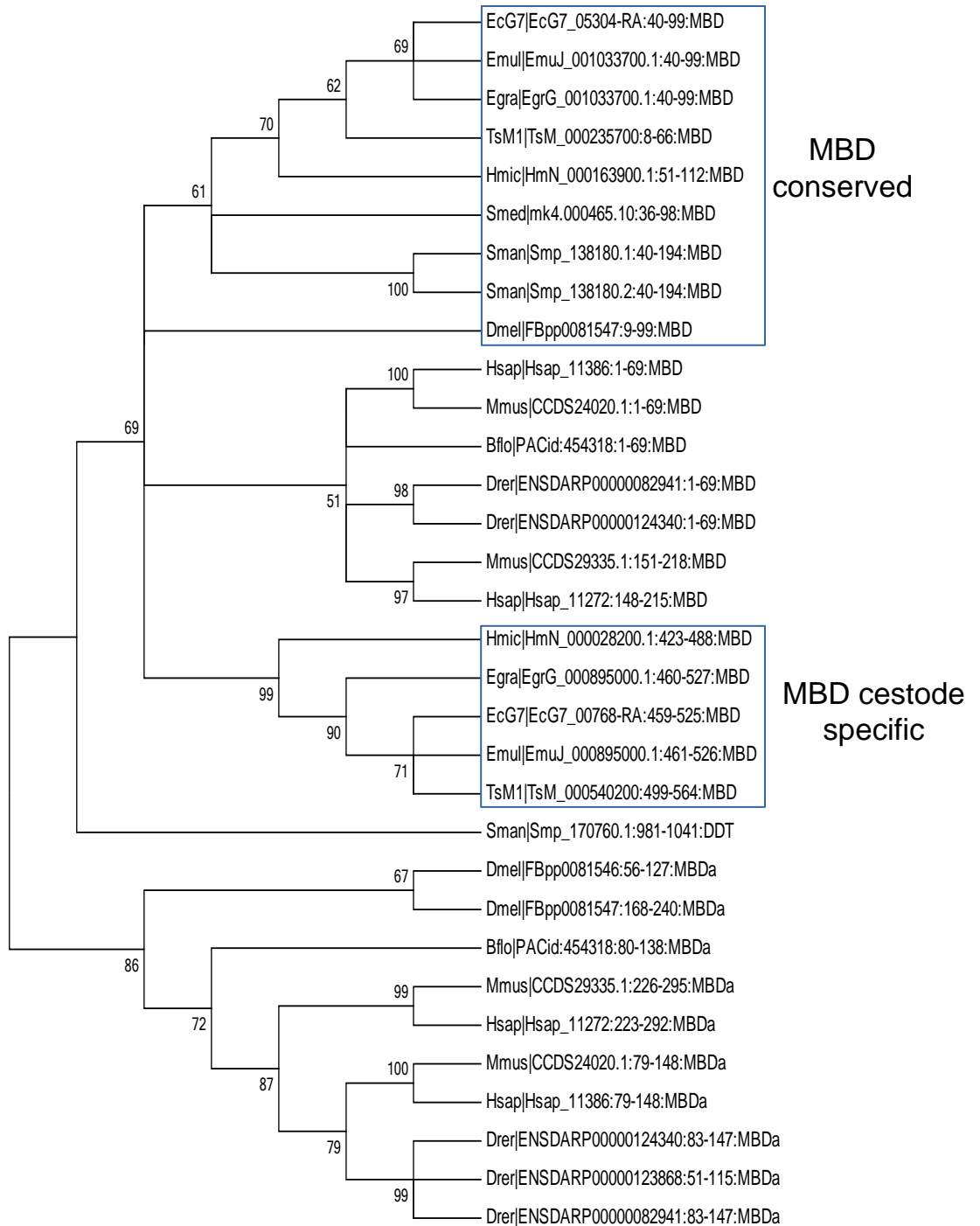

C

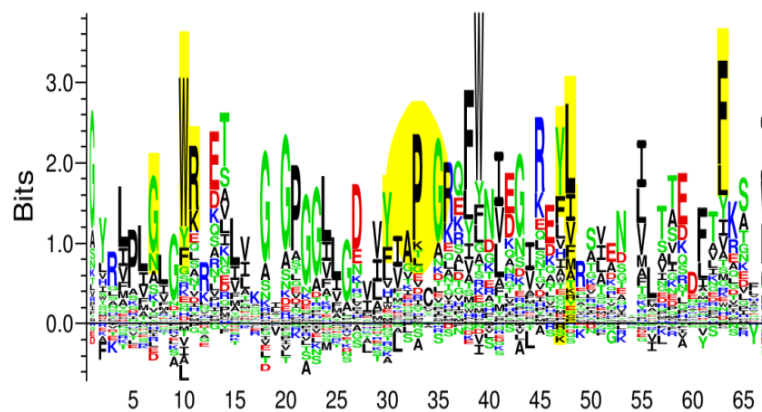

Supplement: Additional file 5: — DNMT and MBD proteins of Echinococcus. (A) Phylogenetic tree of Echinococcus DNMT proteins and classification in family class. (B) Phylogenetic tree of MBD proteins. New MBD protein and families classification. (C) Amino acidic residues involved in MBD motif are shown as logo plots. (PDF 295 kb) [file 12864_2017_3574_MOESM5_ESM.pdf]

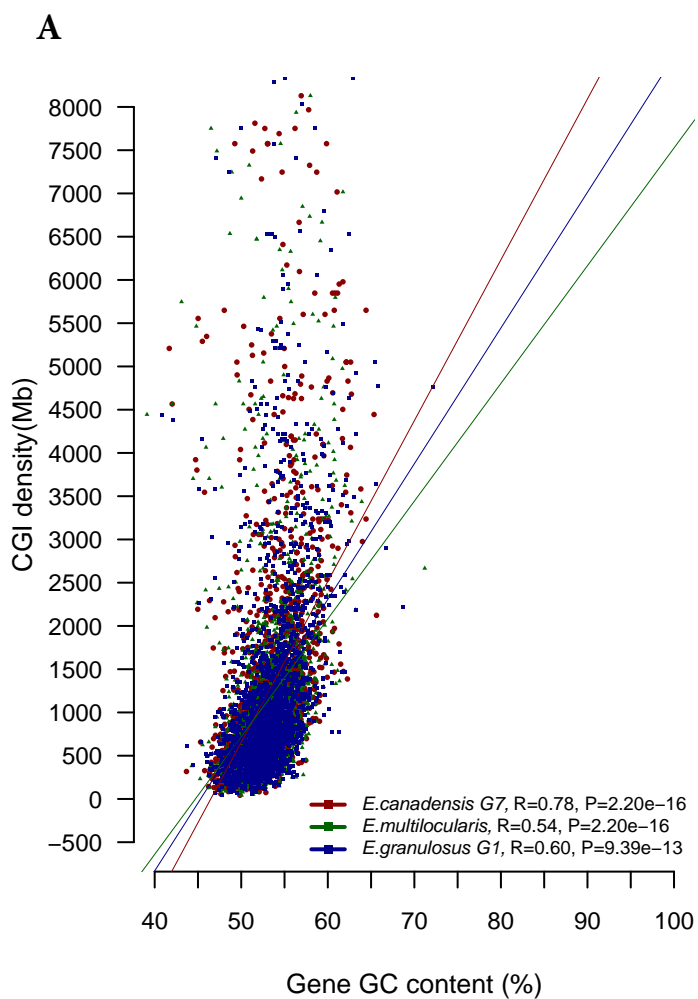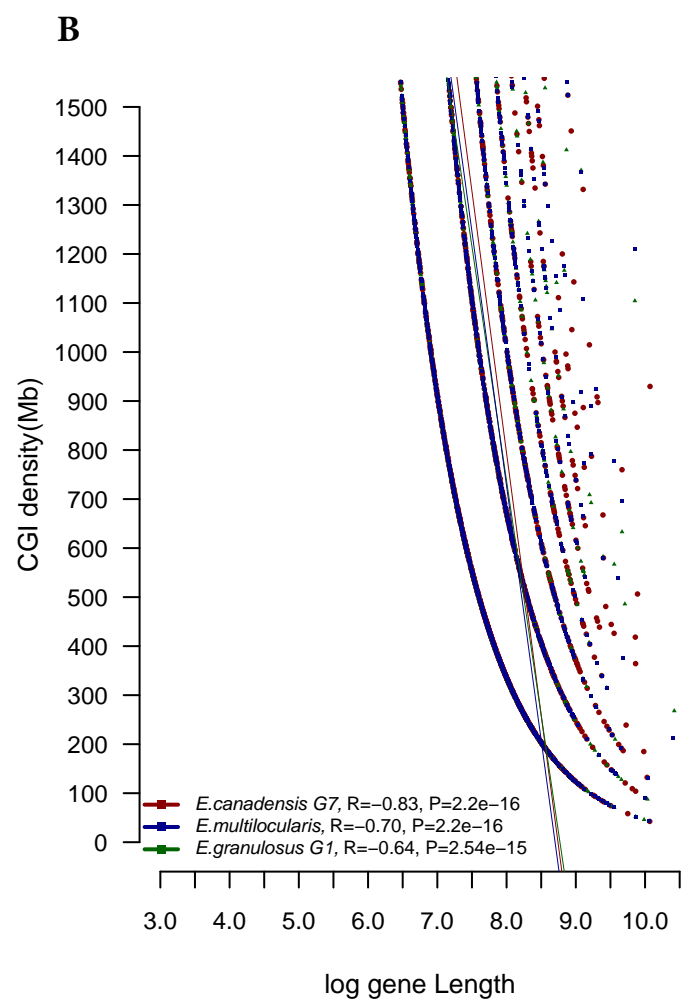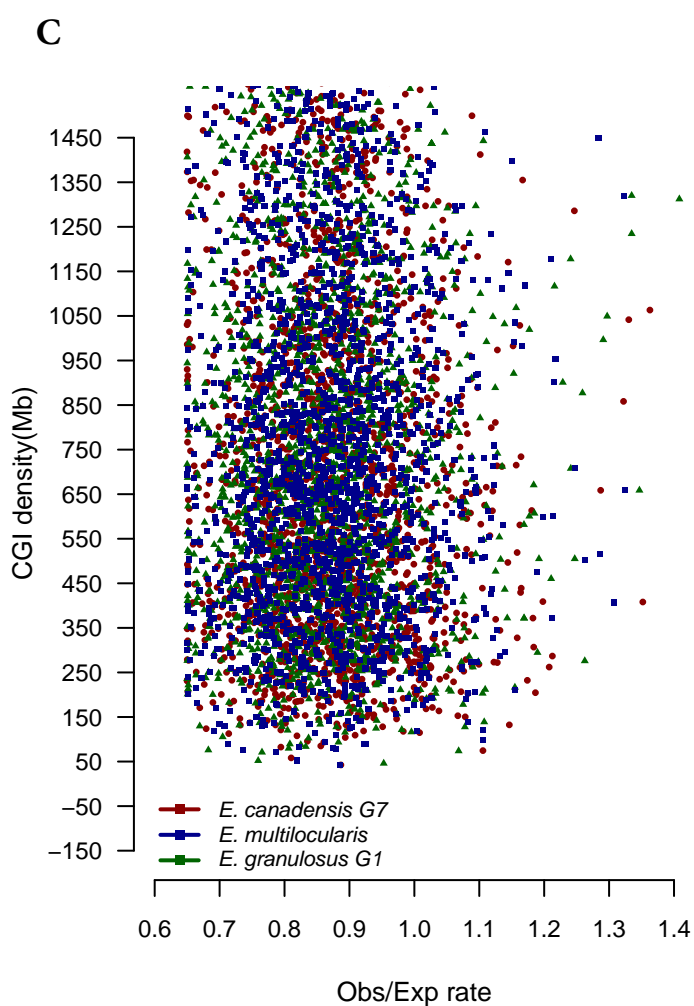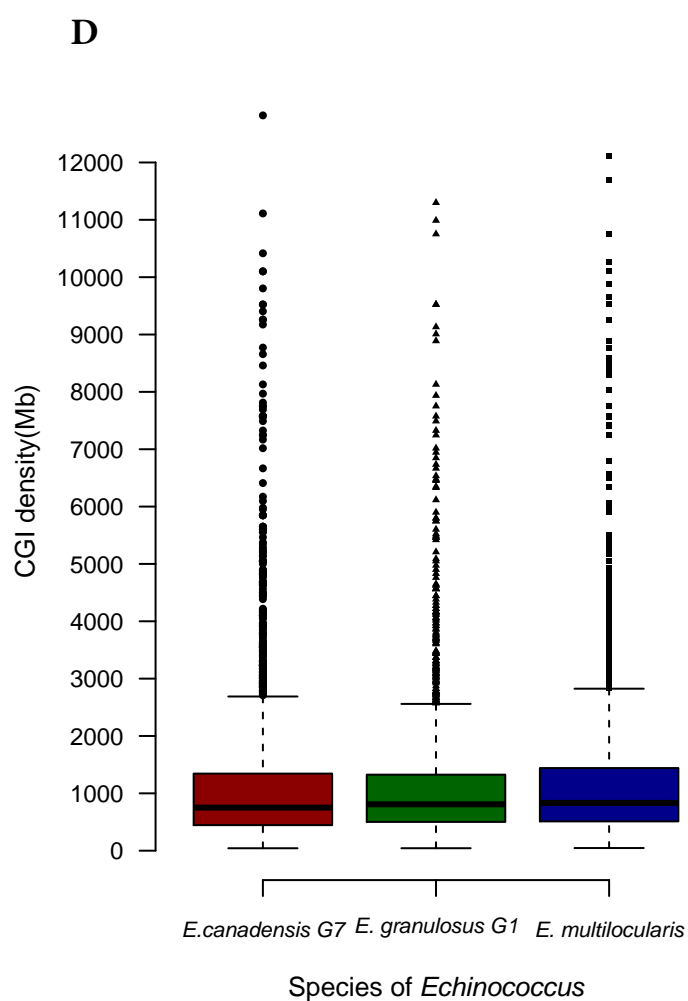

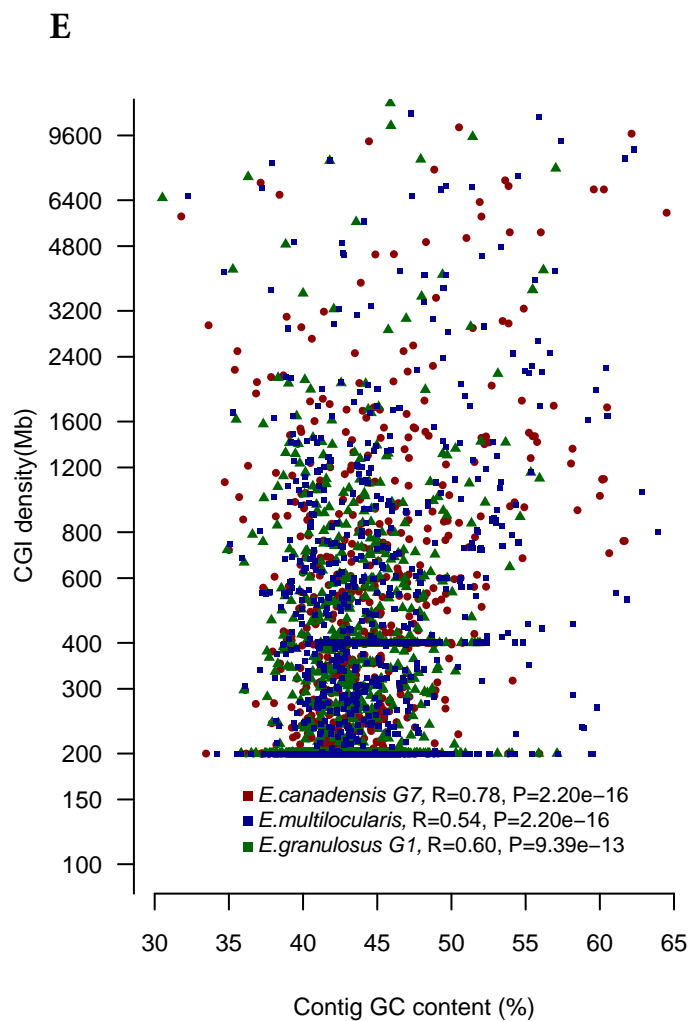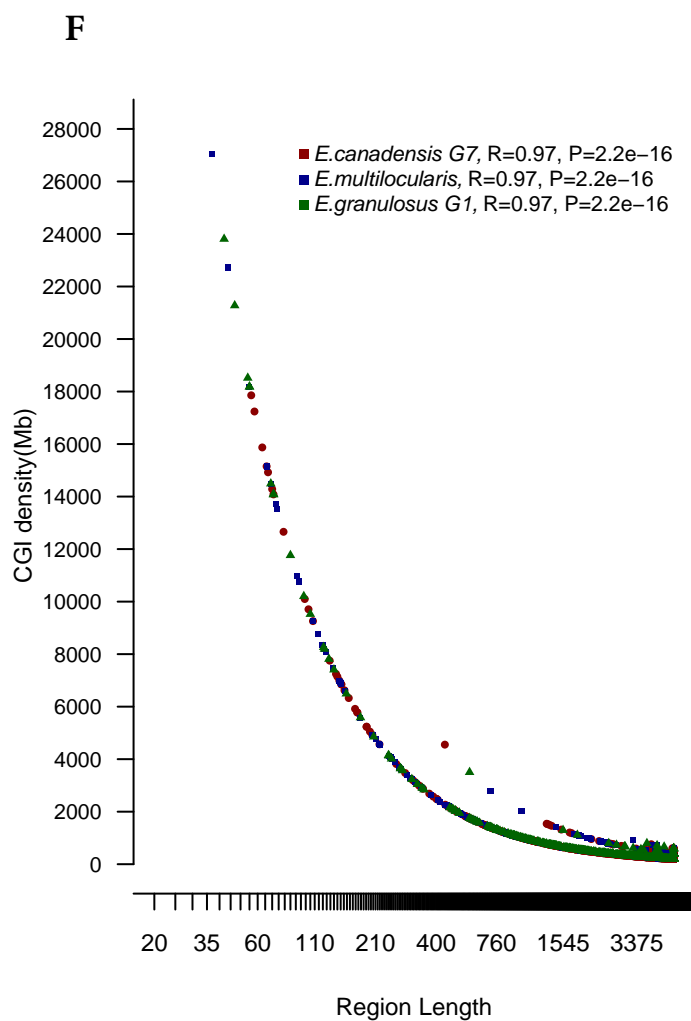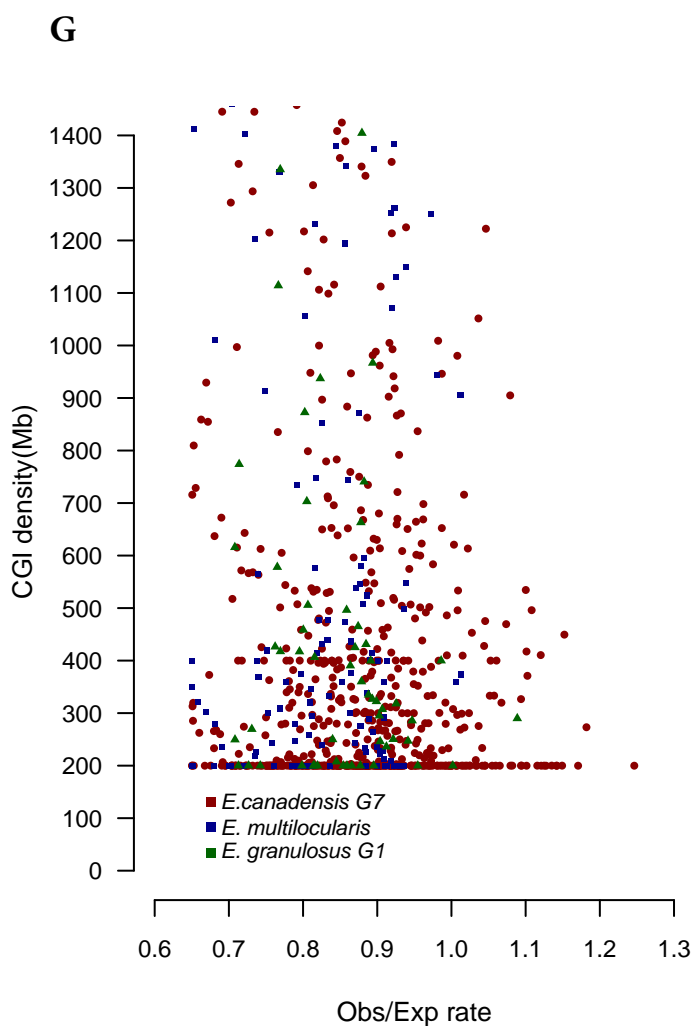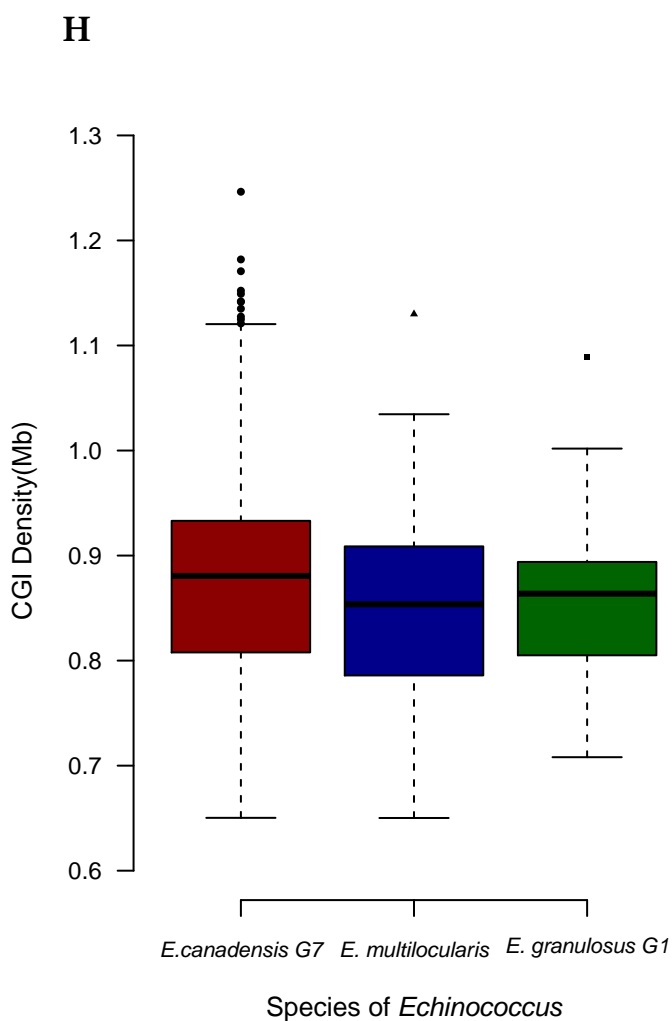

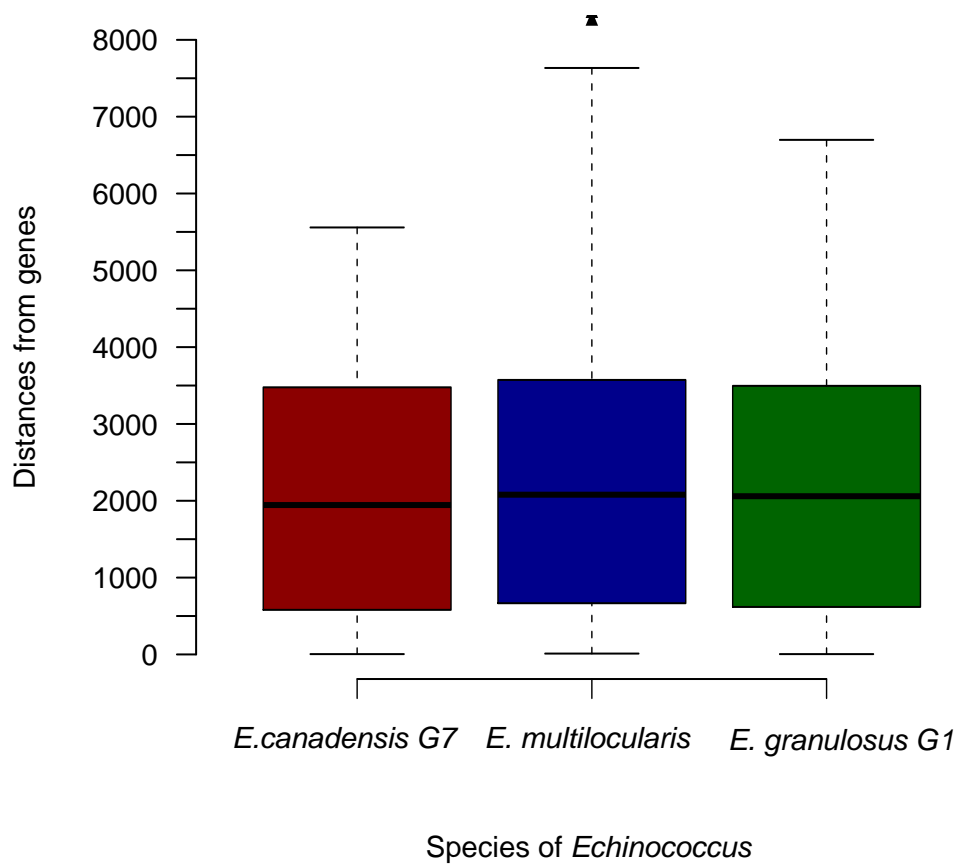

Supplement: Additional file 7: — Correlations between CGI density and coding regions in the genomes of the three Echinococcus species: (A) CGI density (per Mb) versus contig GC content (%). (B) CGI density (per Mb) versus log (contig size). (C) CGI density (per Mb) versus contigs Obs.CpG/Exp.CpG. (D) CGI density (per Mb) by Echinococcus species. Correlations between CGI density and upstream coding regions in the genomes of the three Echinococcus species: (E) CGI density (per Mb) versus contig GC content (%). (F) CGI density (per Mb) versus log (contig size). (G) CGI density (per Mb) versus contigs Obs.CpG/Exp.CpG. (H) CGI density (per Mb) by Echinococcus species. (I) Boxplots of average CGIs distance from the start codon of genes in the three Echinococcus species. (PDF 257 kb) [file 12864_2017_3574_MOESM7_ESM.pdf]

A

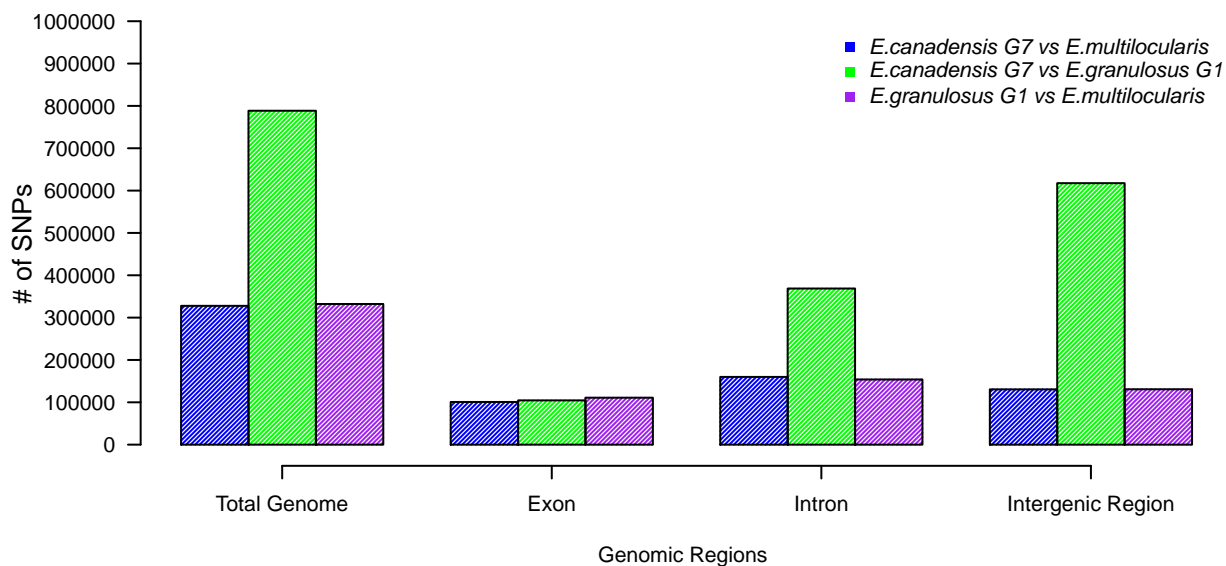

B

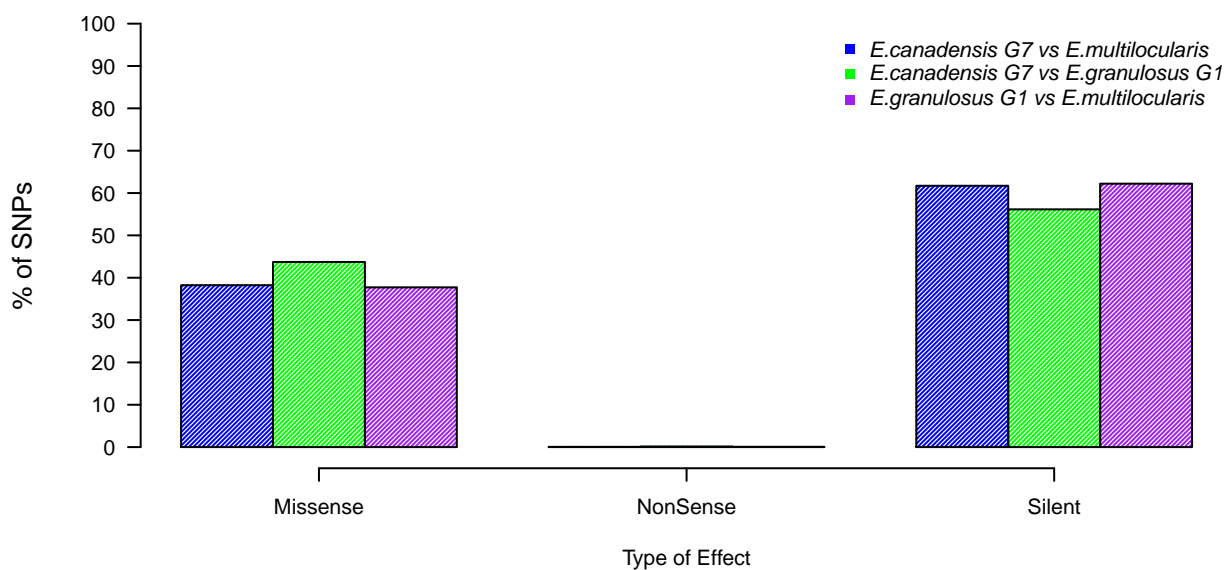

C

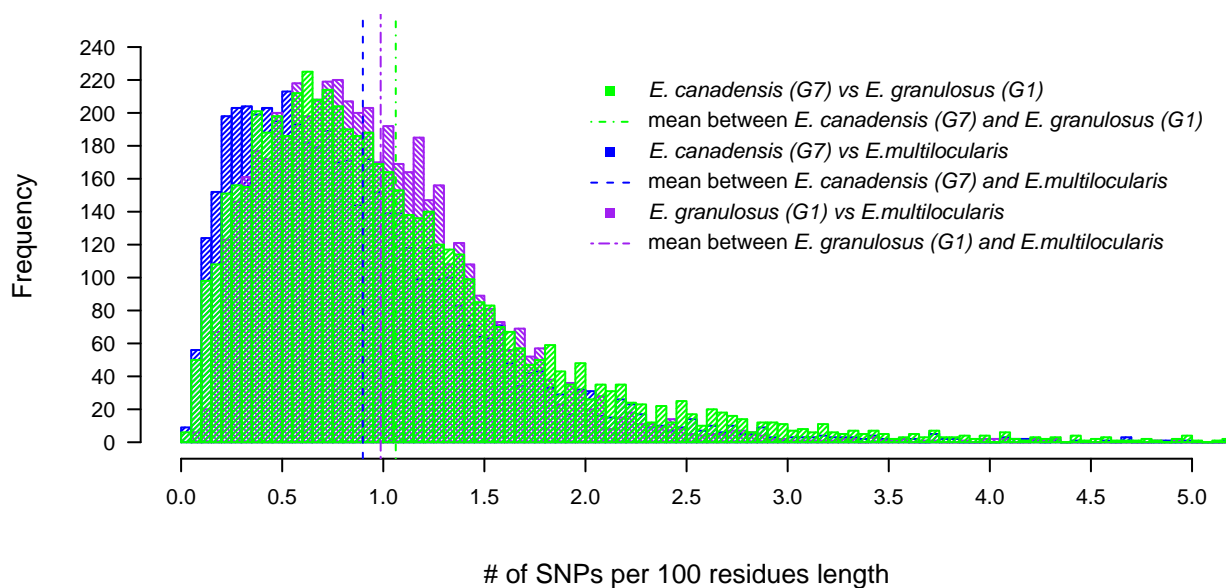

D

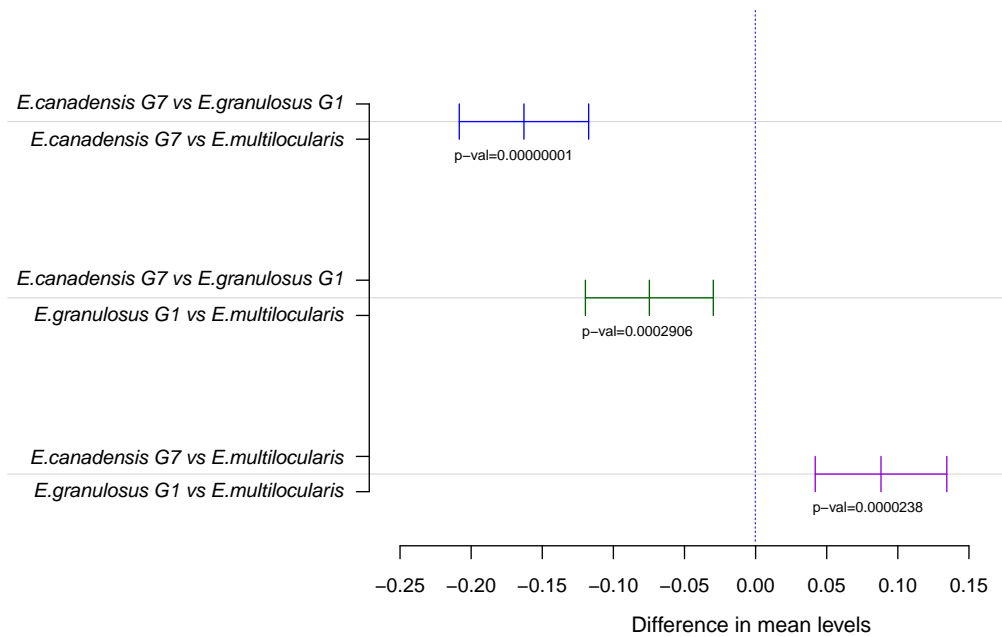

E

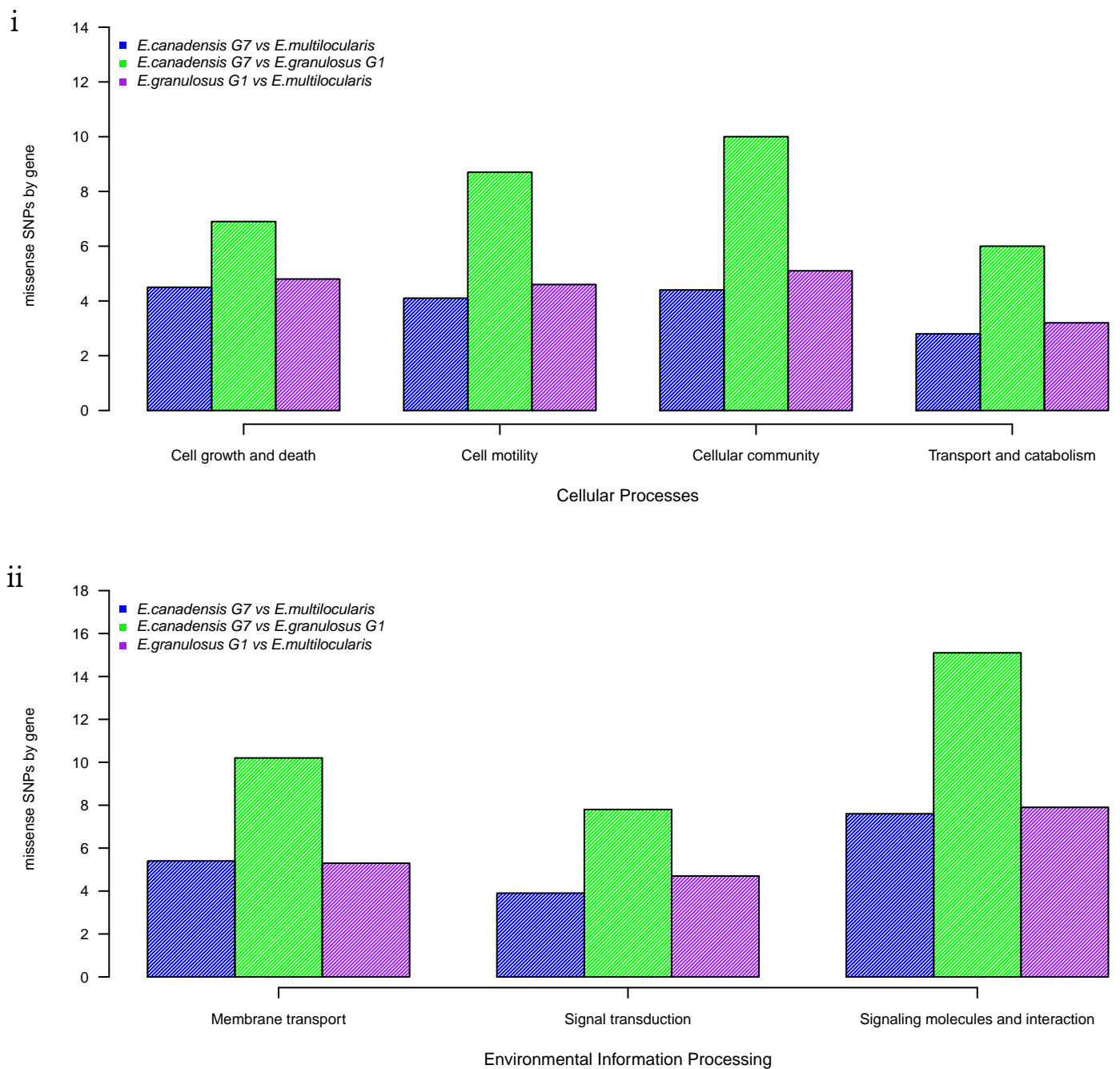

iii

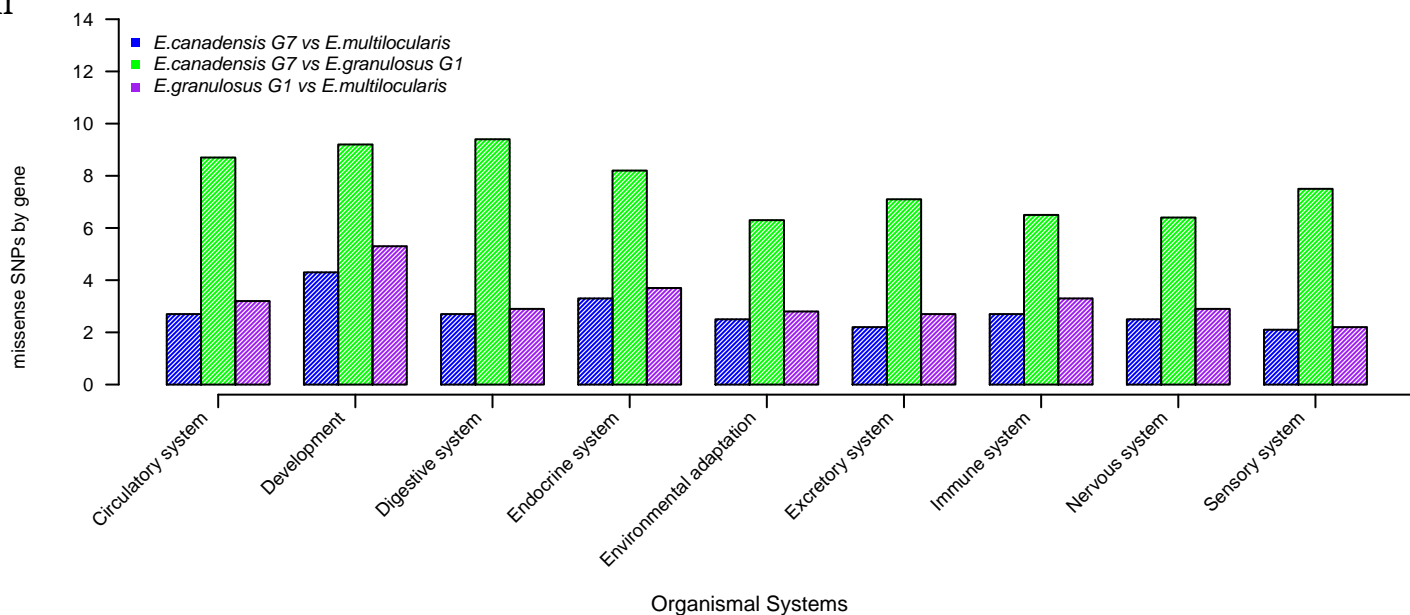

iv

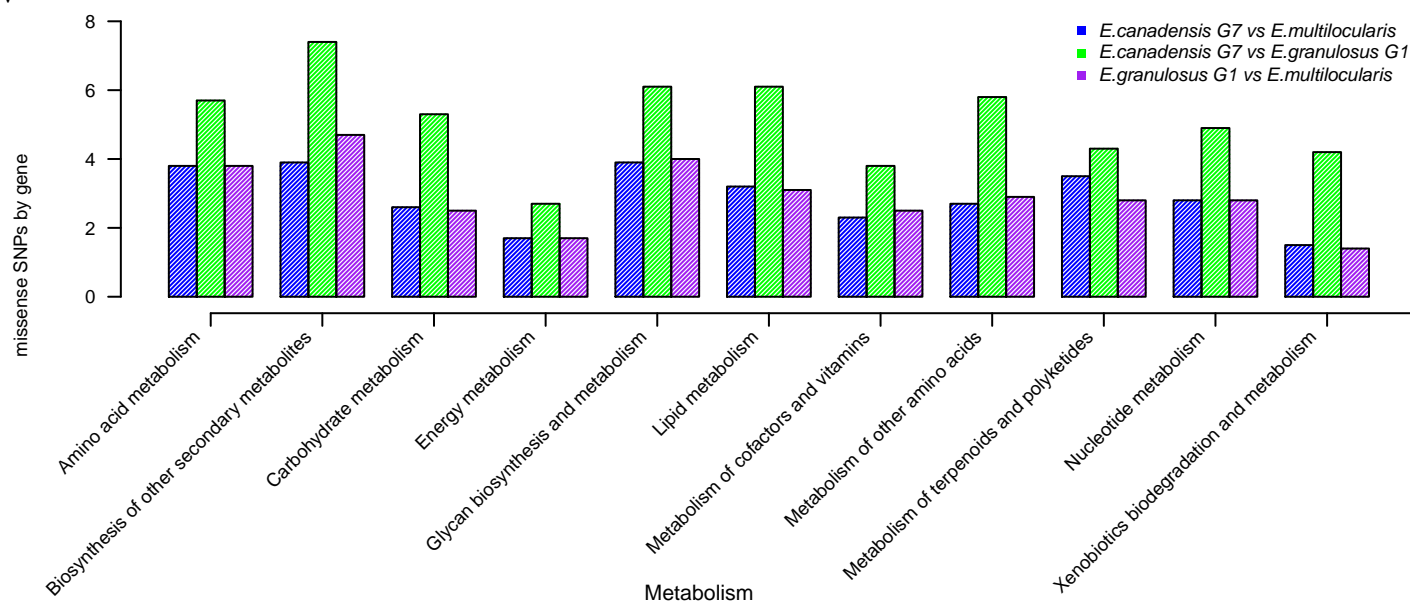

v

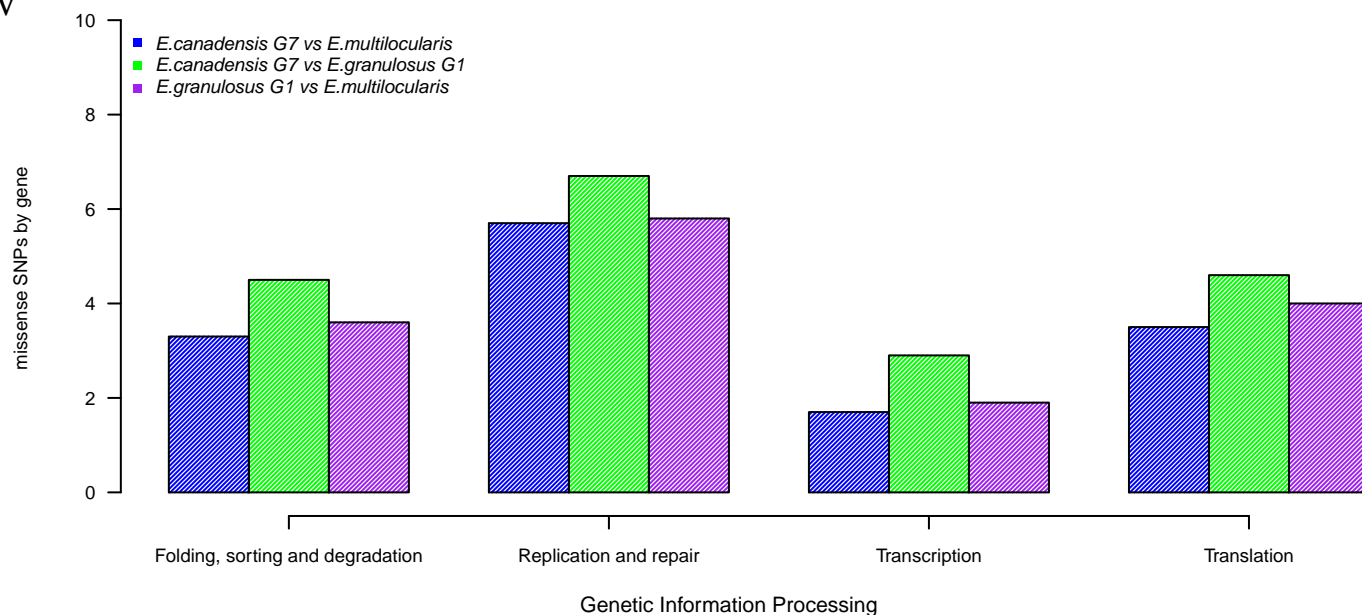

Supplement: Additional file 9: — Single nucleotide polymorphisms in Echinococcus. (A) Number and localization of SNPs in genomic regions defined on the basis of gene architecture between the three Echinococcus species. (B) Type of substitution caused by SNPs in coding regions between the three Echinococcus species. (C) Distribution of missense mutation per each 100 residues of amino acid. (D) Statistical analyses of missense substitution each 100 amino acid residues among the pairs of Echinococcus species. The significance of the differences observed in missense SNPs was evaluated using the anova test with a confidence level of 95%. P-values belong to Anova test significance. (E) SNPs distribution in KEGGs pathways. Measure of SNP density by calculated as the number of SNPs divided the number of genes associated with the 5 main pathways. (i) Cellular processes. (ii) Environmental Information Processing. (iii) Organismal Systems. (iv) Metabolism. (v) Genetic Information Processing. (PDF 2885 kb) [file 12864_2017_3574_MOESM9_ESM.pdf]
