# Supplementary material for: The Echinococcus canadensis (G7) genome: a key knowledge of parasitic platyhelminth human diseases
Source: BMC Genomics. 2017 Feb 27;18:204. doi: 10.1186/s12864-017-3574-0 (PMC5327563; doi:10.1186/s12864-017-3574-0)
Supplement: Additional file 4: — Expanded proteins families and drug targets: Multiple alignment of expanded proteins families and drug target sequences of E. canadensis (G7) and their orthologs. (PDF 999 kb) [file 12864_2017_3574_MOESM4_ESM.pdf]

Expanded protein families in *Echinococcus*

A total of 26 expanded families consisting of 10 to 66 protein members were found in *Echinococcus* (Additional file 1.16). Among them, is the heat-shock protein 70 (Hsp70) family, which has been described by Tsai et al. in all of the tapeworm genomes obtained so far [1]. We also found three interesting expanded families present only in the cestode orthology group: GPS motif-containing protein, Ubiquitin-conjugating enzyme, and Glycosyl transferase. The *E. canadensis* (G7) GPS motif-containing protein is related to polycystin-1, a protein involved in central signal-transduction pathways being the GPS motif (PF01825) responsible of protein-protein interactions. Polycystins form an expanding family of proteins composed of multiple members in fish, invertebrates, mammals and humans. Ubiquitin-conjugating enzyme is known to be involved in the ubiquitination pathway, modulating proteins degradation and protein-protein interactions. The Ubiquitin-conjugating (UBC) complex consists of up to 19 genes in *Echinococcus*. Protein sequence alignments showed a high conservation of the UBC superfamily domain (PF00179) only among cestode parasites. (Figure 1)

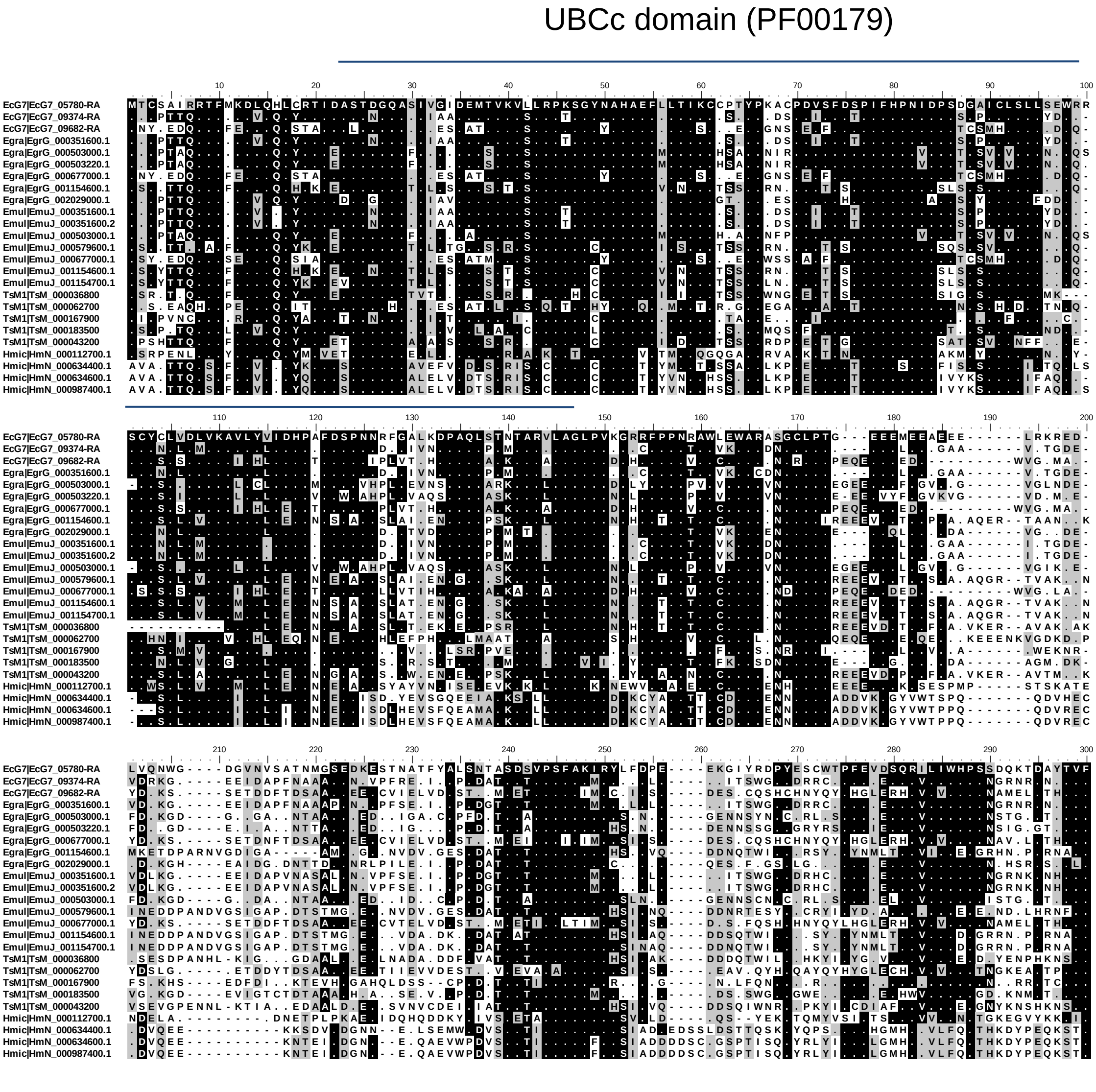

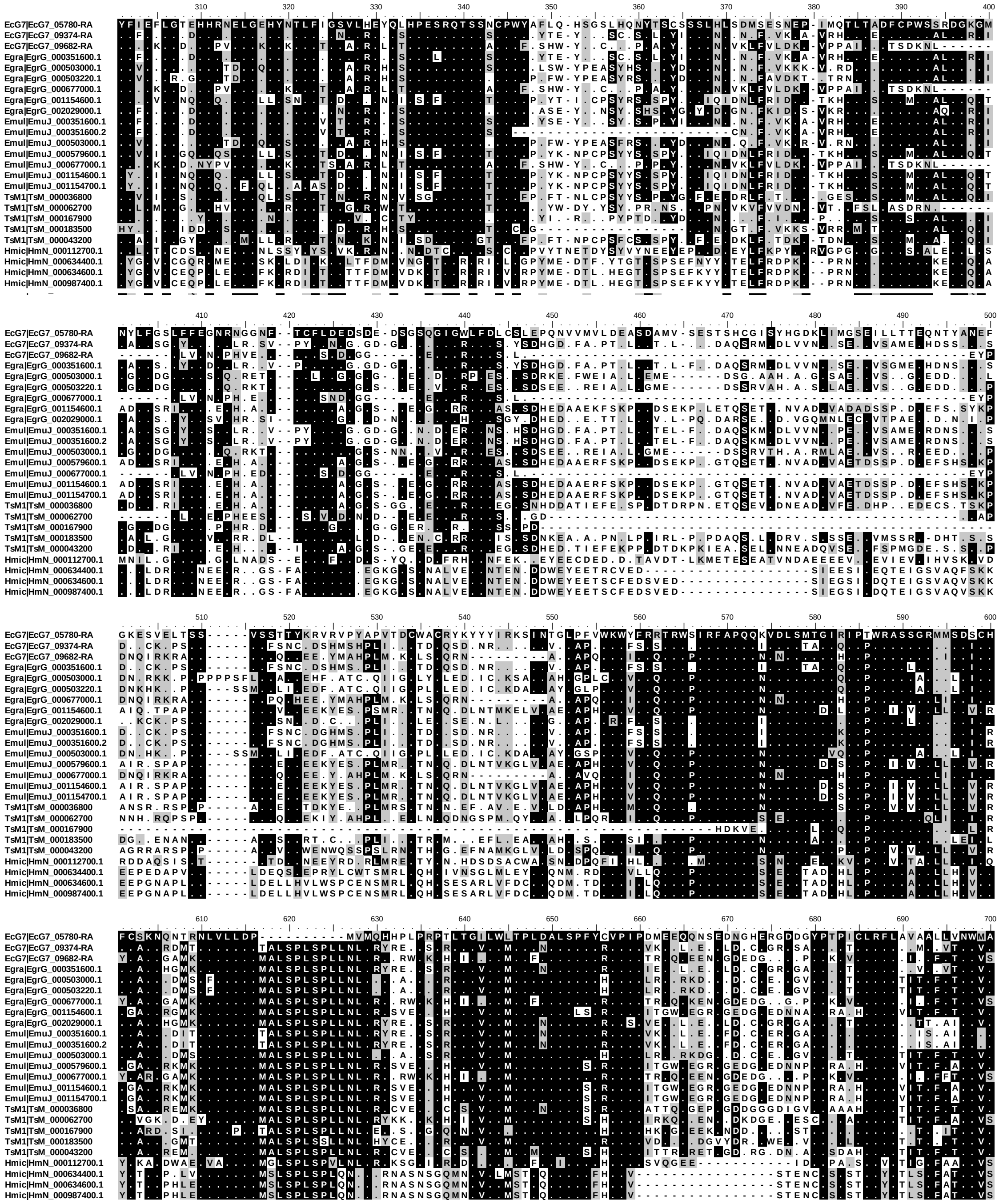

Figure 1: Multiple alignment of *E. canadensis* (G7) expanded proteins. (A) Ubiquitin-conjugating enzyme

The third expanded proteins family is the glycosyl transferases, which is involved in glycan biosynthesis and modifications. This important pathway could play an important role in the biogenesis of the acellular carbohydrate-rich laminated layer, which is a unique *Echinococcus*-specific trait and one of the morphological traits that differs among *Echinococcus* species. These protein families are composed of 10 members that are conserved among cestodes but are very divergent in relation to other organisms. (Figure 2)

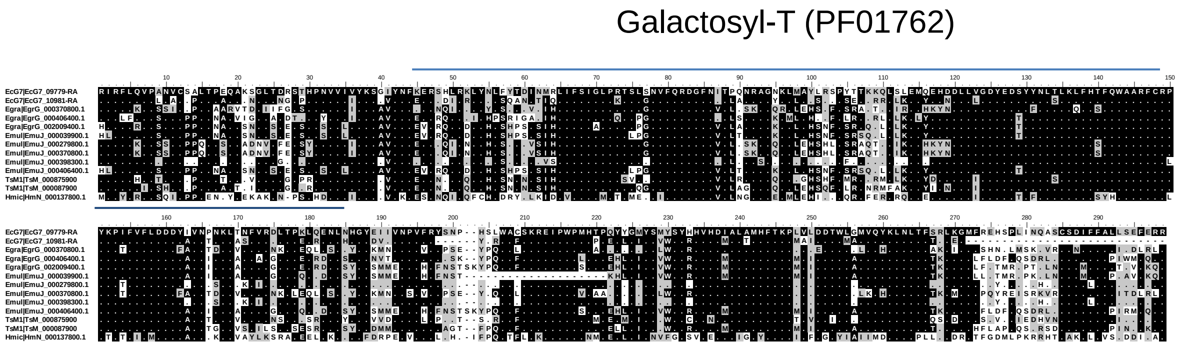

Figure 2: Multiple alignment of *E. canadensis* (G7) expanded proteins (B) Glycosyl transferase enzyme. Protein domains are indicated with Pfam identifier.

Drug targets

Antigens

Antigen B (AgB) is one of the main antigens of cyst hydatid fluid. We have previously demonstrated that it is highly polymorphic and variable in its transcription profile in *E. canadensis* (G7) compared to *E. granulosus* (G1) [2,3]. Important functions have been proposed for this antigen, such as lipid binding and transport [4], and modulation of cell response to inflammation [5]. As expected, we found AgB gene in a cestode-specific orthology group and *E. canadensis* (G7) orthologs to cysteine-type endopeptidase inhibitor (immunogenic protein Ts11), which were previously found in *in vitro* excretion/secretion products of the *T. solium* metacestode [6]. This indicates that there are similarities among the helminth excretion/secretion proteomes, which could be further studied for drug target development.

Antimicrobial peptides

We identified the *E. c*anadensis (G7) “Antimicrobial peptide tachystatin A” (ECANG7_00862), which is present in all of the cestodes but is absent in human hosts. This gene encodes for a 92-amino-acid-long peptide containing a predicted N-terminal signal peptide that resides between the amino acids 1 and 22, thereby suggesting that it could be excreted/secreted. The analysis of the primary sequence of *E. canadensis* (G7) protein exhibited low similarity to the antimicrobial peptide Tachystatin-A2 of *Tachypleus tridentatus* (Arthropoda) (accession number: Q9U8X3) [7] (Figure 3). This protein belongs to the defensin family and has a secondary structure consisting of a cysteine-stabilized triple-stranded beta-sheet [8] and a signal peptide. Defensins are abundant and widely distributed antimicrobial peptides that play an important role in innate immunity and are found in multicellular animals from molluscs to humans. They are characterized by having a cationic β-sheet rich amphipathic structure stabilized by a conserved three-disulfide ligation motif [9]. Indeed, the analysis of the predicted secondary structure of *E. canadensis* (G7) peptide revealed the presence of β-sheet structures with six cysteine residues that could be involved in the stability of the β-sheet structure. In addition, we found that ECANG7_00862 exhibits common physicochemical and structural properties described for antimicrobial peptides: net charge +2, 10 kDa,~ 50% content of hidrobofic amino acids, the presence of signal peptide and short length. Analyses of recently published expression data indicated a high expression level of this peptide in *Echinococcus* [1,10].Amino acid sequence analysis of orthologs of ECANG7_00862 showed that all of the proteins of the

medically relevant related tapeworms share many similarities such as : i) a predicted β-sheet secondary structure is present, ii) the four cysteine residues are conserved, and iii) a C-terminal portion of approximately 23 residues (75-97) is highly conserved.

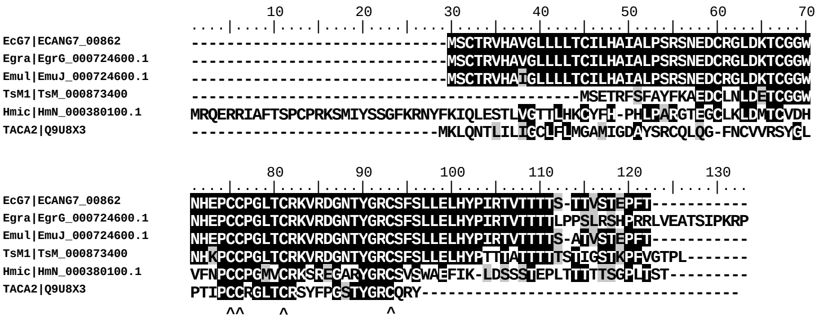

Figure 3: Multiple alignment of drug target sequences of *E. Canadensis* (G7) and their orthologs: (A) Antimicrobial peptides. Arrow heads indicate cysteine residues involved in the β-sheet structures stability.

Peptide hormones

Peptide hormones and neuropeptides are among the most structurally and functionally diverse class of metazoan signalling molecules [11]. The peptides family PP-fold is one of these peptide hormone families [12] and is capable of binding to several G-protein-coupled receptors (GPCRs) [12]. GPCRs are involved in many diseases and are the target of approximately 40% of all of the modern medicinal drugs [13]. These peptides have been identified in *S. mediterranea* and *Schistosoma spp.* and there is evidence of conservation of the genomic organisation of flatworm peptide genes [11]. In this work, the former PP-fold family members were grouped into the “pancreatic hormone peptide” category . In *E. canadensis* (G7) we found four gene models associated with the term "pancreatic hormone peptide-like proteins," that were either present exclusively in cestodes or in flatworms. Two of them, the gene models ECANG7_09023 and ECANG7_05886, encode for a cestode-specific “pancreatic hormone peptide”-like peptide, containing a poorly conserved receptor-binding domain and a dimerization interface. The ECANG7_09023 receptor-binding domain sequence is 100% conserved in *Echinococcus* [1], but has only 55% of identity with the corresponding ortholog of *H. microstoma* [1] and 60% of identity with *S. mediterranea* (Figure 4). The ECANG7_05886 protein domain sequence showed 100% of identity with the corresponding orthologs of *E.multilocularis* and *E. granulosus* (G1), 81% of identity with *H. microstoma* [1] and 75% of identity with *Aplysia californica* (Lophochotrozoa/Mollusca/Gastropoda, the closest invertebrate organism which there is information about) [14]. There is experimental evidence of the transcription of both gene model transcripts in *E. multilocularis* metacestodes, pre-gravid and gravid adult specimens, and of the product of gene model ECANG7_09023 in protoscoleces [1].

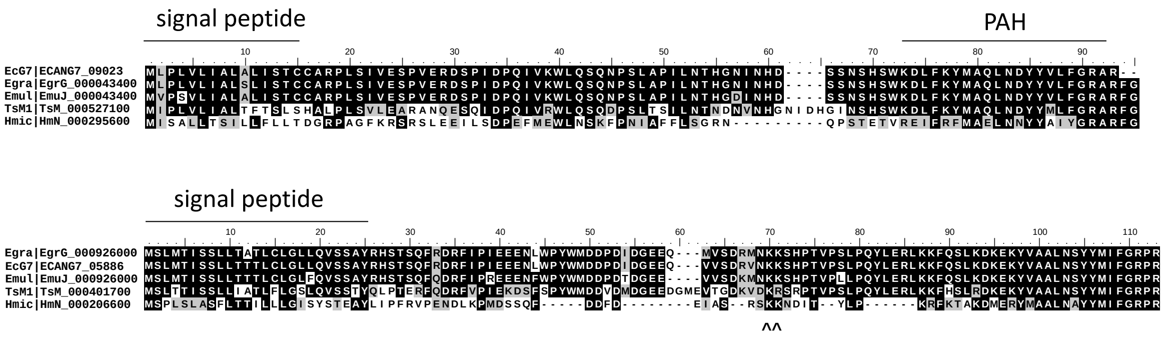

Figure 4: Multiple alignment of drug target sequences of *E. canadensis* (G7) and their orthologs: (B) pancreatic hormones-like proteins The sequence in bold corresponds to the predicted signal peptide found by Signal P 4.1 software, the black line corresponds to the conserved domain found in PFAM database

Transport

Vacuolar ATPase (V-ATPase) is an ubiquitous proton pump of eukaryotic cells that performs essential activities using the localized concentration of protons energized by ATP [15]. V-ATPases play different functions such as receptor-mediated endocytosis, intracellular membrane traffic, protein degradation and coupled transport of small molecules and ions [16,17]. In nematodes it was described to be involved in several functions such as nutrition, osmoregulation, synthesis of the cuticle, neurobiology and reproduction [18]. The presence and the potential role of this protein in platyhelminthes have not yet been explored. In this category, we found 3 gene models that encode for V-ATPases in the *E.canadensis* (G7) genome and their corresponding orthologs in cestodes. Among them; the gene model ECANG7_02132 showed a high conservation of sequence and alpha helix structure in the N-terminal region with the most similar V-ATPase that has been characterised in invertebrates The V-ATPase of tobacco hornworm manduca sexta (Figure 5)[19]

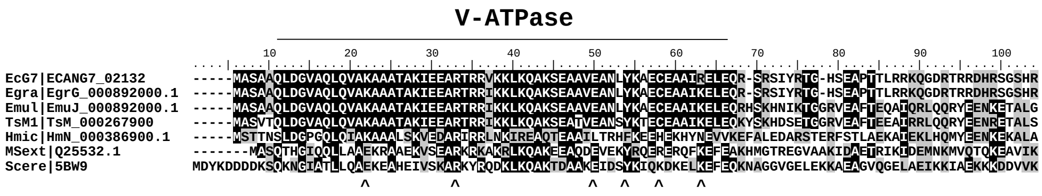

Figure 5: Multiple alignment of drug target sequences of *E. Canadensis* (G7) and their orthologs: (C) V-ATPase protein sequences are added to the alignment. Arrow heads below alignment indicate sV-ATPase conserved residues.

Metabolism

In the metabolism category we found an enzyme involved in glycosylation processes. In eukaryotes, glycosylation is critical for correct protein folding and sorting, as well as for the enzyme activity and quality control involving the endoplasmic reticulum (ER)-associated degradation (ERAD) pathway. Part of glycosylation takes place on the luminal side of the ER, where mannoses and glucoses are transferred to acceptor molecules. Mannosylation in the ER lumen is common to four glycosylation pathways: N-linked glycosylation, glycosylphosphatidylinositol (GPI)-anchor, protein O- and protein C-mannosylation, and it is vital for many eukaryotes [20]. Dolichol-phosphate mannose is a mannosyl donor, which is important for the above-mentioned pathways, and is synthesised from GDP-mannose and dolichol-phosphate by the

enzyme Dolichyl-phosphate beta-D-mannosyl transferase (DPM) (EC 2.4.1.83). In mammals, the enzyme is a complex of three proteins: DPM1, which is the catalytic subunit, and two other subunits, DPM2 and DPM3. DPM3 is necessary for stabilization and ER localization of DPM1, and DPM2 stabilizes DPM3. DPM3 is an ER-localized 92-amino-acid protein composed of two membrane-spanning regions [21]. In *E. canadensis* (G7) we found two subunits of the DPM complex: the subunit DPM1 that is encoded by the gene model ECANG7_04674 and is present in all of the metazoan analysed. And the subunit DPM3 that is encoded by the gene model ECANG7_01023, is present only in cestodes and consist of 2 transmembrane regions and the DMP3 superfamily domain. (Figure 6 A and B). The high sequence conservation in all of the cestode species (90% among *Echinococcus* species and 65% with other cestodes species) and the low sequence conservation in relation to the human counterpart suggest that it codes for a new class of DPM3. Since DPM3 is the only divergent member of the complete metabolic pathway in *Echinococcus* (Figure 6 C) and play a regulatory role in N-glycan precursor biosynhtesis it could represent a novel drug target candidate.

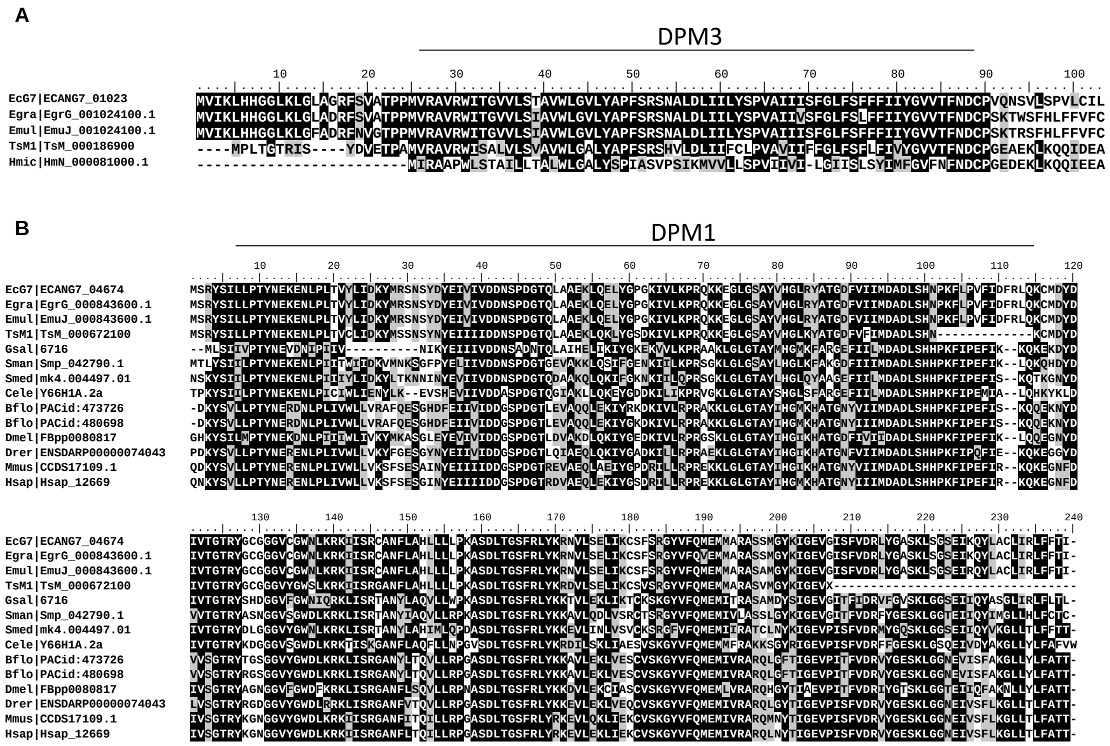

C

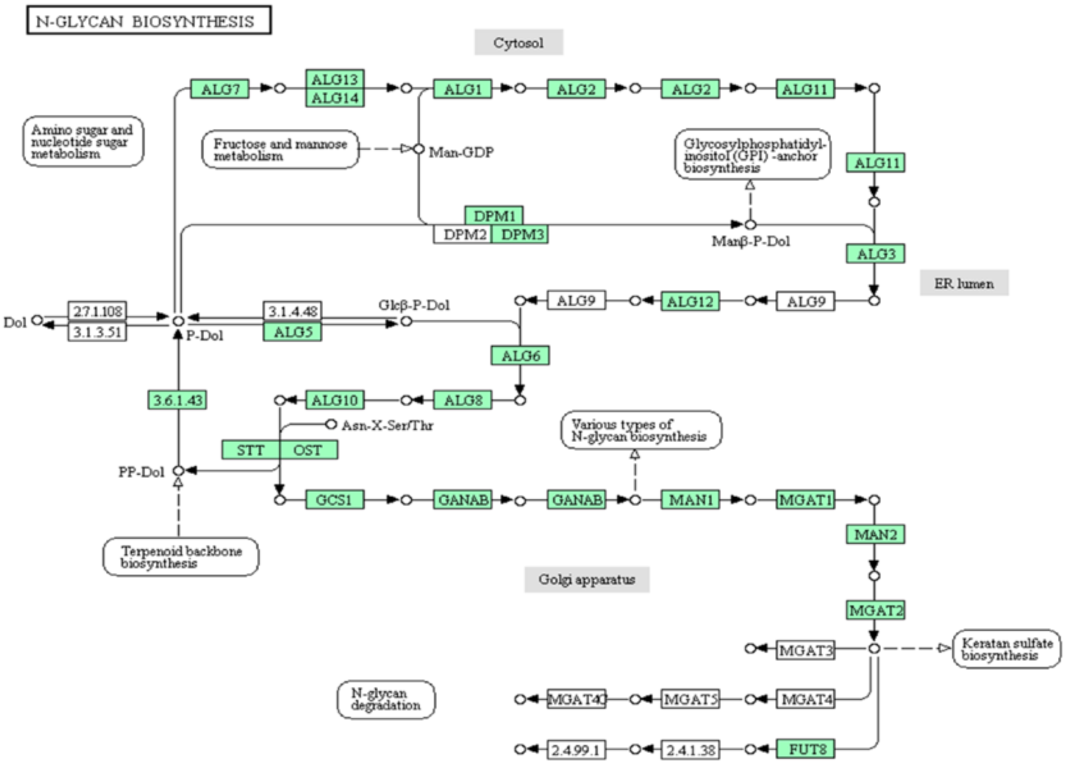

Figure 6: Multiple alignment of drug target sequences of *E. Canadensis* (G7) and their orthologs: (A) DPM3 peptide. The black line over the alignment indicates the Dolichol-phosphate mannosyl transferase subunit 3 domains (DPM3) (B) DPM1 and conserved orthologs of all the organisms. Hsap: *Homo sapiens,* Mmus: *Mus musculus*; Drer: *Danio rerio*; Bflo: *Branchiostoma floridae*; Dmel: *Drosophila melanogaster*; Cele: *Caenorhabditis elegans*; ECANG7: *Echinococcus canadensis*; Egra: *Echinococcus granulosus*; Emul: *Echinococcus multilocularis*; Gsal: *Gyrodactylus salaris*; Hmic: *Hymenolepis microstoma*; Sman*: Schistosoma mansoni*; Smed*: Schimdtea mediterranea*; TsM1: *Taenia solium.*TACA2: *Tachypleus tridentatus* (Tachystatin-A2, GenBank: Q9U8X3) M. sexta and *S. cerevisiae* (Sc). (C) The complete metabolic pathway of N-glican biosynthesis in *Echinococcus*

Transcription processes

Zinc finger proteins are a class of regulatory proteins that participate in a variety of cellular activities, such as development, differentiation and tumour suppression. Among them, C2H2 zinc-finger genes are members of the largest and most complex gene superfamilies in metazoan genomes [22] Subgroups of lineage specific C2H2-containing proteins can be found in yeast, nematodes, insects and plants [23]. This class of zinc fingers can have a variety of functions, such as RNA binding and mediating protein-protein interactions, but are best known due to their role in sequence-specific DNA-binding proteins, in particular in humans, where has been described that they bind specific methylated DNA sequences. Such proteins exhibit zinc finger domains that are typically organised in tandem repeats of two, three or more fingers comprising the DNA-binding domain of the protein. In *E. canadensis* (G7) we found 124 gene models that encode for C2H2-domain containing proteins. Among all of them the gene model ECANG7_07928 encodes for a 125- amino-acid protein that contains 4 C2H2-type zinc finger domains and it is present exclusively in cestodes. Sequences-specific methylated DNA-binding proteins along with genomic DNA methylation pattern may play a role in the regulation of gene transcription.

Bibliography

1. Tsai IJ, Zarowiecki M, Holroyd N, Garciarrubio A, Sanchez-Flores A, Brooks KL, et al. The genomes of four tapeworm species reveal adaptations to parasitism. Nature [Internet]. Nature Publishing Group, a division of Macmillan Publishers Limited. All Rights Reserved.; 2013 [cited 2014 May 29];496:57–63. Available from: http://dx.doi.org/10.1038/nature12031
2. Muzulin PM, Kamenetzky L, Gutierrez AM, Guarnera EA, Rosenzvit MC. Echinococcus granulosus antigen B gene family: Further studies of strain polymorphism at the genomic and transcriptional levels. Exp. Parasitol. 2008;118:156–64.
3. Kamenetzky L, Muzulin PM, Gutierrez AM, Angel SO, Zaha A, Guarnera EA, et al. High polymorphism in genes encoding antigen B from human infecting strains of Echinococcus granulosus. Parasitology. 2005;131:805–15.
4. Silva-Álvarez V, Franchini GR, Pórfido JL, Kennedy MW, Ferreira AM, Córsico B. Lipid-free antigen B subunits from echinococcus granulosus: oligomerization, ligand binding, and membrane interaction properties. PLoS Negl. Trop. Dis. [Internet]. 2015 [cited 2016 Apr 11];9:e0003552. Available from: http://www.ncbi.nlm.nih.gov/pubmed/25768648
5. Silva-Álvarez V, Folle AM, Ramos AL, Kitano ES, Iwai LK, Corraliza I, et al. Echinococcus granulosus Antigen B binds to monocytes and macrophages modulating cell response to inflammation. Parasit. Vectors [Internet]. 2016 [cited 2016 Apr 11];9:69. Available from: http://www.ncbi.nlm.nih.gov/pubmed/26846700
6. Victor B, Dorny P, Kanobana K, Polman K, Lindh J, Deelder AM, et al. Use of expressed sequence tags as an alternative approach for the identification of Taenia solium metacestode excretion/secretion proteins. BMC Res. Notes [Internet]. 2013 [cited 2016 Mar 30];6:224. Available from: http://www.ncbi.nlm.nih.gov/pubmed/23742691
7. Osaki T, Omotezako M, Nagayama R, Hirata M, Iwanaga S, Kasahara J, et al. Horseshoe crab hemocyte-derived antimicrobial polypeptides, tachystatins, with sequence similarity to spider neurotoxins. J. Biol. Chem. [Internet]. 1999 [cited 2016 Mar 30];274:26172–8. Available from: http://www.ncbi.nlm.nih.gov/pubmed/10473569
8. Fujitani N, Kawabata S, Osaki T, Kumaki Y, Demura M, Nitta K, et al. Structure of the antimicrobial peptide tachystatin A. J. Biol. Chem. [Internet]. 2002 [cited 2016 Mar 30];277:23651–7. Available from: http://www.ncbi.nlm.nih.gov/pubmed/11959852
9. Ganz T. Defensins: antimicrobial peptides of innate immunity. Nat. Rev. Immunol. [Internet]. 2003 [cited 2016 Mar 30];3:710–20. Available from: http://www.ncbi.nlm.nih.gov/pubmed/12949495
10. Zheng H, Zhang W, Zhang L, Zhang Z, Li J, Lu G, et al. The genome of the hydatid tapeworm Echinococcus granulosus. Nat. Genet. [Internet]. Nature Publishing Group; 2013 [cited 2014 Jun 17];45:1168–75. Available from: http://dx.doi.org/10.1038/ng.2757
11. Collins JJ, Hou X, Romanova E V, Lambrus BG, Miller CM, Saberi A, et al. Genome-wide analyses reveal a role for peptide hormones in planarian germline development. PLoS Biol. [Internet]. 2010 [cited 2016 Mar 30];8:e1000509. Available from: http://www.ncbi.nlm.nih.gov/pubmed/20967238
12. Berglund MM, Schober DA, Statnick MA, McDonald PH, Gehlert DR. The use of bioluminescence resonance energy transfer 2 to study neuropeptide Y receptor agonist-induced beta-arrestin 2 interaction. J. Pharmacol. Exp. Ther. [Internet]. 2003 [cited 2016 Mar 30];306:147–56. Available from: http://www.ncbi.nlm.nih.gov/pubmed/12665544
13. DAVID FILMORE. Cell-based screening assays and structural studies are fueling G-protein coupled receptors as one of the most popular classes of investigational drug

targets. Mod. DRUG Discov. [Internet]. 2004 [cited 2016 Mar 30];7:4. Available from: http://pubs.acs.org/subscribe/journals/mdd/v07/i11/pdf/1104feature_filmore.pdf
14. Rajpara SM, Garcia PD, Roberts R, Eliassen JC, Owens DF, Maltby D, et al. Identification and molecular cloning of a neuropeptide Y homolog that produces prolonged inhibition in Aplysia neurons. Neuron [Internet]. 1992 [cited 2016 Mar 30];9:505–13. Available from: http://www.ncbi.nlm.nih.gov/pubmed/1524828
15. Hinton A, Bond S, Forgac M. V-ATPase functions in normal and disease processes. Pflügers Arch. Eur. J. Physiol. [Internet]. 2009 [cited 2016 Mar 30];457:589–98. Available from: http://www.ncbi.nlm.nih.gov/pubmed/18026982
16. Forgac M. Structure, function and regulation of the vacuolar (H+)-ATPases. FEBS Lett. [Internet]. 1998 [cited 2016 Mar 30];440:258–63. Available from: http://www.ncbi.nlm.nih.gov/pubmed/9872382
17. Forgac M. The vacuolar H+-ATPase of clathrin-coated vesicles is reversibly inhibited by S-nitrosoglutathione. J. Biol. Chem. [Internet]. 1999 [cited 2016 Mar 30];274:1301–5. Available from: http://www.ncbi.nlm.nih.gov/pubmed/9880499
18. Knight AJ, Behm CA. Minireview: the role of the vacuolar ATPase in nematodes. Exp. Parasitol. [Internet]. 2012 [cited 2016 Mar 30];132:47–55. Available from: http://www.ncbi.nlm.nih.gov/pubmed/21959022
19. Lepier A, Gräf R, Azuma M, Merzendorfer H, Harvey WR, Wieczorek H. The peripheral complex of the tobacco hornworm V-ATPase contains a novel 13-kDa subunit G. J. Biol. Chem. [Internet]. 1996 [cited 2016 Mar 30];271:8502–8. Available from: http://www.ncbi.nlm.nih.gov/pubmed/8626552
20. Kinoshita T, Fujita M, Maeda Y. Biosynthesis, remodelling and functions of mammalian GPI-anchored proteins: recent progress. J. Biochem. [Internet]. 2008 [cited 2016 Mar 30];144:287–94. Available from: http://www.ncbi.nlm.nih.gov/pubmed/18635593
21. Maeda Y, Tanaka S, Hino J, Kangawa K, Kinoshita T. Human dolichol-phosphate-mannose synthase consists of three subunits, DPM1, DPM2 and DPM3. EMBO J. [Internet]. 2000 [cited 2016 Mar 30];19:2475–82. Available from: http://www.ncbi.nlm.nih.gov/pubmed/10835346
22. Knight RD, Shimeld SM. Identification of conserved C2H2 zinc-finger gene families in the Bilateria. Genome Biol. [Internet]. BioMed Central; 2001 [cited 2016 Oct 7];2:RESEARCH0016. Available from: http://www.ncbi.nlm.nih.gov/pubmed/11387037
23. Seetharam A, Bai Y, Stuart GW, Knight R, Shimeld S, Stillman J, et al. A survey of well conserved families of C2H2 zinc-finger genes in Daphnia. BMC Genomics [Internet]. BioMed Central; 2010 [cited 2016 Oct 7];11:276. Available from: http://bmcgenomics.biomedcentral.com/articles/10.1186/1471-2164-11-276
