# Supplementary material for: The Echinococcus canadensis (G7) genome: a key knowledge of parasitic platyhelminth human diseases
Source: BMC Genomics. 2017 Feb 27;18:204. doi: 10.1186/s12864-017-3574-0 (PMC5327563; doi:10.1186/s12864-017-3574-0)
Supplement: Additional file 8: — Argonaute proteins in Echinococcus. (A) Relative expression of Ago proteins in E. canadensis (G7). (B) Relative expression of Ago proteins in E. multilocularis. Actin gene Emul_190400 was used as reference. (C) Table of Echinococcus Ago proteins domains. (D) Cartoon representation of RNAse-like fold coloured by domains of Group 1 Echinococcus Ago proteins. (E) Molecular surface representation of Group 1 of Echinococcus Ago and pocket binding miRNA. (F) Overview of conserved Group 1 structures and their domain architecture. (G) Relevant conserved and non-conserved amino acid residues of Echinococcus Ago proteins. (H) Cartoon representation of proteins from Group 1 of Echinococcus Ago: relevant and conserved residues involved in seed binding, mRNA and slicer activity are zoomed. (I) Cartoon representation of proteins from Group 2 of Echinococcus Ago: relevant and conserved residues involved in seed binding, mRNA and slicer activity are zoomed. (J) Cartoon representation of proteins from Group 4 of Echinococcus Ago: relevant and conserved residues involved in seed binding, mRNA and slicer activity are zoomed. (K) Sequence alignment of amino acid residues involved in seed binding region of the four groups of Echinococcus. (L) Table of sequence primers used for RT-qPCR. (PDF 304 kb) [file 12864_2017_3574_MOESM8_ESM.pdf]

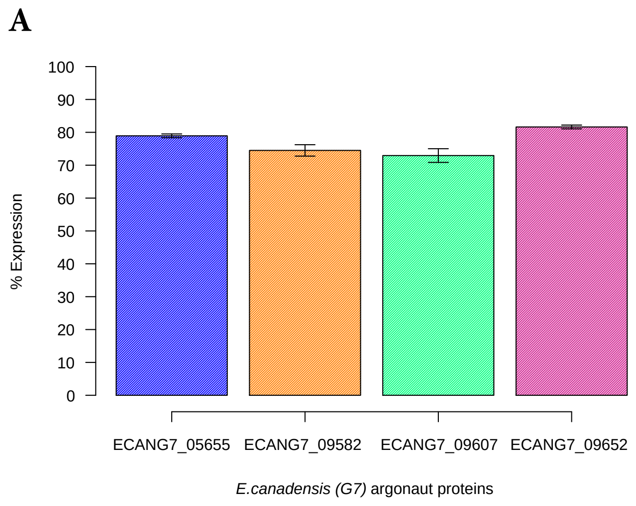

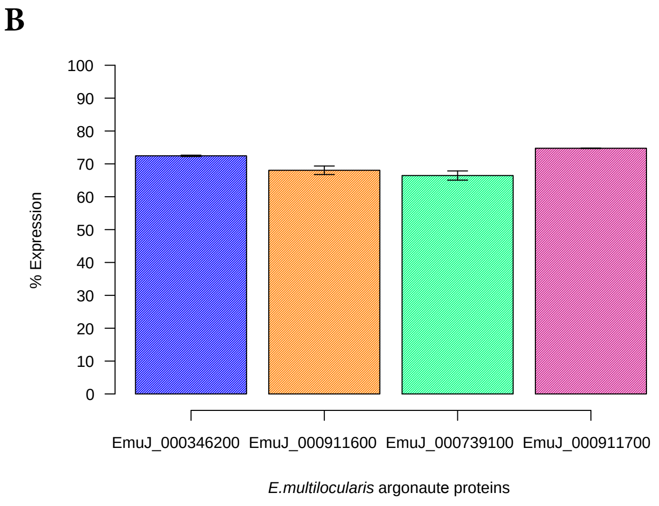

C

***Echinococcus* Ago proteins domain.**

Group	Domain	Pfam Id	Gene Id
Group 1	Piwi	PF02171.14	EcG7_05655, EmuJ_000346200, EgrG_000346200
	ArgoMid	PF16487.2	
	PAZ	PF02170.19	
	ArgoN	PF16486.2	
	ArgoL1	PF08699.7	
	ArgoL2	PF16488.2	
Group 2	Piwi	PF02171.14	EcG7_09582, EmuJ_000911600, EgrG_000911600
	PAZ	PF02170.19	
	ArgoN	PF16486.2	
	ArgoL1/ACT_6 (*)	PF08699.7 / PF13740.3 (*)	
	ArgoL2	PF16488.2	
Group 3	Piwi	PF02171.14	EcG7_09607, EmuJ_000739100, EgrG_000739100
	PAZ	PF02170.19	
Group 4	Piwi	PF02171.14	EcG7_09652, EmuJ_000911700, EgrG_000911700
	PAZ	PF02170.19	
	ArgoL1	PF08699.7	
	ArgoN	PF16486.2	

(*) EmuJ_000911600 belonging to E. *multilocularis*, contain ArgoL1 domain. EcG7_09582 and EgrG_000911600 belonging to E. *canadensis* (G7) and E. *granulosus* (G1) respectevely contain ACT_6 domain instead.

D

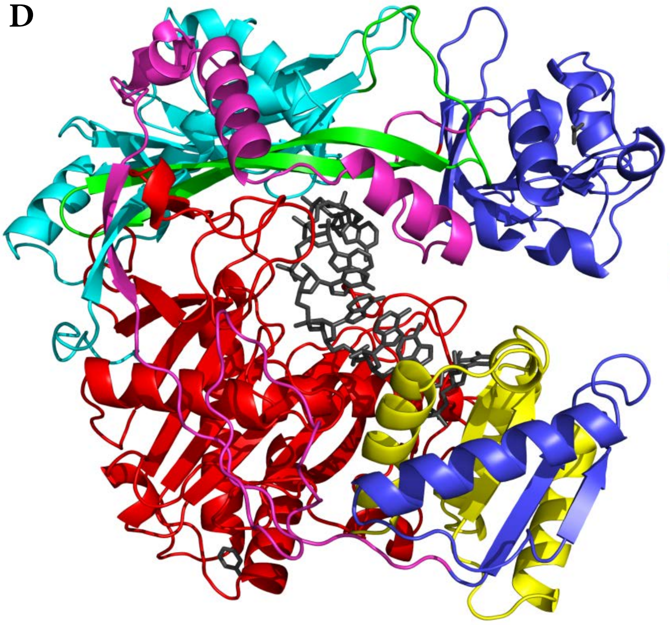

E

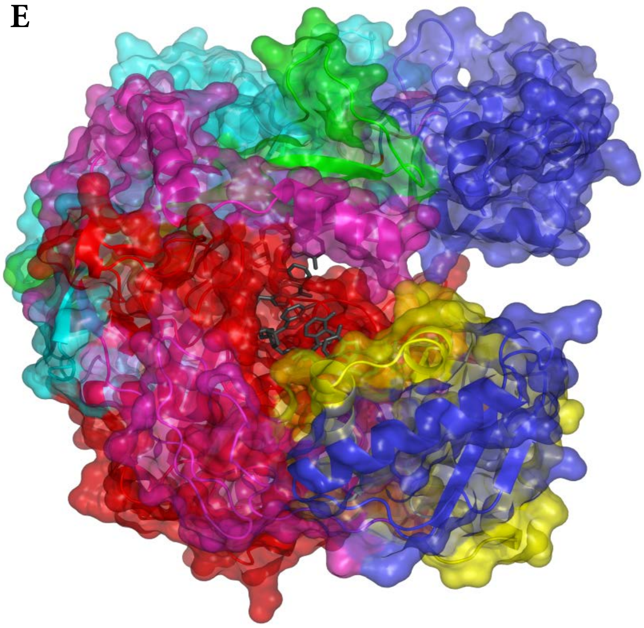

F

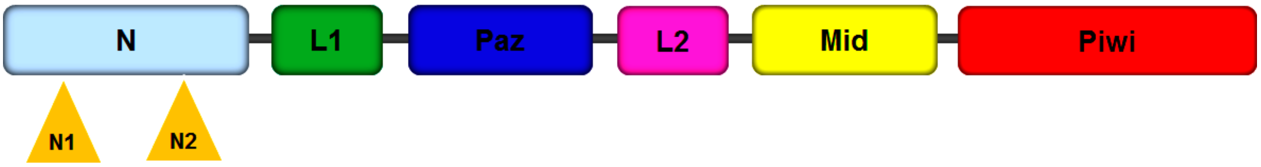

G

Echinococcus **Ago proteins groups. Conserved and non conserved relevant residues.**

Human-Ago2	Human-Ago1	Human-Ago3	Human-Ago4	Hiwi	Miwi	Group 1	Group 2	Group 3	Group 4	Domains
Y529	+	+	+	+	+	+	+	+	+	PAZ
K533	+	+	+	+	+	+	+	+	+	
Q548	+	+	+	V	V	+	K	K	K	
N551	+	+	+	+	+	+	T	T	T	
L563	+	+	+	+	+	+	+	+	+	MID
K566	+	+	+	+	+	+	+	+	+	
K570	+	+	+	+	+	+	+	+	+	
Q633	R	R	R	D	D	S	D	D	D	PIWI
R710	+	+	+	+	+	+	+	+	+	
Q757	+	+	+	+	+	+	+	+	+	
R761	R	+	+	S	S	+	K	K	K	
R792	+	+	+	+	+	+	+	+	+	
C793	+	+	+	+	+	+	+	+	+	
S798	+	+	+	+	+	+	+	+	+	
Y804	+	+	+	+	+	+	+	+	+	
R812	+	+	+	+	+	+	+	+	+	

Conservation is represented by (+) in the aminoacid position, and a change in position is represented by single-letter amino acid code.

H

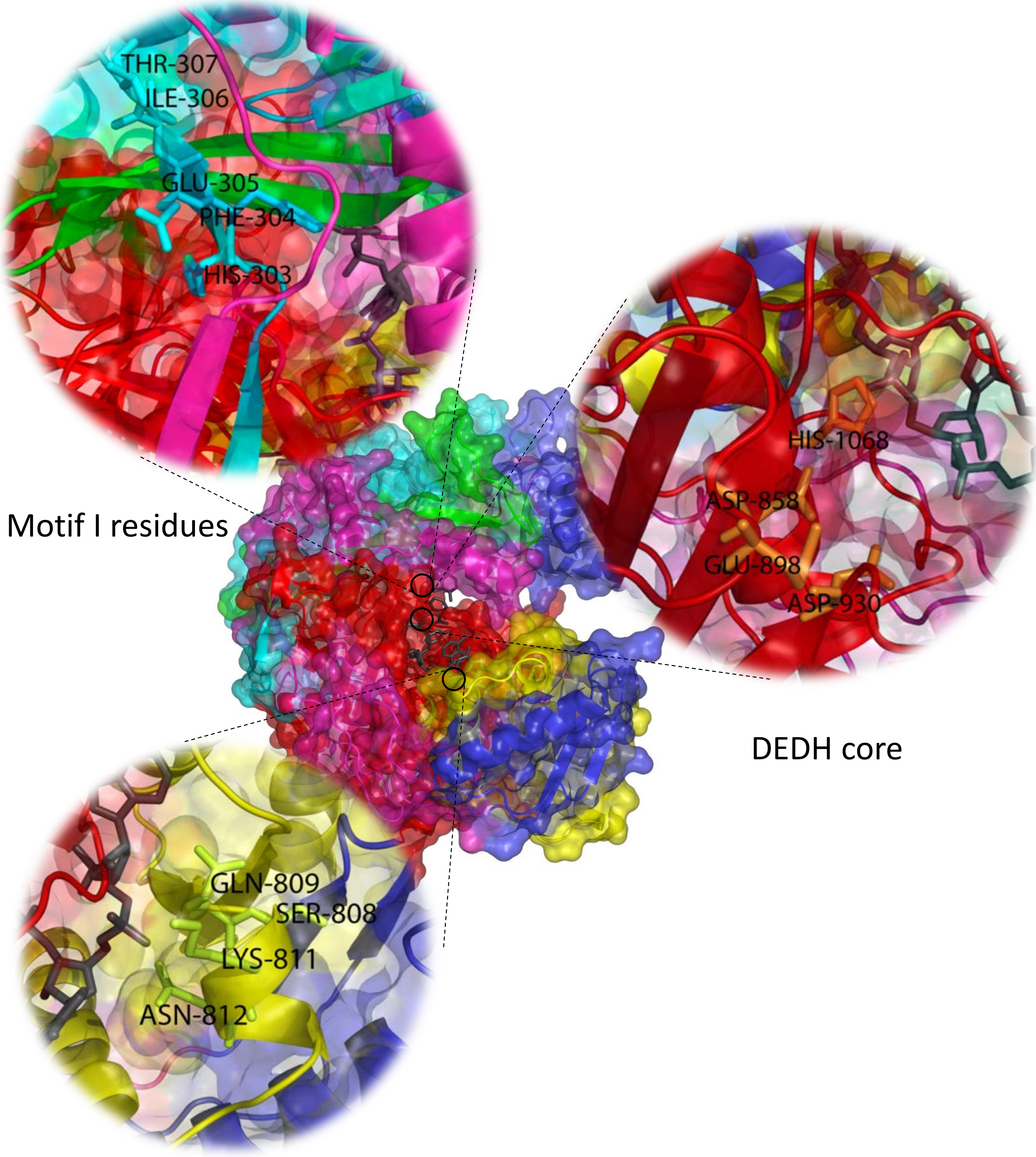

THR-307
ILE-306
GLU-305
PHE-304
HIS-303
Motif I residues
HIS-1068
ASP-858
GLU-898
ASP-930
DEDH core
GLN-809
SER-808
LYS-811
ASN-812
Seed binder

I

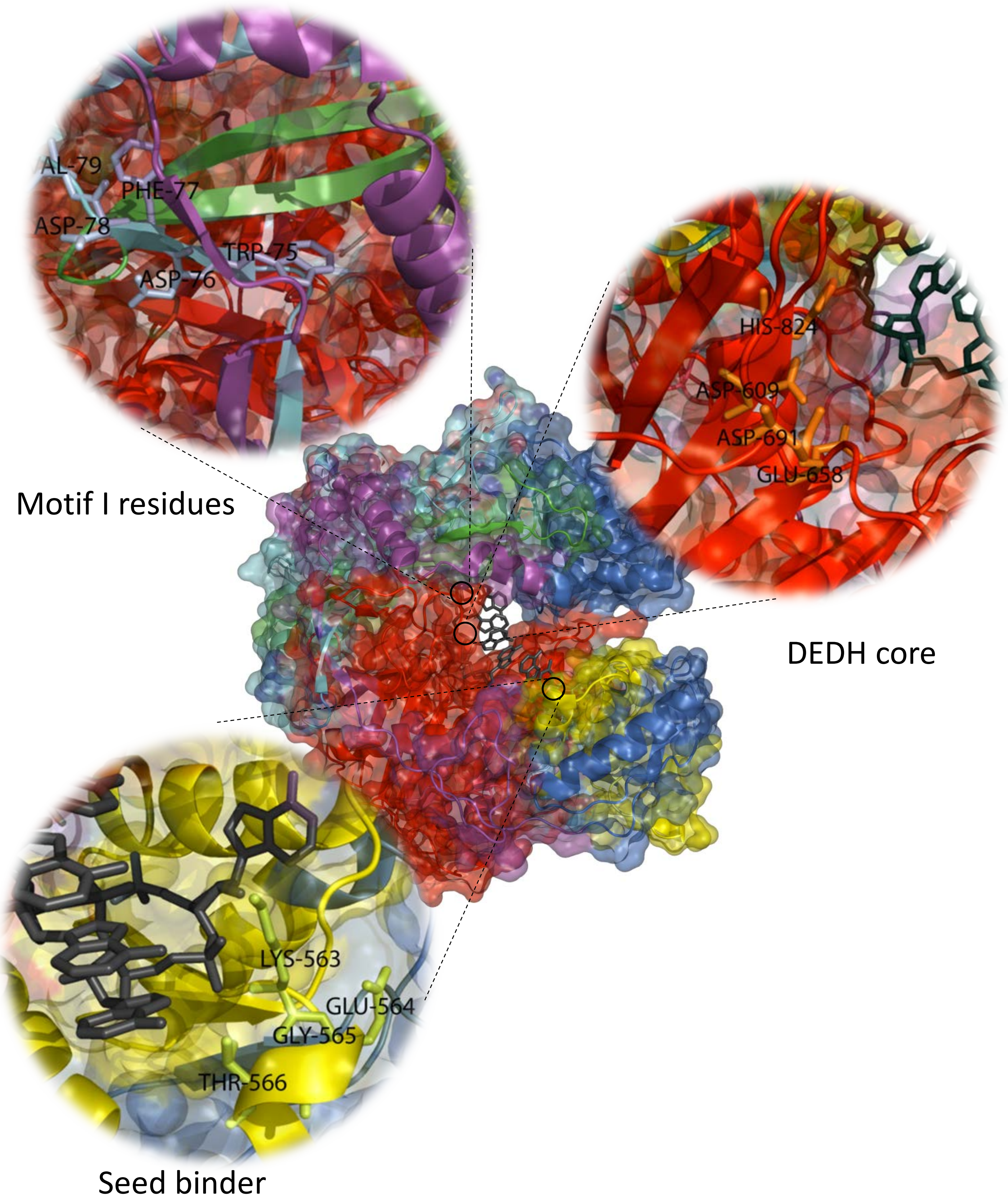

AL-79
PHE-77
ASP-78
TRP-75
ASP-76
Motif I residues
HIS-824
ASP-609
ASP-691
GLU-658
DEDH core
LYS-563
GLU-564
GLY-565
THR-566
Seed binder

J

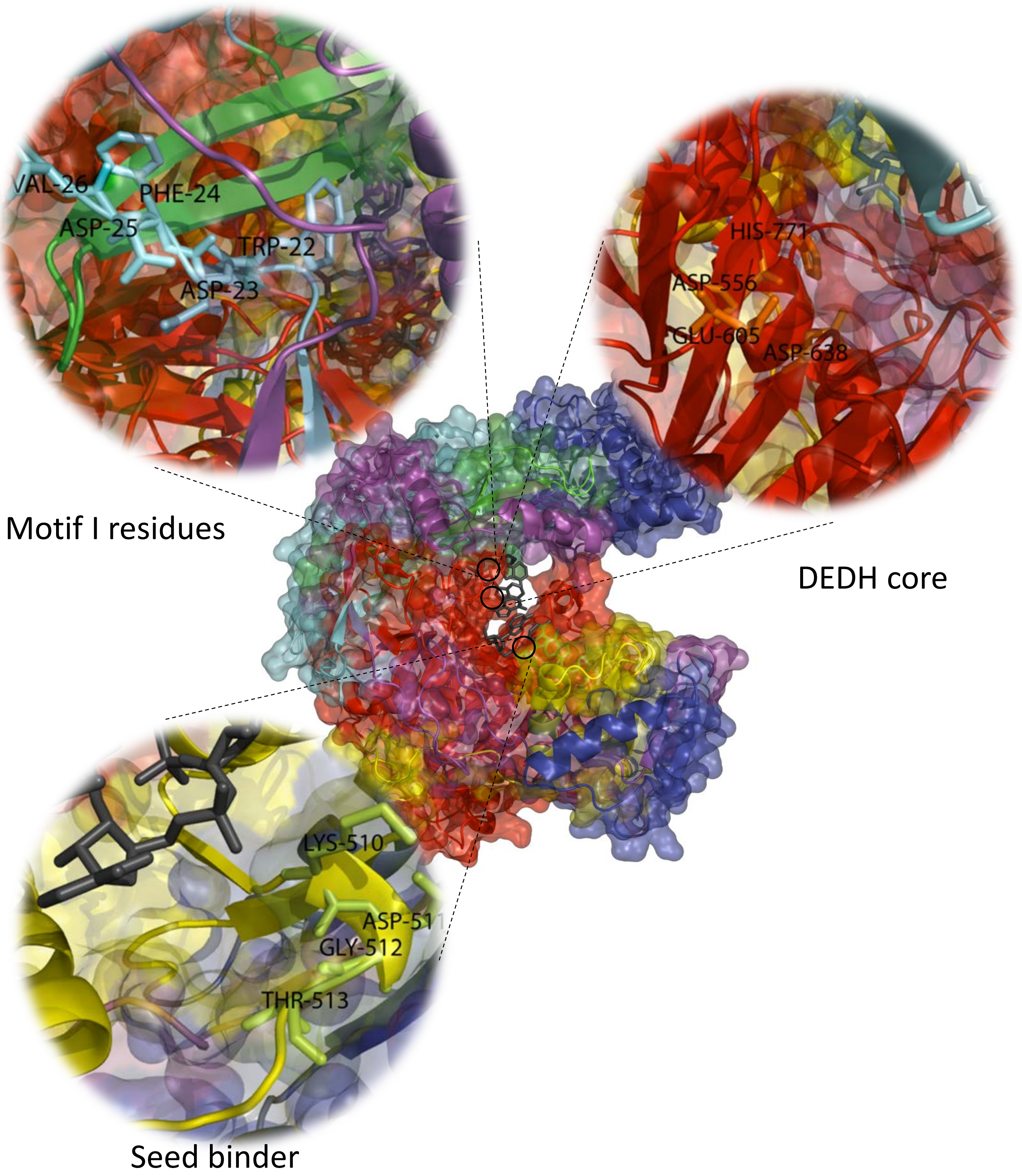

VAL-26
PHE-24
ASP-25
TRP-22
ASP-23
HIS-771
ASP-556
GLU-605
ASP-638
LYS-510
GLY-512
THR-513
Motif I residues
DEDH core
Seed binder

K

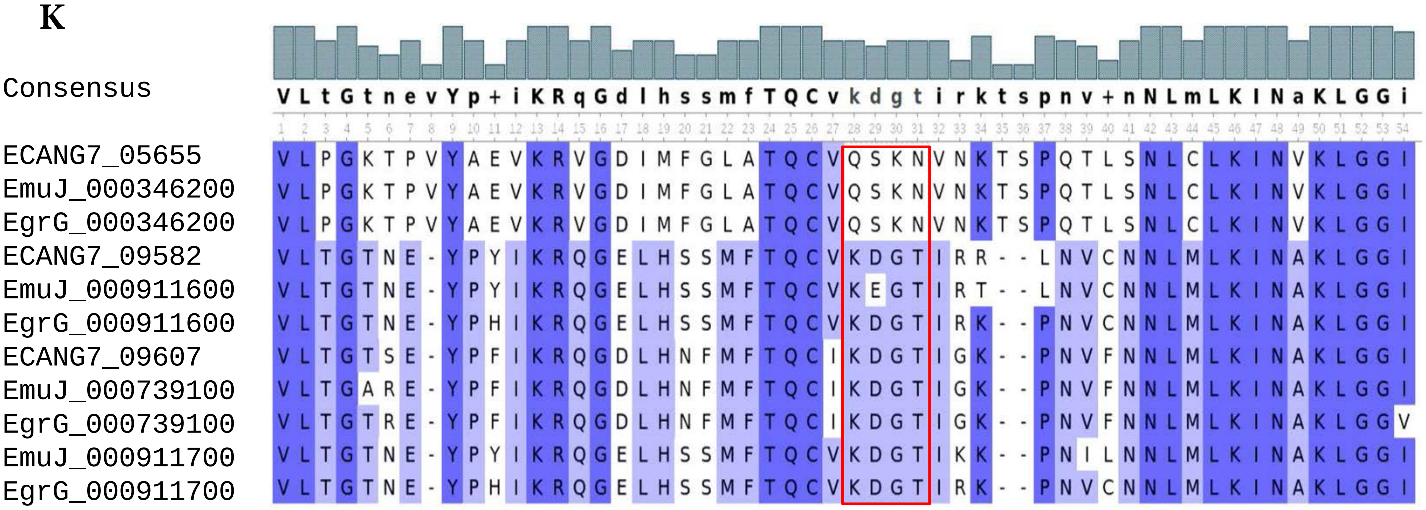

L

Ago primers RT-PCR

Primers sequence used for RT-PCR

Genes	Sequence (5'-3')	Annealing temperature (°C)	Product size (pb)
EchiAgoF1_346200	CAAGTGGGTCAAGAGCAGAA	51.78	**100**
EchiAgoR1_346200	AGGTCTGCATGTCAGTGAGC	53.83	
EchiAgoF2_911600	AAATTCGGATTGGGAATGAG	47.68	97
EchiAgoR2_911600	GTACCAGCTCCCCCTATCAA	53.83	
EchiAgoF4_739100	GCAGATCGTTTTGGGAATTT	47.68	143
EgrAgoR4_739100	AAAGTGAGCACTCGGTTGTG	51.78	
EgrAgoF5_911700	GGAGGAACAATTGGGAGAAA	47.68	67
EchiAgoR5_911700	ACACATCCGATACGTCGAAA	49.73	
eif_F	TCGGGACAAGAGGGTAGAGA	53.83	108
eif_R	ACTAACAGCGGAAGGCGTAA	51.78	
actin_190400_F1	ACGGGTATCGTCTTGGACTC	53.83	105
actin_190400_R1	CACGACCAGCTAAATCGAGA	51.78	
