# Supplementary material for: The Echinococcus canadensis (G7) genome: a key knowledge of parasitic platyhelminth human diseases
Source: BMC Genomics. 2017 Feb 27;18:204. doi: 10.1186/s12864-017-3574-0 (PMC5327563; doi:10.1186/s12864-017-3574-0)
Supplement: Additional file 10: — Global phylogeny of Echinococcus and model species. (A) Phylogeny reconstruction from complete Echinococcus mitochondrial genomes. Nucleotide sequences of 12 protein-coding genes were aligned according to Nakao et al. [53]. Phylogenetic analysis was performed using the Maximum Likelihood method based on the JTT matrix-based model. Bootstrap consensus tree was inferred from 100 replicates. Branches corresponding to partitions reproduced in less than 50% bootstrap replicates are collapsed. Mitochondrial genomes used for Echinococcus species phylogeny reconstruction: E. oligarthrus, NC_009461, Nakao et al., [108]; E. vogeli, NC_009462, Nakao et al., [108]; E. equinus, AB786665, Nakao et al., [53]; E. granulosus, AB786664, Nakao et al., [53]; E. felidis, AB732958, Nakao et al., [53]; E. multilocularis, NC_000928, Nakao et al., [109]; E. shiquicus, NC_009460, Nakao et al., [108]; E. ortleppi, NC_011122, Nakao et al., [108]; E. canadensis (G6), NC_011121, Nakao et al., [108]; E. canadensis (G7), AB235847, Nakao et al., [108]; E. canadensis (G7), PRJEB8992, this work; E. canadensis (G8), AB235848, Nakao et al., [108]; E. canadensis (G10), AB745463, Nakao et al., [53]. (B) Proteins encoded by single-copygenes were analysed using the Maximum Likelihood method based on the JTT matrix-based model. The analysis involved 14 amino acid sequences from 29 single-copy genes. There were a total of 7001 positions in the final dataset. (C) Single-copy coding DNA sequences (CDS). The analysis involved 14 nucleotide sequences from 29 single-copy genes using the Maximum Likelihood method based on the Tamura-Nei model. There were a total of 14,364 positions in the final dataset. All of the positions with less than 60% site coverage were eliminated. Evolutionary analyses were conducted in MEGA5. Hsap: Homo sapiens; Mmus: Mus musculus; Drer: Danio rerio; Bflo: Branchiostoma floridae; Dmel: Drosophila melanogaster; Cele: Caenorhabditis elegans; ECANG7: Echinococcus canadensis; Egra: Echinococ [file 12864_2017_3574_MOESM10_ESM.pdf]

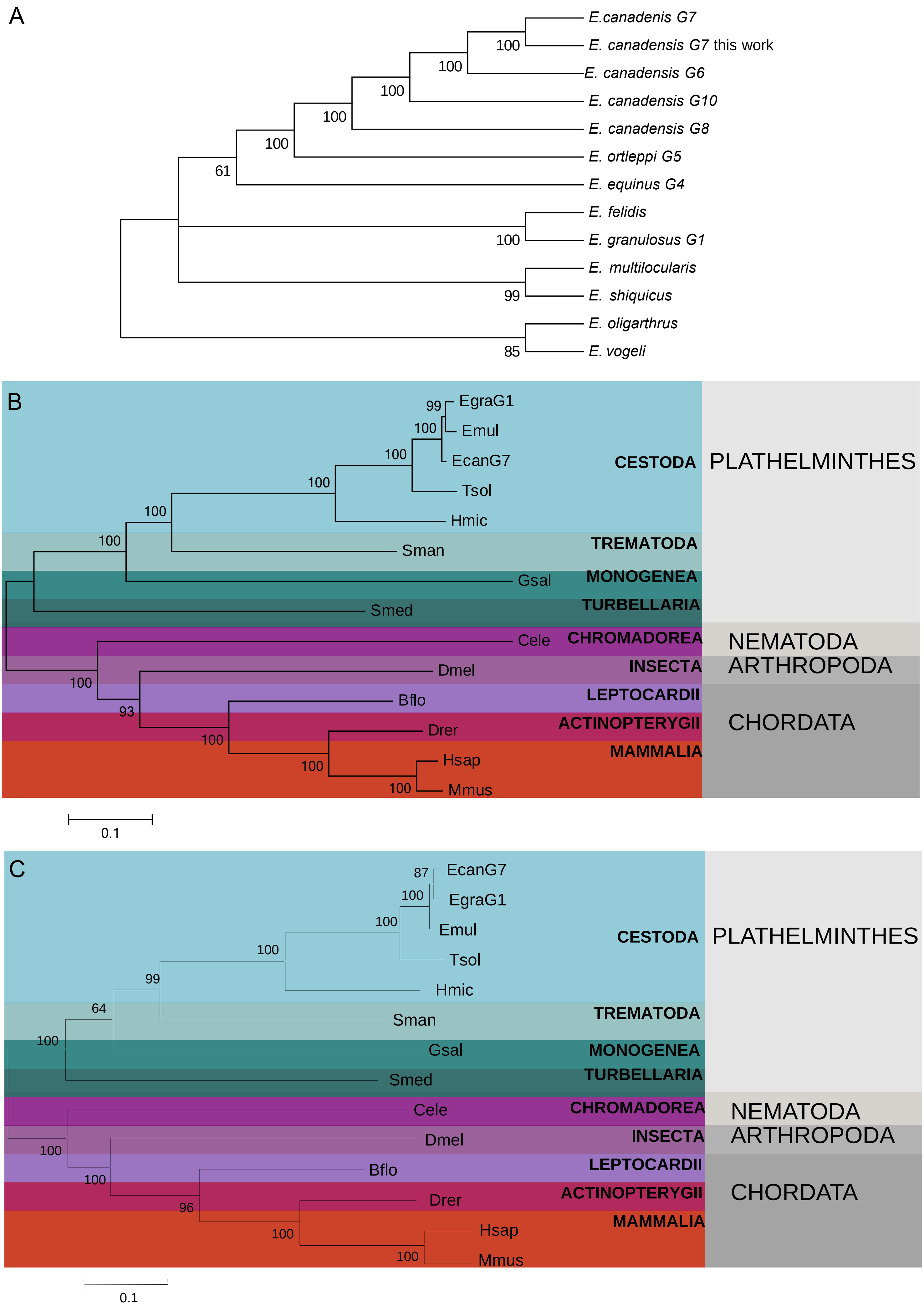
A
E.canadenis G7
E. canadensis G7 this work
E. canadensis G6
E. canadensis G10
E. canadensis G8
E. ortleppi G5
E. equinus G4
E. felidis
E. granulosus G1
E. multilocularis
E. shiquicus
E. oligarthrus
E. vogeli
100
100
100
100
100
61
100
99
85
B
EgraG1
Emul
EcanG7
Tsol
Hmic
Sman
Gsal
Smed
Cele
Dmel
Bflo
Drer
Hsap
Mmus
CESTODA
TREMATODA
MONOGENEA
TURBELLARIA
CHROMADOREA
INSECTA
LEPTOCARDII
ACTINOPTERYGII
MAMMALIA
PLATHELMINTHES
NEMATODA
ARTHROPODA
CHORDATA
0.1
C
EcanG7
EgraG1
Emul
Tsol
Hmic
Sman
Gsal
Smed
Cele
Dmel
Bflo
Drer
Hsap
Mmus
CESTODA
TREMATODA
MONOGENEA
TURBELLARIA
CHROMADOREA
INSECTA
LEPTOCARDII
ACTINOPTERYGII
MAMMALIA
PLATHELMINTHES
NEMATODA
ARTHROPODA
CHORDATA
0.1

D

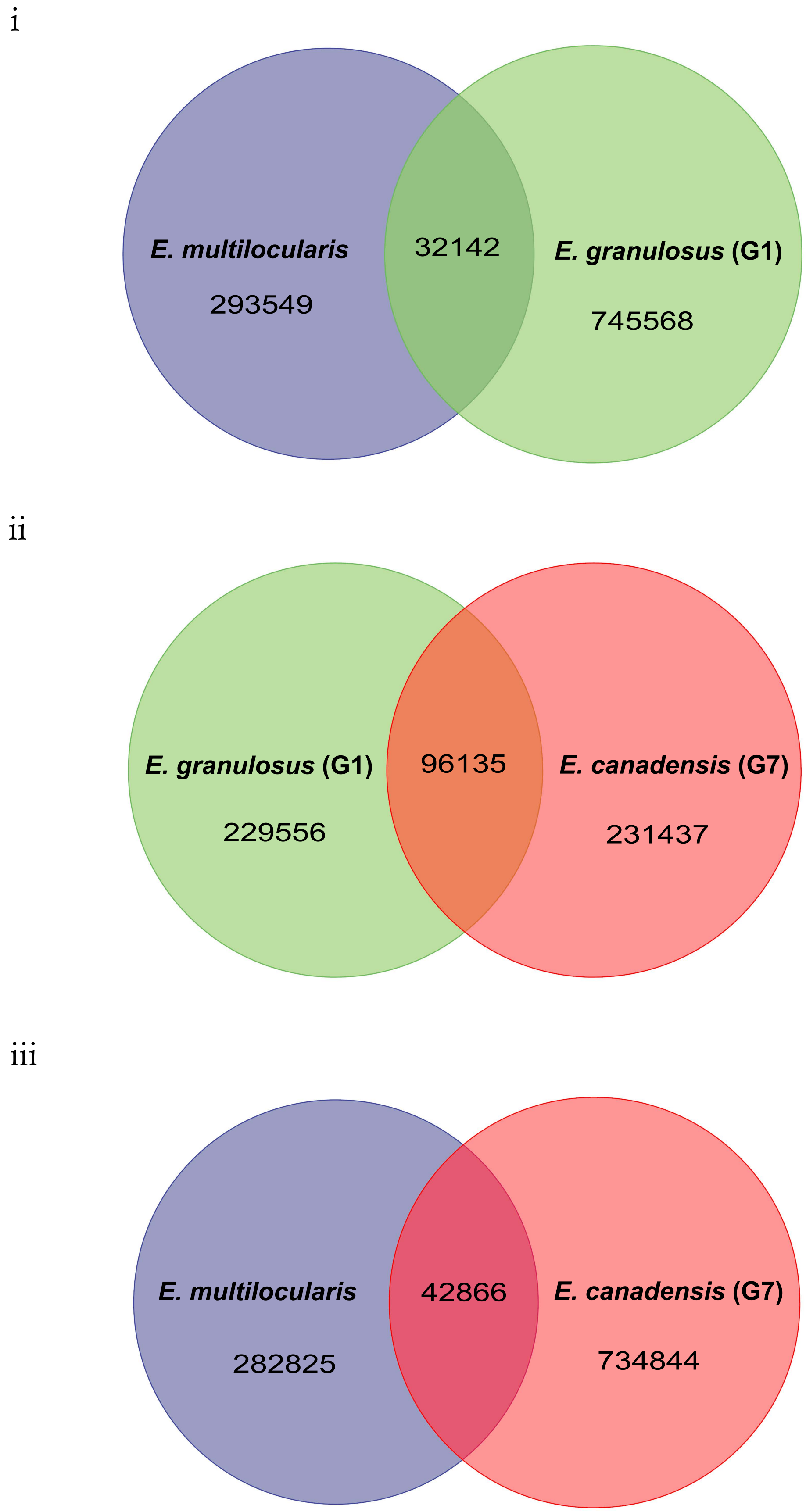
i
E. multilocularis
293549
32142
E. granulosus (G1)
745568
ii
E. granulosus (G1)
229556
96135
E. canadensis (G7)
231437
iii
E. multilocularis
282825
42866
E. canadensis (G7)
734844
